# Excursions in the Bayesian treatment of model error

**DOI:** 10.1371/journal.pone.0286624

**Published:** 2023-06-02

**Authors:** L. Mark Berliner, Radu Herbei, Christopher K. Wikle, Ralph F. Milliff

**Affiliations:** 1 Department of Statistics, The Ohio State University, Columbus, OH, United States of America; 2 Department of Statistics, University of Missouri, Columbia, MO, United States of America; 3 Cooperative Institute for Research in Environmental Sciences, University of Colorado, Boulder, CO, United States of America; Federal University of Pernambuco: Universidade Federal de Pernambuco, BRAZIL

## Abstract

Advances in observational and computational assets have led to revolutions in the range and quality of results in many science and engineering settings. However, those advances have led to needs for new research in treating model errors and assessing their impacts. We consider two settings. The first involves physically-based statistical models that are sufficiently manageable to allow incorporation of a stochastic “model error process”. In the second case we consider large-scale models in which incorporation of a model error process and updating its distribution is impractical. Our suggestion is to treat dimension-reduced model output as if it is observational data, with a data model that incorporates a bias component to represent the impacts of model error. We believe that our suggestions are valuable quantitative, yet relatively simple, ways to extract useful information from models while including adjustment for model error. These ideas are illustrated and assessed using an application inspired by a classical oceanographic problem.

## Introduction

Studies of model error have long-played a fundamental role in statistical modeling. This is especially evident in regression analysis. Also, at least since 1960, contributions of Kalman, Stratonovich, and others have provided powerful approaches for prediction of dynamical systems in the presence of noise; see the classic book [1] for discussion and references. More recently, technological advances have led to substantial research on the combination of information from both massive datasets and computer models, to produce effective inferences. It is important that inferences are accompanied by reliable measures of uncertainty. With these goals in mind, we develop approaches for treating model error for prediction of space-time processes.

We focus on Bayesian treatments of model error. We believe that the Bayesian approach generally offers the superior method for quantifying and communicating uncertainty. In addition, the use of observational data is fundamental to the assessment and treatment of model error and Bayesian statistics offers a powerful strategy for data analysis. We believe that physically-based models are critical sources of information regarding the processes of interest. Hence, it is important that analyses maintain the influence of such *prior* information. The Bayesian approach is an ideal framework for combining data and models.

### Notions of error

The term “error” is used in many contexts and with numerous meanings. We discuss two broad forms of error, namely, *measurement error* and *model error*. Here, “model” refers to mathematical objects that serve as primary tools for interpretation, inference, simulation, and prediction. Models may be deterministic or probabilistic (statistical), or some combination of these. There is a spectrum of complexity and a variety of uncertainties.

Measurement error is pervasive in the production of data. The common informal definition is “measurement error = observation—true value”. Similarly, in numerical modeling we have “model error = numerical result—true value”. This definition applies to numerical models that are computable in closed form as well as those that only provide computer model output. Other names for model error include *discrepancy, bias*, and *offset*.

The informal definitions of error above must be generalized in problems for which simple additive errors are insufficient. Further, the usual goal in probabilistic modeling is the formulation of a probability distribution for quantities of interest that matches the true probability distribution of those quantities. We offer a clarification in use of the term “error”. Typically, statisticians use the term to refer to misspecifications of probability distributions used in an analysis. This may lead to phrases like “model error in the specification of the distribution of model error”. We merely point this issue out and trust that interpretations in specific examples will be clear.

The casual definition of measurement error above is often an oversimplification. Specifically, quantities viewed as observational data may be results of some numerical model, itself subject to model error. Very important examples arise in remote sensing. Typically, remote sensors record electromagnetic radiation (or photon counts). Models are applied to that raw information to produce “observations” of quantities of interest (e.g., [2–4]). In many cases, use of statistical summaries of observations in the production of observational data may introduce a variety of errors or biases. Though this is an important topic, we focus on computer model error here.

There are two primary sources of computer model error. First, errors arise due to numerical model inadequacies, such as those resulting from approximations (e.g., “ignoring higher order terms”), as well as algorithmic or computer mistakes. The second category comprises *apparent* model errors that arise due to misspecification of model inputs. Most numerical models require specification of parameters or processes, such as boundary or initial conditions. Errors in such inputs may produce model output that is in error, even for virtually error-free models. (We discuss this issue further in Section 4.) These points are related to *intrinsic* versus *extrinsic* error classifications. Also, the issues are related to research known as *uncertainty quantification* (UQ). In our framework inputs are modeled as random variables with distributions that reflect uncertainties and errors. Hence, the output of even deterministic models would be treated as random. Also, the output of very high-dimensional and complex, including chaotic, models may often be usefully treated as random.

There are several statistical and inference problems associated with model error. Seminal contributions regarding the design and analysis of computer experiments include [5, 6]. Other examples are model assessment such as verification and validation [7–9]. Calibration is important for the eventual use of the model [10]; [11]. Models which display satisfactory assessments and are well-calibrated may be used to obtain large ensembles to learn about responses to variations of parameters, etc. This notion is highly related to *model emulation* [12–14]. In this article we focus on prediction.

### Bayesian modeling overview

We use **Y** to denote observational data, **X** to denote the processes that are the main targets of our analyses, and ***θ*** to denote unknown parameters. Bayesian analysis is based on probability modeling for all such quantities. For random objects, say *U* and *V*, we use the notation [*U*, *V*] to denote the joint distribution of *U* and *V*, [*U*|*V*] to denote the conditional distribution of *U* given *V*, and [*U*] to denote the marginal distribution of *U*. The following skeleton of a Bayesian model is useful in formulating models [15]. The model consists of three primary probability distributions:

1. *Data Model*: [**Y** | **X**, ***θ***_*y*_]. The data model is the conditional probability distribution of **Y** given **X** and perhaps a collection of unknown parameters ***θ***_*y*_.
2. *Process Model Prior*: [**X** | ***θ***_*x*_]. The process model prior is a probability distribution for the processes of interest, perhaps depending on some parameters ***θ***_*x*_.
3. *Parameter Model Prior*: [***θ***_*y*_, ***θ***_*x*_]. Bayesian modeling is completed by specifying a prior distribution of all unknown parameters.

After observing **Y**, the data model is combined with the process and parameter models via Bayes’ Theorem to produce the posterior distribution

$$\begin{array}{c} \left[X,\theta_{y},\theta_{x}\;|\;Y\right]\propto \left[Y\;|\;X,\theta_{y}\right]\;\left[X\;|\;\theta_{x}\right]\;\left[\theta_{y},\theta_{x}\right]. \end{array}$$ (1)

The specification of all three of the model components are subject to *modeling error*. In this article we focus on the treatment of error in specifying and using process model priors. In our development below, physical models are components of process model priors. There is substantial research on modeling error regarding data models and parameter priors; [16, 17] provide substantial discussion, also see [18]. We note that there are challenges regarding the impacts and interactions of model errors among all three of the primary components of a Bayesian model. Though very important, this issue is beyond the scope of this article. Discussion and references regarding robust Bayesian analysis are given in [19, 20]. Realistic models are often complicated and involve many more unknown quantities than indicated in the descriptions of the distributions involved in Eq (1). For example, **X** may include spatial-temporal fields of several variables. Bayesian hierarchical modeling (BHM) provides tools for developing complex models, clarifying assumptions, and quantitative uncertainty management. A hierarchical probability model for a collection of random variables is a sequence of conditional probability models that specifies a bona fide joint probability distribution. The product of the three distributions on the right hand side of Eq (1) is an example of a BHM, but is also deceptively simple compared to the sort of models that arise in practice.

### Physical-statistical models

Historically, many analysts separated modeling into two categories: statistical modeling and physically-based mechanistic modeling. In this article we combine the two notions by developing physical- (or mechanistic-) statistical models (e.g., [21]). To clarify, suppose **X** represents a spatial-temporal field. Our starting point for the mechanistic-model contribution is consideration of a deterministic dynamic model for **X** in continuous time *t*,

$$\begin{array}{c} \frac{\partial X}{\partial t}=h\left(X;Z,\theta_{z}\right) \end{array}$$ (2)

where **Z** is a collection of relevant processes and ***θ***_*z*_ are parameters that arise in the modeling. Important examples of **Z**, particularly in prediction, are known as *explanatory* or *independent* or *predictor* variables or *covariates*. Further, **Z** may include quantities such as initial and/or boundary conditions, forcings, etc. Despite the importance of **Z**, we assume it and ***θ***_*z*_ are known so that we can focus on model error. We return to consideration of **Z** in the Conclusions. Till then, we suppress dependence on **Z** and ***θ***_*z*_. Also, many models are discrete time and/or space models. Important examples are computationally implemented approximations of (2). For brevity, we do not rewrite continuous models as discrete ones in the general discussion.

We suppose that *nature* uses a “true model” to produce a “true” space-time process $\left\{\tilde{X}\left(s,t\right):\left(s,t\right)\in D\right\}$ where D denotes the space (*s*)—time (*t*) domain being considered. However, the true model is unknown to us. Further, we cannot observe $\tilde{X}$ exactly but only subject to measurement error. Our use of the notation **X** rather than $\tilde{X}$ is a response to such limitations. Relying in part on the model in (2), we formulate a *physical-statistical model*

$$\begin{array}{c} g\left(\left(X,\eta ,\theta_{x}\right)\left(s,t\right)\right)=0\;,\;\;\left(s,t\right)\in D\subset \tilde{D}\;, \end{array}$$ (3)

where ***η*** is a stochastic process used to represent error in the specification of **g** and ***θ***_*x*_ are additional unknown, random parameters that arise in the modeling. Our goal is to formulate useful statistical models or otherwise manage the impacts of ***η***.

Note that {η(s,t)∣(s,t)∈D} is our *modeled* model error process. We define the *true model error process* as

$$\begin{array}{c} \delta \left(s,t\right)=\tilde{X}\left(s,t\right)-X\left(s,t\right),\;\left(s,t\right)\in D. \end{array}$$ (4)

We have emphasized the differences between $\tilde{X}$ and **X** and between ***δ*** and ***η***. This is rarely done in the literature and hereafter we use **X**, not $\tilde{X}$, to denote the quantity of interest and in specifying the data model and the process model prior. We reserve the use of $\tilde{X}$ in the specification of the “true process” in our numerical examples in the “Illustrations” Section. Similarly, our probability model for ***η*** may be viewed as our prior distribution for ***δ***, though only ***η*** is used in the rest of this article.

## Goals and strategies

Our goal in forming a *prediction* analysis is to combine observational data and computer model output in a fashion that manages (and reduces, when possible) model error and quantifies uncertainty. Statistical contributions to our discussion arise due to (i) use of observational data, (ii) the Bayesian treatment of all unknowns including fixed parameters as if they are random variables, and (iii) treatment of model errors as stochastic processes. Related research include [6, 22].

Note that the third point is relevant even in the use of output from deterministic models. Descriptions of our approaches are readily explained for a common collection of models; namely, models with *additive errors*. For example, a typical data model is of the form

$$\begin{array}{c} Y=X+∊ \end{array}$$ (5)

where ***∊*** represents zero-mean measurement errors. Connecting to the general notation, Eq (5) is interpreted as specifying that conditional on **X**, the expectation of **Y** is **X**, i.e., the observations are *conditionally unbiased*. The data model [**Y** | **X**, ***θ***_*y*_] specifies the distribution, e.g., normal (Gaussian), of ***∊***; ***θ***_*y*_ could be the covariance matrix of ***∊***.

We organize the presentation of model-error treatments around two classes of models based primarily on practical constraints. We intend no hard delineation between these classes, nor are the classes exhaustive. Also, analyses may feature aspects of both classes.

### Class I

We suppose the models as in Eq (3) are sufficiently manageable to allow incorporation of a model error process into the dynamics or an approximate model based on those dynamics (e.g., discretization of a continuous model). The key points are that we can formulate probabilistic models for the model errors ***η*** and use observational data to compute updates of all features of the Bayesian model.

In [23] the authors considered prediction of tropical surface winds given high-resolution satellite (i.e., scatterometer) observations and low-resolution assimilated model output obtained from the National Centers for Environmental Prediction. The authors applied physical-statistical modeling at multiple scales using both a solution to an approximating system of differential equations (“shallow fluid equations”) and multi-resolution wavelets. The resulting space-time model for the north-south components **v**_*t*_ of surface winds is

$$\begin{array}{c} v_{t}=\mu +\Phi a_{t}+\Psi b_{t} \end{array}$$ (6)

where ***μ*** is a mean, Φ is a matrix containing basis functions of the differential equations and Ψ contains wavelet basis functions (the model for the east-west components of the winds is analogous). They formed a linear dynamic model for the coefficients **a**_*t*_:

$$\begin{array}{c} a_{t}=H\left(\theta \right)a_{t-1}+\eta_{t} \end{array}$$ (7)

the ***η*** process represents model error in the *spectral domain* (a similar model was used to model the **b**_*t*_’s). In the spirit of Class I, model error represented by ***η*** is incorporated directly into the (transformed) physical-statistical model.

An example in the context of glacial dynamics is provided in [24]. They include model error in a physical model that improves model behavior. Also, they are able to offer scientific interpretations of the posterior distribution of that error suggesting spatial regions where the physical model requires adjustment.

In [25] the authors use a BHM for surface wind forcing within a variational data assimilation scheme (weak constraint) to identify regions in (*s*, *t*) of suspected model error for a high-resolution model of the California Current System upwelling/downwelling processes. Model error identification leads to improved model forecasts.

### Class II

We suppose that direct incorporation of dynamical, stochastic error processes is not practical. Examples arise when the quantities modeled are of very high dimension. Specifically, suppose that **X** is a vector of length in the 10’s to 100’s of millions. Though we can “write down” Eq (3), we believe that usefully modeling and subsequently learning about the error process ***η*** from data in such large dimensions is implausible. Similar concerns may arise in low to moderately sized problems involving complicated and computationally expensive models. (The meanings of “large” dimensions and “implausible” and/or “computationally expensive” evolve as both data collection and computational assets improve.) However, our challenge remains the use of computer model output in a fashion that accounts for model error. The strategy pursued in this article treats computer model output as *biased observations of the truth*. That is, we will formally model output in much that same fashion as we treat observational data.

Let G(X) be a dimension-reduced summary of **X**. For example, the operator G may be a matrix whose elements indicate the selection of specific subsets of variables, spatial and/or temporal averaging of **X**, etc. Even in moderate dimensions we often must settle for comparing estimated expectations (means, probabilities, information measures, etc.) and other summary statistics such as medians, coverings, etc. Let **Y**^*c*^ represent the corresponding summary derived from model output. The superscript “c” emphasizes the fact that these “data” are computer generated as opposed to observational data **Y**. We incorporate the computer output based on a statistical model of the form

$$\begin{array}{c} Y^{c}=\beta +\vec{G}\left(X\right)+\gamma \end{array}$$ (8)

where ***β*** represents *bias* that arises as an unknown function of the large-scale model error ***η***, $\vec{G}$ is the appropriate vectorized form of G(X), and **γ** is a vector of zero-mean errors with covariance matrix *Σ*^*c*^. For clarity, in numerical simulated examples presented later, we actually produce $Y^{c}$ as

$$\begin{array}{c} Y^{c}=\vec{G}\left(\tilde{X}\right)+∊. \end{array}$$ (9)

We note that it is possible to formulate G and $\vec{G}$ as functions of unknown parameters. While we envision an underlying model (3) and the existence of a function that maps ***η*** to ***β***, we assume that estimating this function is implausible. Hence, our fundamental modeling challenge is to formulate useful statistical models for ***β*** directly. Note that this means the form and behavior of ***β*** vary with the choice of G. We note that the dimension-reduction strategy may induce a further error. We don’t account for this separately, but rather assume that this is incorporated in ***β***.

By treating model output as if they are observations, we mean that we assume **Y**^*c*^ has a conditional distribution [**Y**^*c*^ | **X**, ***β***, ***θ***_*y*^*c*^_]. Note that the covariance matrix *Σ*^*c*^ of the errors **γ** in Eq (8) is an example of ***θ***_*y*^*c*^_. Assuming that the observational data, now denoted by **Y**^*o*^ for clarity, and the model output are conditionally independent, the BHM that combines observations and dimension-reduced model output is summarized as

$$\begin{array}{ccc} & \text{data}\;\text{model} & \left[Y^{o}\;|\;X,\theta_{y^{o}}\right]\;\left[Y^{c}\;|\;X,\beta ,\theta_{y^{c}}\right] \end{array}$$ (10)

$$\begin{array}{ccc} & \text{process}\;\text{model} & \left[X,\beta \;|\;\theta_{x},\theta_{\beta }\right] \end{array}$$ (11)

$$\begin{array}{ccc} & \text{parameter}\;\text{prior} & [\theta_{x},\theta_{y^{o}},\theta_{y^{c}},\theta_{\beta }]. \end{array}$$ (12)

Formulation and interpretation of the priors in this approach merit further discussion. First, though we have written the process model prior as a conditional probability distribution for **X**, it may be the case that we only form a prior probability model for the low dimensional summary variable G(X). Next, an important by-product of this strategy is that it offers opportunities to combine simplified, low-dimensional physical-statistical models or purely statistical models for G(X) with output from large-scale computer models. *Downscaling* is one potential application. An example is reviewed at the end of Section 2.3. The dimensions of **Y**^*o*^, **Y**^*c*^ may also be too large to be usable “as is”. This potentially requires introducing a further operator, acting on these variables, generally performing another level of dimension reduction. Though very important, we do not pursue these points further here. Rather, we assume that dimension reduction is performed on **X** only. Finally, forming priors on parameters is a common, though not typically easy, task in Bayesian analysis. Here, we also face the problem of formulating a prior probability distribution for ***β***. Though challenging, this problem may also be worth the effort. Data-informed posterior distributions for biases may help us to understand model error and suggest possible improvements to models. Also, note that we have included ***β*** in the prior process model and allowed for additional flexibility by using a distribution that may be parameterized by parameters ***θ***_*β*_. This is particularly useful when incorporating more than one numerical model (see the next section).

In practice additive-errors data models such as (5) and (8) may be inappropriate. Hence, the general probabilistic notation above is important in formulating models and performing computations.

### Multiple computer models

This approach offers a method for combining model output from multiple models (e.g., [26]). Suppose we are given model output from *K* computer models. Our model data consists of *K* vectors $Y^{c_{1}},\ldots ,Y^{c_{K}}$ of length *J*_1_, …, *J*_*K*_, respectively. Following (8) we assume that

$$\begin{array}{c} Y^{c_{k}}=\beta_{k}+{\vec{G}}_{k}\left(X\right)+\gamma_{k},\;k=1,\ldots ,K. \end{array}$$ (13)

Note that we have allowed each model to suffer from its own biases. Further, the ${\vec{G}}_{k}\left(X\right)$ vary with the computer model, may be of differing dimensions, and even different variables in **X**. If maintaining the conditional independence assumption is reasonable, the BHM extends to

$$\begin{array}{cc} \text{data}\;\text{model} & \left[Y^{o}\;|\;X,\theta_{y^{o}}\right]\;\left[Y^{c_{1}}\;|\;X,\beta_{1},\theta_{y^{c_{1}}}\right]\;\ldots \;\left[Y^{c_{K}}\;|\;X,\beta_{K},\theta_{y^{c_{K}}}\right] \end{array}$$ (14)

$$\begin{array}{cc} \text{process}\;\text{model} & \left[X,\beta_{1},\ldots ,\beta_{K}\;|\;\theta_{x},\theta_{\beta_{1}},\ldots ,\theta_{\beta_{K}}\right] \end{array}$$ (15)

$$\begin{array}{cc} \text{parameter}\;\text{prior} & [\theta_{x},\theta_{y^{o}},\theta_{y^{c_{1}}},\ldots ,\theta_{y^{c_{K}}},\theta_{\beta_{1}},\ldots ,\theta_{\beta_{K}}]. \end{array}$$ (16)

The conditional independence assumptions among the various models does not mean the model outputs are marginally independent. Indeed, we expect some similarities among the models. Rather, it means that model output departures {**γ**_*k*_} from the means $\beta_{k}+{\vec{G}}_{k}\left(X\right)$ when **X** and the biases are known are independent between the models. Further, suppose the $G_{k}$ all represent the same summary of the large-scale process. We then have the opportunity of building similarities of the models by appropriate formulation of the priors for ***β***_*k*_.

Berliner and Kim [27] (hereafter BK) provides a Bayesian analysis for process variables **X** defined as hemispheric- and monthly averaged surface temperatures for the period 1882–2097. They used observational data over the period 1882–2001 to form a statistical time series model for the temperatures. Climate model projections obtained from two climate system models were incorporated as biased observations to drive the model for the period 2002–2097. A key challenge in their case is that the prior for the model biases β_*_(*s*, *t*) should accommodate their temporal evolution. In some cases, analysts may have sufficient prior information to construct meaningful time series models for the biases. BK assumed very little prior information. In their comparatively uninformed analysis they assumed that the biases were constants locally in time. A single bias assumed over the entire time period seems implausible and could lead to overconfidence in the final conclusions. At the other extreme biases modeled to change at each time point, but are otherwise unstructured, would lead to very weak inference about the biases. Based on some preliminary data analysis, BK assumed that the biases are constant over intervals 2002–31, 2032–51, 2052–71, 2072–91, and 2092–97. Note that the observational data is used to form a process model prior [**X** | ***θ***_*x*_] for prediction of **X** after 2001. In a “real” analysis, we would seek observations and model output that overlap in time. This could improve inferences for model biases.

## Illustrations: Advection-diffusion equation

**Fig 1:**
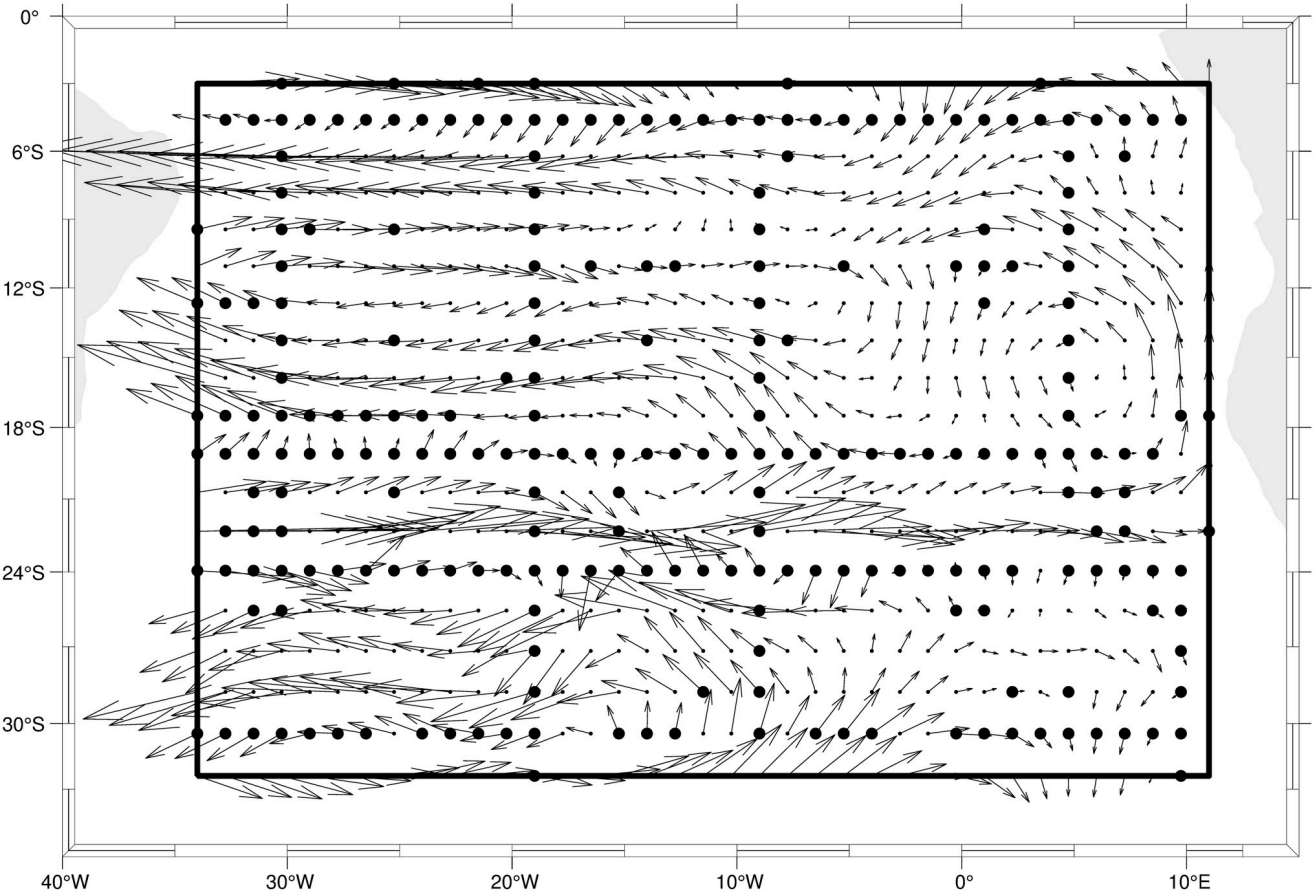
The domain Ω used in Eq (17). Arrows indicate the velocity field (*u*, *v*). The large black dots are locations where observations of the process **X** are available.

We illustrate the concepts using a physical model that is representative of those used in oceanography and other areas. Our first application is inspired by the inverse problem described in [28], although we use synthetic data. Let $\Omega \subset R^{2}$ be a rectangular domain (displayed in Fig 1) and let *X* = *X*(*x*, *y*;*t*) represent the concentration of a tracer at time *t* and location *s* = (*x*, *y*) in the domain Ω. The dynamics of *X*(⋅, ⋅, ⋅) are modeled using the advection-diffusion equation

$$\begin{array}{c} \left\{ \begin{array}{c} \frac{\partial X}{\partial t}=\frac{\partial }{\partial x}(\kappa_{1}\frac{\partial X}{\partial x})+\frac{\partial }{\partial y}(\kappa_{2}\frac{\partial X}{\partial y})-u\frac{\partial X}{\partial x}-v\frac{\partial X}{\partial y}-\lambda X\\ X_{\partial \Omega }=b. \end{array}\right. \end{array}$$ (17)

where (*x*, *y*) ∈ Ω, *t* ∈ [0, *T*] and (*u*, *v*) = (*u*(*x*, *y*), *v*(*x*, *y*)) are velocity fields and (*κ*_1_, *κ*_2_) = (*κ*_1_(*x*, *y*), *κ*_2_(*x*, *y*)) are diffusion coefficients. The consumption rate is *λ* > 0 and $b=\vec{b}\left(x,y\right)$ denotes the Dirichlet boundary values. We impose the incompressibility condition ∂*u*/∂*x*+ ∂*v*/∂*y* = 0. Note that Eq (17) cannot be solved analytically.

### Class I approach

#### True process

We generate the true process $\tilde{X}$ using a modification of the advection-diffusion equation. First, we use a version of the model that is discretized in both space and time. We defined spatial locations by their standard coordinates: (*x*, *y*). Fig 1 indicates a 19 × 37 = 703 regular grid on which our numerical approximation is computed for monthly times *t* = 0 to *T* = 20. The velocities (*u*(*x*, *y*), *v*(*x*, *y*)) (displayed in Fig 1) are inspired by the oceanographic application in [28]. The critical step in generating the true process $\tilde{X}$ here is that *diffusion coefficients* *κ*_1_*and* *κ*_2_ are modeled to *vary spatially*:

$$\begin{array}{c} \kappa_{1}\left(x,y\right)=a_{1}x+a_{2}\;\text{and}\;\kappa_{2}\left(x,y\right)=b_{1}y^{2}+b_{2}y+b_{3}. \end{array}$$ (18)

The constants *a*_1_, *a*_2_, *b*_1_, *b*_2_, *b*_3_ are selected such that *κ*_1_ and *κ*_2_ remain positive throughout the domain.

#### Observations

For this application we use simulated observations, whereas [28] used real data. In their case, Ω is a 2000*m* deep neutral density layer in the South Atlantic Ocean and they use a multivariate tracer concentration (oxygen, salinity, silica, etc.). At a subset of 335 sites (larger dots in Fig 1) and for the first 12 months, we generated observations as

$$\begin{array}{c} Y\left(s_{i},t_{j}\right)=\tilde{X}\left(s_{i},t_{j}\right)+\epsilon \left(s_{i},t_{j}\right), \end{array}$$ (19)

where the simulated measurement errors *ϵ*(⋅, ⋅) are all mutually independent, zero mean random variables with common variance $\sigma_{y}^{2}$.

#### Process model prior

We prescribe a prior model for the space-time field **X** as a discretized version of a stochastic advection-diffusion equation with additive model error ***η***:

$$\begin{array}{c} \frac{\partial X}{\partial t}=\kappa_{x}\frac{\partial^{2}X}{\partial x^{2}}+\kappa_{y}\frac{\partial^{2}X}{\partial y^{2}}-u\frac{\partial X}{\partial x}-v\frac{\partial X}{\partial y}-\lambda X+\eta \end{array}$$ (20)

where the diffusivities *κ*_*x*_ and *κ*_*y*_*are assumed to be constant* throughout the domain. This is a typical assumption in oceanographic applications [28], made, in part, due to the difficulty of estimating both the velocities and the diffusion coefficients as spatial fields. We used the same consumption rate *λ* and boundary conditions as in Eq (17). We compare results for four choices for the model error process ***η***:

$$\begin{array}{ccc} \eta_{1} & = & 0\;\text{everywhere}. \end{array}$$ (21)

$$\begin{array}{ccc} \eta_{2} & = & A\frac{\partial X}{\partial x}+B\frac{\partial X}{\partial y}\;;\;A,B\in R. \end{array}$$ (22)

$$\begin{array}{ccc} \;\eta_{3} & = & A\left(t\right)\frac{\partial X}{\partial x}+B\left(t\right)\frac{\partial X}{\partial y}\;;\;A\left(t\right),\;B\left(t\right)\text{are}\;\text{linear}\;\text{functions}\;\text{of}\;\text{time}. \end{array}$$ (23)

$$\begin{array}{ccc} \;\eta_{4} & = & \alpha_{1}\left(t\right)\frac{\partial X}{\partial x}+\alpha_{2}\left(t\right)\frac{\partial X}{\partial y}\;;\;\alpha_{1}\left(t\right),\;\alpha_{2}\left(t\right)\text{are}\;\text{AR}\;(1)\text{processes}. \end{array}$$ (24)

Our use of the spatial gradients of **X** when formulating these processes is discussed and motivated below. Note that none of these choices are “correct”. Rather, the four models were chosen in advance without any attempt to capture the true error $\tilde{X}-X$ in either space or time.

#### Results

**Fig 2:**
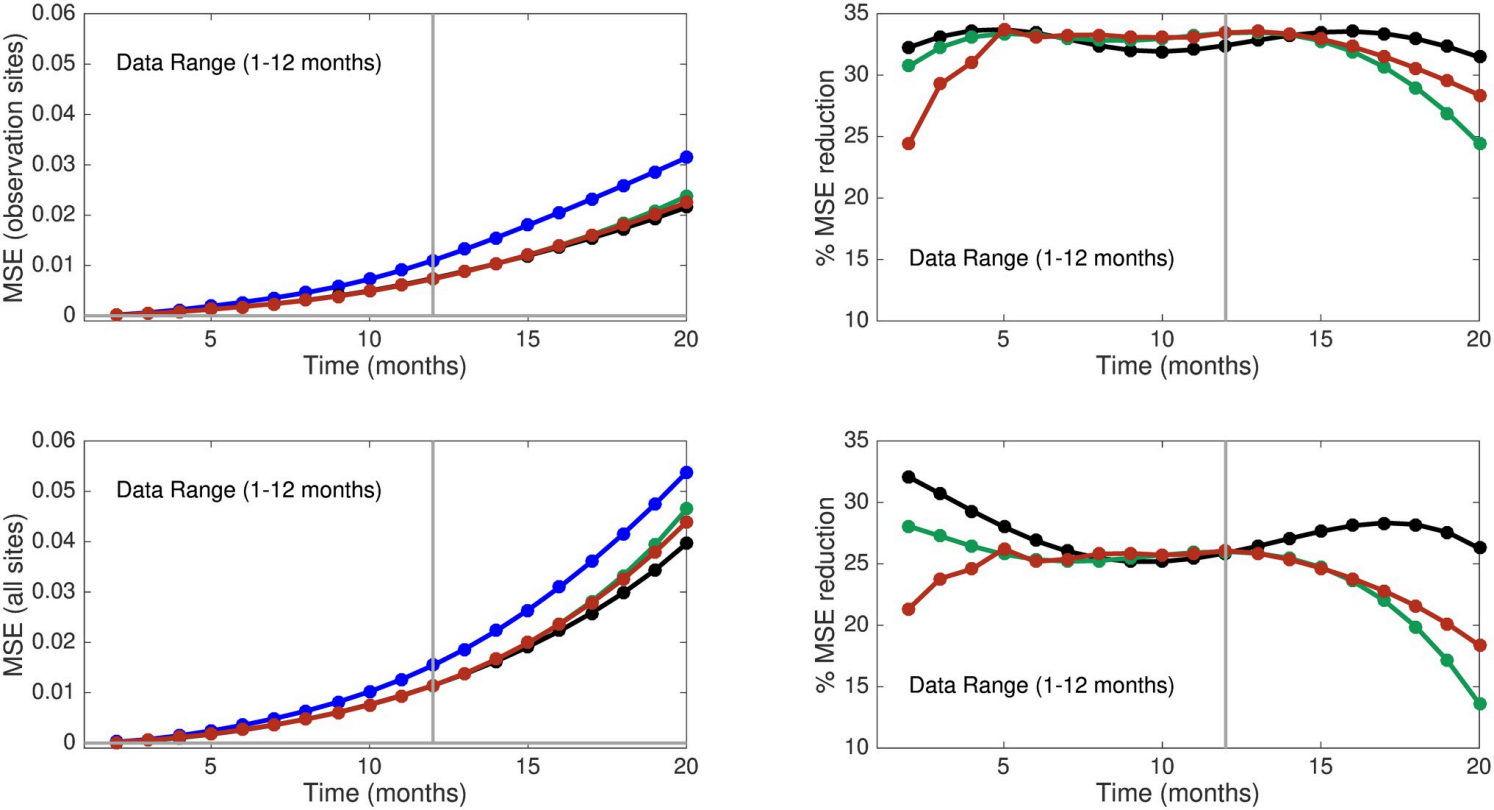
Predictive ability and percent reduction in MSE. Left: predictive ability: no model error (blue line), model (22)—black line, model (23)—green line, model (24)—red line. Right: percent reduction in MSE for the three models—versus “no model error”.

To assess the quality of estimates (i.e., *t* ≤ 12) and predictions (*t* > 12) of **X**, we consider spatially averaged mean squared errors

$$\begin{array}{c} MSE\left(t\right)=\frac{1}{\left|sites\right|}\;\sum_{\left(x,y\right)}\;{\left(\tilde{X}\left(x,y,t\right)-E\left(X\left(x,y,t\right)|Y\right)\right)}^{2} \end{array}$$ (25)

where *E*(**X**(*x*, *y*, *t*) | **Y**) is the posterior mean (or expectation) of the true process conditional on the observations **Y**. For all four error processes, we compute MSE in two cases: (i) average over the observation sites and (ii) average over all sites. The most obvious comment based on inspection of Fig 2 is that use of any of the three nontrivial models for ***η*** lead to better results than those obtained using assuming that ***η*** = 0 everywhere. This is suggestive of the Bayesian approach’s ability to use observational data effectively when using flexible, though even incorrect, models. The influence of the observations is evident when comparing achieved MSE at all sites with the MSE at observation sites only.

Recall that the true model involves spatially varying diffusion coefficients while the assumed model uses constant diffusion coefficients. This may explain why using error models that involve spatial partial derivatives of **X**(⋅, ⋅, *t*) is useful. This indicates that state-dependent error models are suggested in general. Model error prior models based on spatial gradients in **X** strikes us as mandated in the context of nonlinear physical models. Further, when model errors may be due to misspecifications of parameters, selection of error models based on the type of misspecifications deemed most likely may prove effective. In practice, we would not observe $\tilde{X}$ so calculation of the MSE as done here is not possible. However, some information is available by approximating MSE by replacing $\tilde{X}$ by **Y** at observation sites. Statistical model building ideas such as fitting the model using subsets of the observations and investigating how the models perform in predicting at the remaining sites may be valuable. We note that selection of error models may require substantial numerical computations of posterior distributions and comparisons of results. When feasible such model building approaches are recommended and potentially valuable in improving physical models.

### Class II approach

We again demonstrate approaches for the advection-diffusion Eq (17), but, for brevity, we only consider a spatial version. We assume the same 335 grid points as the observational network.

#### True process

A discretized version of the following model is used to produce the true process $\tilde{X}\left(x,y\right)$:

$$\begin{array}{c} \left\{ \begin{array}{c} \frac{\partial }{\partial x}(\kappa_{1}\frac{\partial \tilde{X}}{\partial x})+\frac{\partial }{\partial y}(\kappa_{2}\frac{\partial \tilde{X}}{\partial y})-u\frac{\partial \tilde{X}}{\partial x}-v\frac{\partial \tilde{X}}{\partial y}-\lambda \tilde{X}=0\;\;\left(x,y\right)\in \Omega \\ {\tilde{X}}_{\partial \Omega }=b \end{array}\right. \end{array}$$ (26)

where the velocities (*u*, *v*) are the same as in the *Class I Approach* and the diffusivities are specified as

$$\begin{array}{c} \kappa_{1}\left(x,y\right)=a_{1}x+a_{2}\;,\;\kappa_{2}\left(x,y\right)=b_{1}y+b_{2}. \end{array}$$

#### Observations

Analogous to G (recall the discussion around Eq (8)), we use an operator H to describe the sampling design used to obtain the observations. To produce an observational data vector **Y**^*o*^, we add simulated errors to functions of the true process $Y_{i}^{o}=H_{i}\left(\tilde{X}\right)+{∊}_{i},\;i=1,\ldots ,N$ where *i* indexes spatial locations and the *ϵ*_*i*_ are mutually independent Gaussian random variables with common mean 0 and unknown variance $\sigma_{y_{o}}^{2}$. This leads to an *N*-vector **Y**^*o*^:

$$\begin{array}{c} Y^{o}=\vec{H}\left(\tilde{X}\right)+∊ \end{array}$$ (27)

where $\vec{H}\left(\tilde{X}\right)$ is the appropriate vectorized form of $H(\tilde{X})$. For simplicity we assumed that the indices *i* of the observations $Y_{i}^{o}$ are associated with our grid points. This is not necessary and generalizations in which observations are not restricted to model grid points introduce notational but not conceptual differences.

#### Computer model output

To produce model output we used the model in Eq (26), but with constant coefficients *κ*_*x*_ and *κ*_*y*_, as our hypothesized computer model:

$$\begin{array}{c} \kappa_{x}\frac{\partial^{2}X}{\partial x^{2}}+\kappa_{y}\frac{\partial^{2}X}{\partial y^{2}}-u\frac{\partial X}{\partial x}-v\frac{\partial X}{\partial y}-\lambda X=0. \end{array}$$ (28)

A solution to this equation yields a computer model data vector **Y**^*c*^ that we define element-wise as $Y_{j}^{c}=\beta_{j}+G_{j}\left(X\right)+\gamma_{j};\;j=1,\ldots ,J$. Collecting the *β*_*j*_ into a *J*-vector ***β***, we assume the statistical model (recall Eq (8)),

$$\begin{array}{c} Y^{c}=\beta +\vec{G}\left(X\right)+\gamma , \end{array}$$ (29)

where **γ** is assumed to have a multivariate Gaussian distribution with mean 0 and covariance matrix **∑**_*y*^*c*^_.

#### Data model

For choices of G and H given below, we assume two conditionally independent data vectors with the following distributions:

$$\begin{array}{c} \left\{ \begin{array}{c} Y^{o}∼N\left(\vec{H}\left(X\right),\sum_{y^{o}}\right);\;\sum_{y^{o}}=\sigma_{y^{o}}^{2}\;I\;\text{and}\\ Y^{c}∼N\left(\beta +\vec{G}\left(X\right),\sum_{y^{c}}\right);\;\sum_{y^{c}}=\sigma_{y^{c}}^{2}\;I. \end{array}\right. \end{array}$$ (30)

In the simulations we set *σ*_*y*^*o*^_ = 0.23 and *σ*_*y*^*c*^_ = 0.1.

#### Process model priors

In all simulations we assume that **X** is a realization of a Gaussian Markov random field (GMRF). Markov random fields (MRF) are popular models in spatial statistics. They allow modelers to construct field models with specified local dependence structures. Specifically, at each site *s* the conditional probabilities for **X**(*s*) given the values of **X**(⋅) at *all* other sites actually depends only on values at a subset of neighboring sites. If all **X**(*s*) are assumed to be Gaussian random variables, the process is a GMRF. These models are characterized by the assumed prior mean field and covariance function which dictates variances at all sites and covariances at all pairs of sites, see [6, 29]. We also assigned GMRF priors for ***β*** except in two special cases below in which low dimensional prior models are used.

In all examples **X** and ***β*** are assumed to be a priori independent. They are expected to display dependence in the posterior distribution. The specific prior used for ***β*** in Examples 1 and 2 has the un-normalized density

$$\begin{array}{c} \text{exp}\{-\delta_{1}\sum_{s∼s^{\prime }}{\left(\beta_{s}-\beta_{s^{\prime }}\right)}^{2}-\delta_{1}^{\prime }\sum_{s}\beta_{s}^{2}\}\;, \end{array}$$ (31)

where *s* ∼ *s*′ denotes the north-south-east-west neighborhood structure. We set *δ*_1_ = 0.5 and $\delta_{1}^{\prime }=0.0001$ which corresponds to a relatively smooth, yet proper, distribution. The prior on **X** has un-normalized density

$$\begin{array}{c} \text{exp}\{-\delta_{2}\sum_{s∼s^{\prime }}{\left(X_{s}-X_{s^{\prime }}\right)}^{2}-\delta_{2}^{\prime }\sum_{s}X_{s}^{2}\}\;, \end{array}$$ (32)

where *s* ∼ *s*′ denotes the north-south-east-west neighborhood structure. We set *δ*_2_ = 0.1 and $\delta_{2}^{\prime }=0.0001$ which favors realizations that are not as smooth as realizations of ***β***. Finally, the operators G and H are defined as

$$\begin{array}{c} H_{s}\left(X\right)=G_{s}\left(X\right)=\frac{1}{9}(X_{s}+\sum_{s^{\prime }\approx s}X_{s^{\prime }}) \end{array}$$ (33)

where *s*′ ≈ *s* denotes the “eight nearest neighbors” *N*-*S*-*E*-*W*-*NE*-*NW*-*SE*-*SW* neighborhood structure. For brevity, we do not run examples involving averaging of observations. We select independent *IG*(4.0, 0.1) prior distributions for $\sigma_{y^{o}}^{2}$ and $\sigma_{y^{c}}^{2}$, where *IG*(*a*, *b*) stands for an Inverse Gamma distribution with probability density proportional to *x*^−*a*−1^exp(−*b*/*x*), *x* > 0.

#### Primary examples

We begin with two examples summarized coordinate-wise by

$$\begin{array}{cc} 1.\{\begin{array}{cc} Y_{i}^{o} & =X_{i}+\epsilon_{i},\\ Y_{j}^{c} & =\beta_{j}+X_{j}+\gamma_{j}, \end{array}\; & 2.\{\begin{array}{cc} Y_{i}^{o} & =X_{i}+\epsilon_{i},\\ Y_{j}^{c} & =\beta_{j}+G_{j}\left(X_{j}\right)+\gamma_{j}, \end{array} \end{array}$$ (34)

where *i* = 1, …, *N* and *j* = 1, …*J*.

#### Example 1

**Fig 3:**
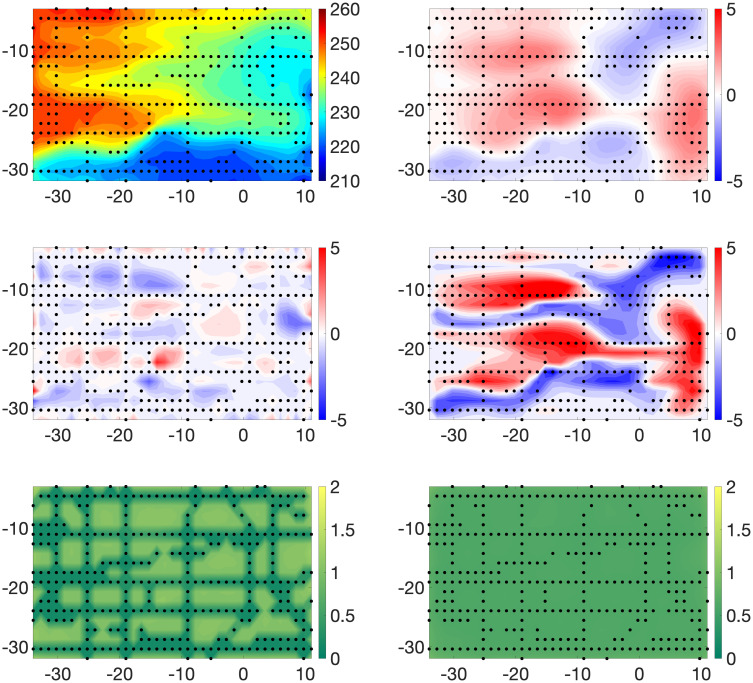
Example 1. Top Row: Posterior means of **X** (left panel) and ***β*** (right panel). Middle Row: True estimation errors: $\tilde{X}-E\left(X\;|\;Y^{0},Y^{c}\right)$ (left panel) and model biases: $\tilde{X}-Y^{c}$ (right panel). Bottom Row: Posterior standard deviations of **X** (left panel) and ***β*** (right panel).

**Fig 4:**
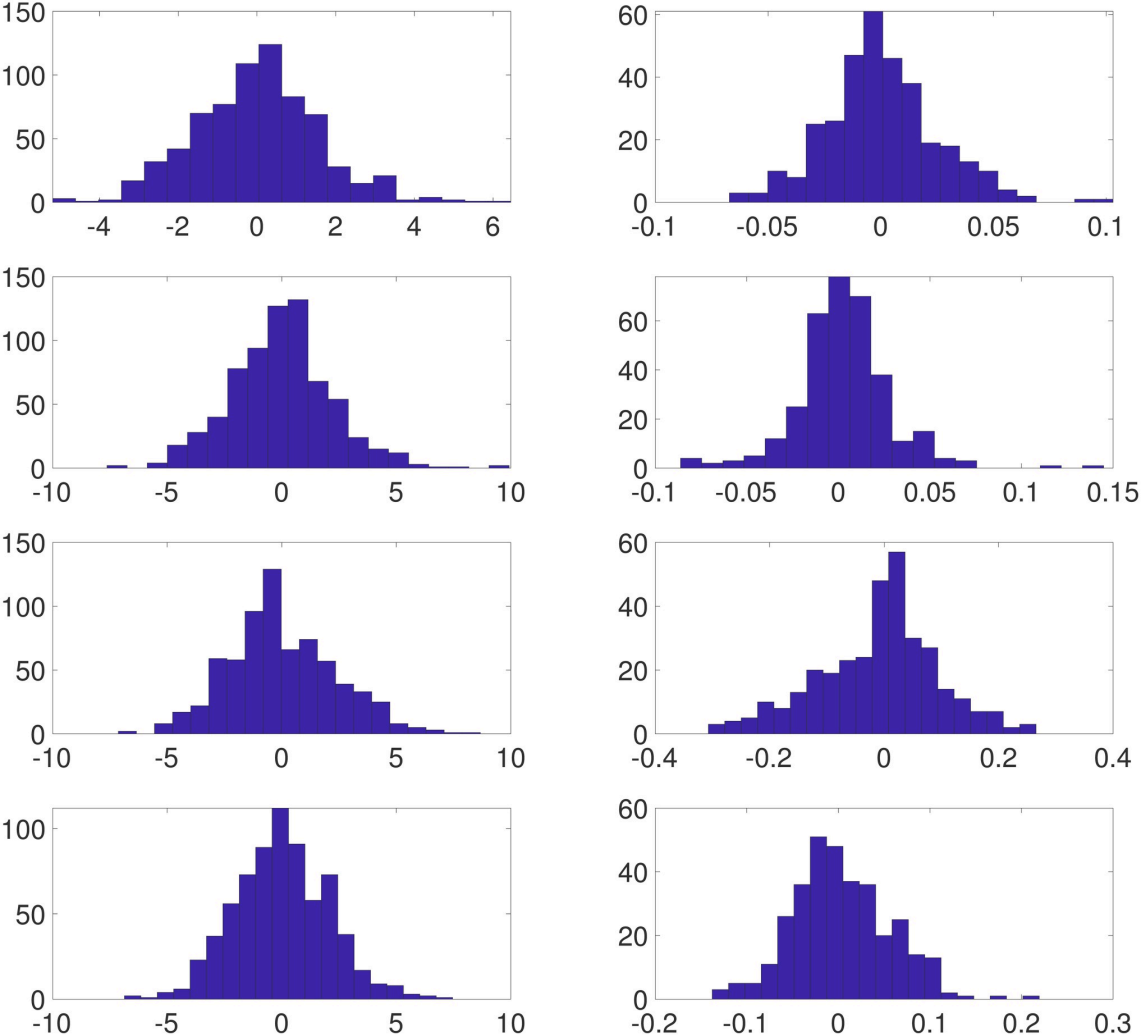
Histograms of residuals for Examples 1–4. Histograms of computer model residuals $Y^{c}-{\hat{Y}}^{c}$ (left side) and observation residuals $Y^{o}-{\hat{Y}}^{o}$ (right side) in rows 1–4, respectively. We note that the scale of the *x*-axis varies from plot to plot.

Fig 3 displays the posterior means and standard deviations of **X** and ***β*** for Example 1. The pattern of observational data sites stands out in the plot of the posterior standard deviations of **X**. As expected, these assessments of posterior uncertainty are dramatically smaller at observed sites than those at sites with no observational data. This feature is also indicated by the relatively small values at observation sites in the plot of the true estimation of errors. We note that the posterior standard deviations of ***β*** are much less variable spatially than those of **X**. This is reasonable since we “observed” the computer model at all grid points. The magnitudes of the posterior standard deviations of ***β*** are roughly concentrated in the middle of the range of those of **X**. The pattern and values of both the posterior means of the biases and the true biases in the western half of the region suggest that the computer model systematically underestimates the large values of the true $\tilde{X}$ and overestimates its small values. We also remark that this pattern of biases appears to be translated to the east by roughly 10°. Further, the posterior means of the biases capture the spatial pattern of the true biases very well. Histograms of residuals (i.e., $Y^{o}-{\hat{Y}}^{o},Y^{c}-{\hat{Y}}^{c}$) are shown in the top row of Fig 4. For Example 1, we define

$$\begin{array}{c} {\hat{Y}}^{o}=E\left(X\;|\;Y^{o}\right)\;{\hat{Y}}^{c}=E\left(\beta \;|\;Y^{o},Y^{c}\right)+E\left(X\;|\;Y^{o},Y^{c}\right) \end{array}$$

while for Example 2, ${\hat{Y}}^{c}=E\left(\beta \;|\;Y^{o},Y^{c}\right)+G\left(E\left(X\;|\;Y^{o},Y^{c}\right)\right)$. These graphics confirm and quantify the comparatively excellent estimation behavior at sites with physical observations.

#### Example 2

**Fig 5:**
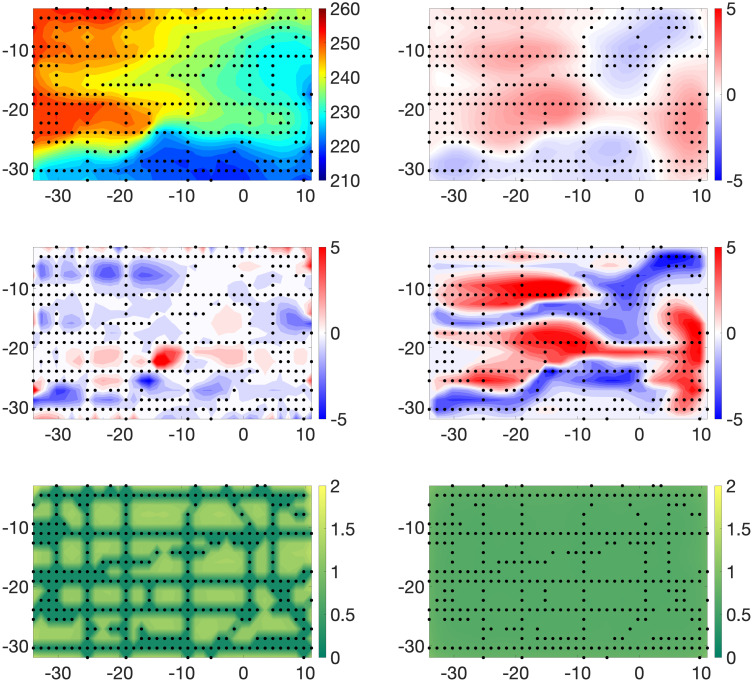
Example 2. Top Row: Posterior means of **X** (left panel) and ***β*** (right panel). Middle Row: True estimation errors: $\tilde{X}-E\left(X\;|\;Y^{o},Y^{c}\right)$ (left panel) and model biases: $\tilde{X}-Y^{c}$ (right panel). Bottom Row: Posterior standard deviations of **X** (left panel) and ***β*** (right panel).

Fig 5 displays the posterior means and standard deviations of **X** and ***β*** for Example 2. Recall that in this case we use local averages of computer model output in defining the means of model observations. The most obvious and expected impact of this procedure as opposed to that in Example 1 is a general degradation in the accuracy of estimates of the true process. Histograms of estimated residuals are again shown in Fig 4, second row. These graphics confirm and quantify the comparatively excellent estimation behavior at sites with physical observations. Further, when compared to their counterparts in Fig 4, they show the expected tendency of larger (in magnitude) residuals.

### Impacts of selected changes in the prior models

#### Low-dimensional priors for *β*

We maintain the assumptions used in Example 1 except for two alternative choices for the prior on ***β***.

#### Example 3

**Fig 6:**
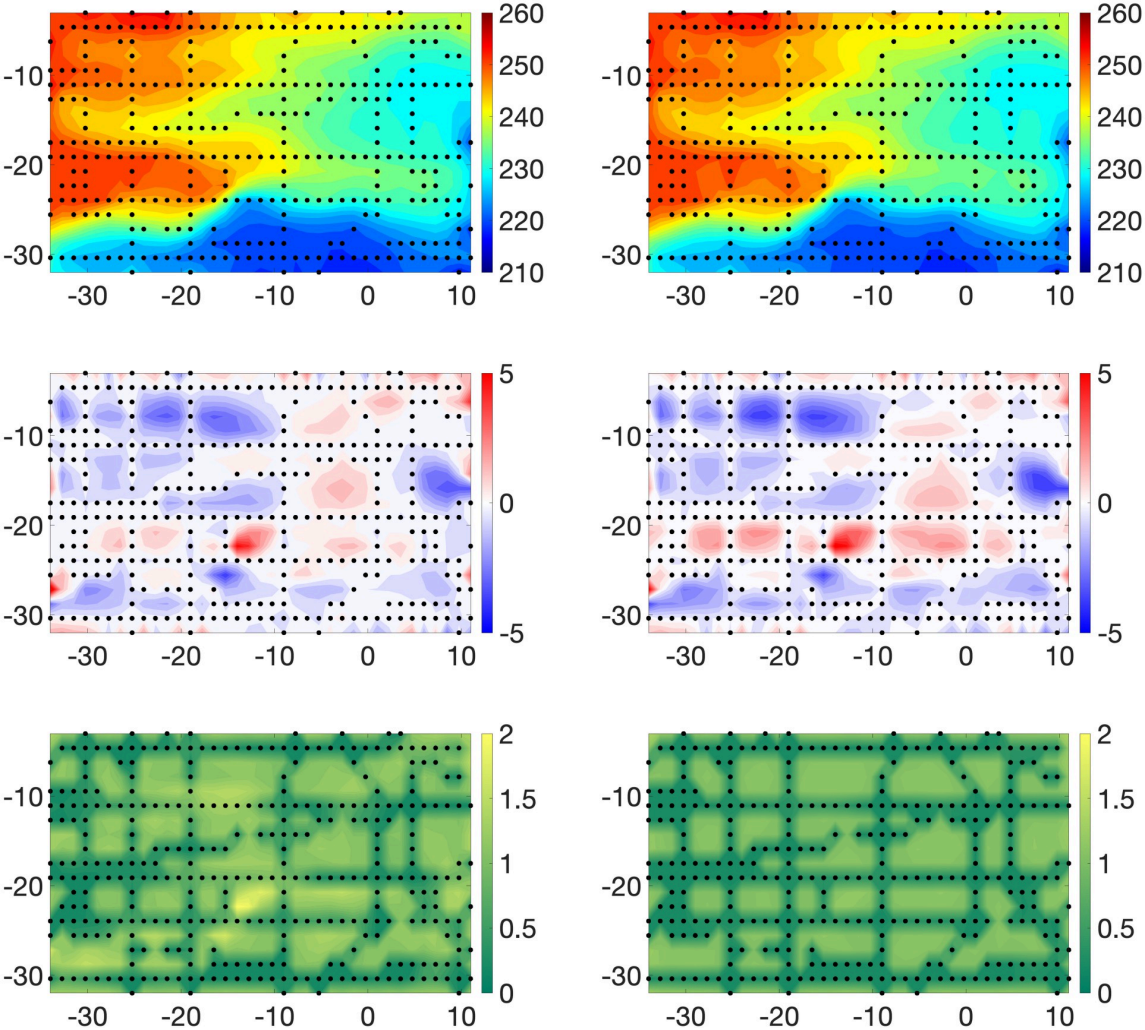
Examples 3–4. Constant-bias prior. The left panels correspond to a bias prior model which assumes that ***β*** is constant everywhere (Example 3). The right panels correspond to a bias prior model which assumes that ***β*** is row-wise constant (Example 4). Top: the posterior mean *E*(**X** | **Y**^*o*^, **Y**^*c*^). Middle: True estimation errors: $\tilde{X}-E\left(X\;|\;Y^{o},Y^{c}\right)$. Bottom: posterior standard deviations of **X**.

**Fig 7:**
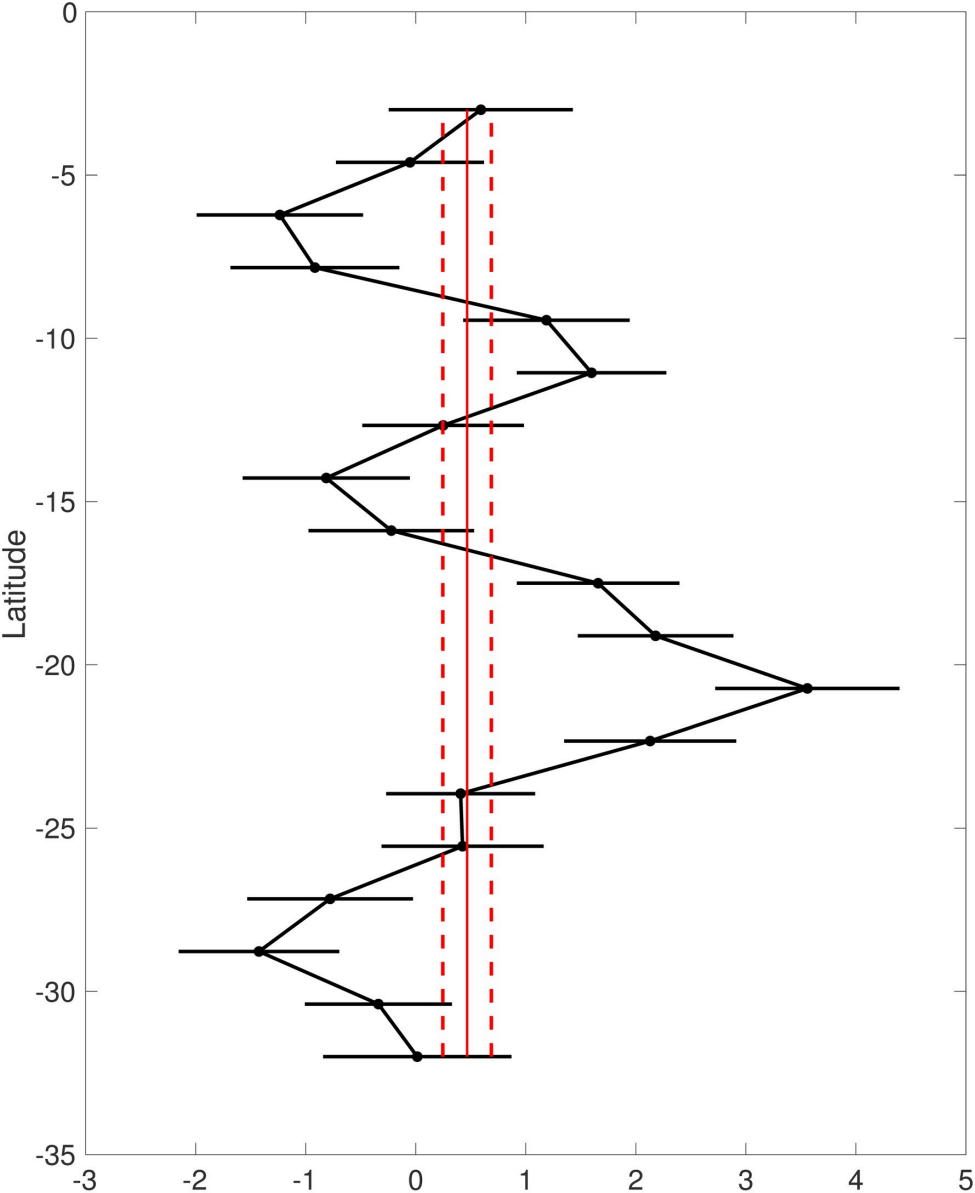
Examples 3–4. Posterior means and 90% credible intervals for biases in both the everywhere constant bias (red) and row-wise constant bias (black) examples.

We assume that the biases are equal at all grid points. Specifically, we assume that

$$\begin{array}{c} \beta =\beta_{0}1,\;\text{and}\;\beta_{0}∼N\left(0,\sigma_{\beta }^{2}\right)\;, \end{array}$$ (35)

where 1 is a vector of length 703 containing only 1’s. Again, we select an *IG*(4, 0.1) prior distribution for $\sigma_{\beta }^{2}$. Asummary of results is presented in Fig 6. As anticipated based on our earlier review of BK, this prior leads to relatively poor results, especially regarding the bias. As indicated in Fig 7, the posterior mean value 0.50 of *β*_0_ is very small compared to the range, roughly −5 to 5, of biases suggested in Fig 3. In combination with the very small posterior uncertainty regarding *β*_0_ as reflected by the narrowness of the 90% credible interval, the results suggest with high confidence that the model bias is small. This would likely lead to overconfidence in the computer model. Heuristically, the reason for this behavior is that there is a very large number of observations being used to form inference for a scalar.

#### Example 4

Next, we used a prior that assumes the biases are equal within each of the *R* = 19 rows of grid points. Such a prior assertion might arise in anticipation of highly zonal (east-west) behavior. Specifically, we set $\beta ={\left(\beta_{1}1^{\prime },\ldots ,\beta_{R}1^{\prime }\right)}^{\prime }$ where each 1 is a vector of all 1′*s* and of length equal to the number of columns (37) of grid points. The prior for the *R* horizontal biases used is a one-dimensional GMRF with un-normalized density given by

$$\begin{array}{c} \text{exp}\{-\delta_{3}\sum_{r=1}^{R-1}{\left(\beta_{r+1}-\beta_{r}\right)}^{2}-\delta_{3}^{\prime }\sum_{r=1}^{R}\beta_{r}^{2}\}\;, \end{array}$$ (36)

where we set *δ*_3_ = 0.5 and $\delta_{3}^{\prime }=0.0001$.

Fig 7 summarizes posterior inferences for the row-wise-constant biases and the everywhere-constant bias. The posterior standard deviations of **X** shown in Fig 7 coupled with the histograms of the residuals shown in Fig 4 again indicate poor behavior relative to that in Example 1 though somewhat better than the behavior in Example 3.

**Fig 8:**
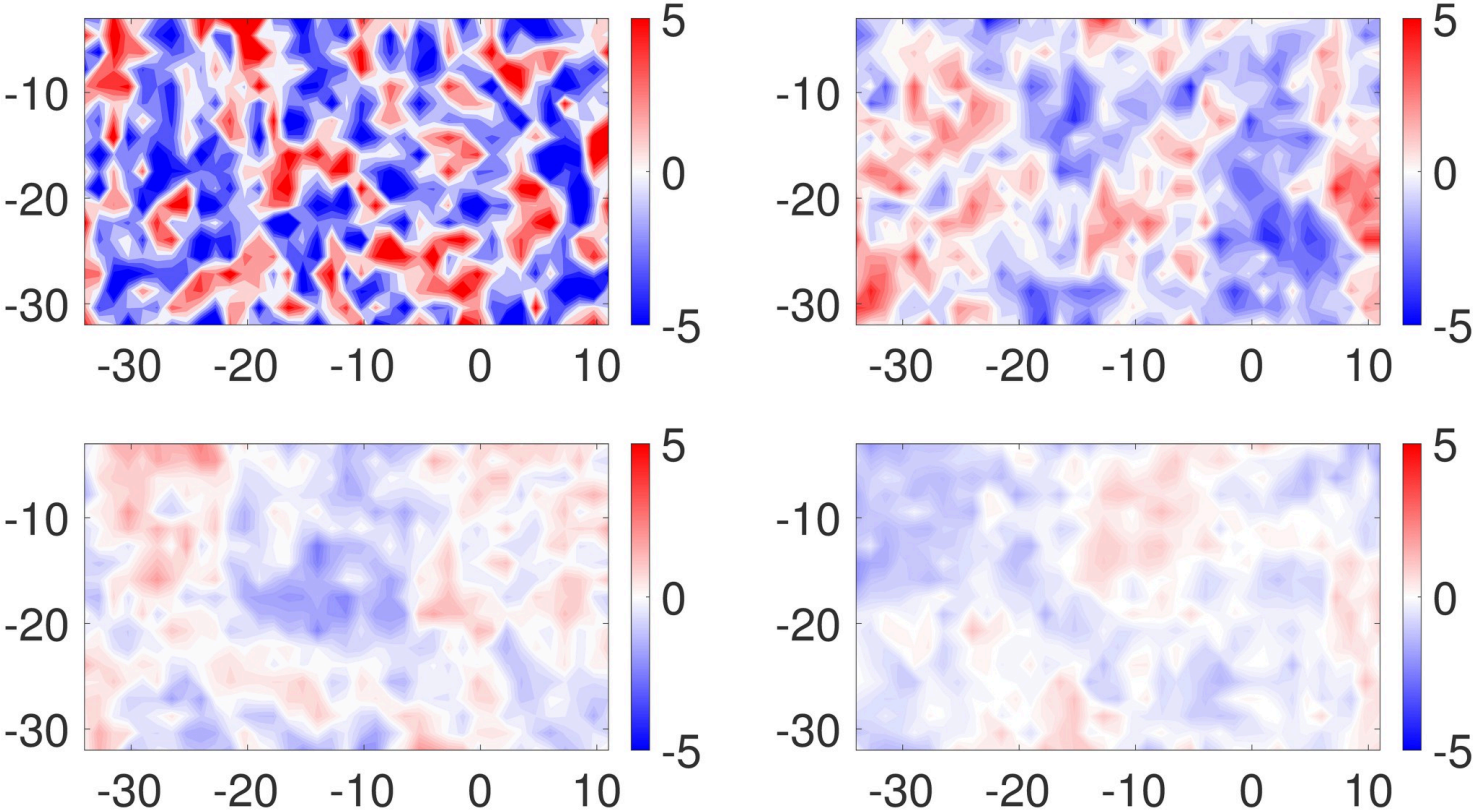
Draws from different prior distributions on β. Simulated random fields from the prior distribution on ***β*** with parameters *δ*_1_ = 0.01 (top-left), 0.1 (top-right), 0.5 (bottom-left) and 1.0 (bottom-right).

**Fig 9:**
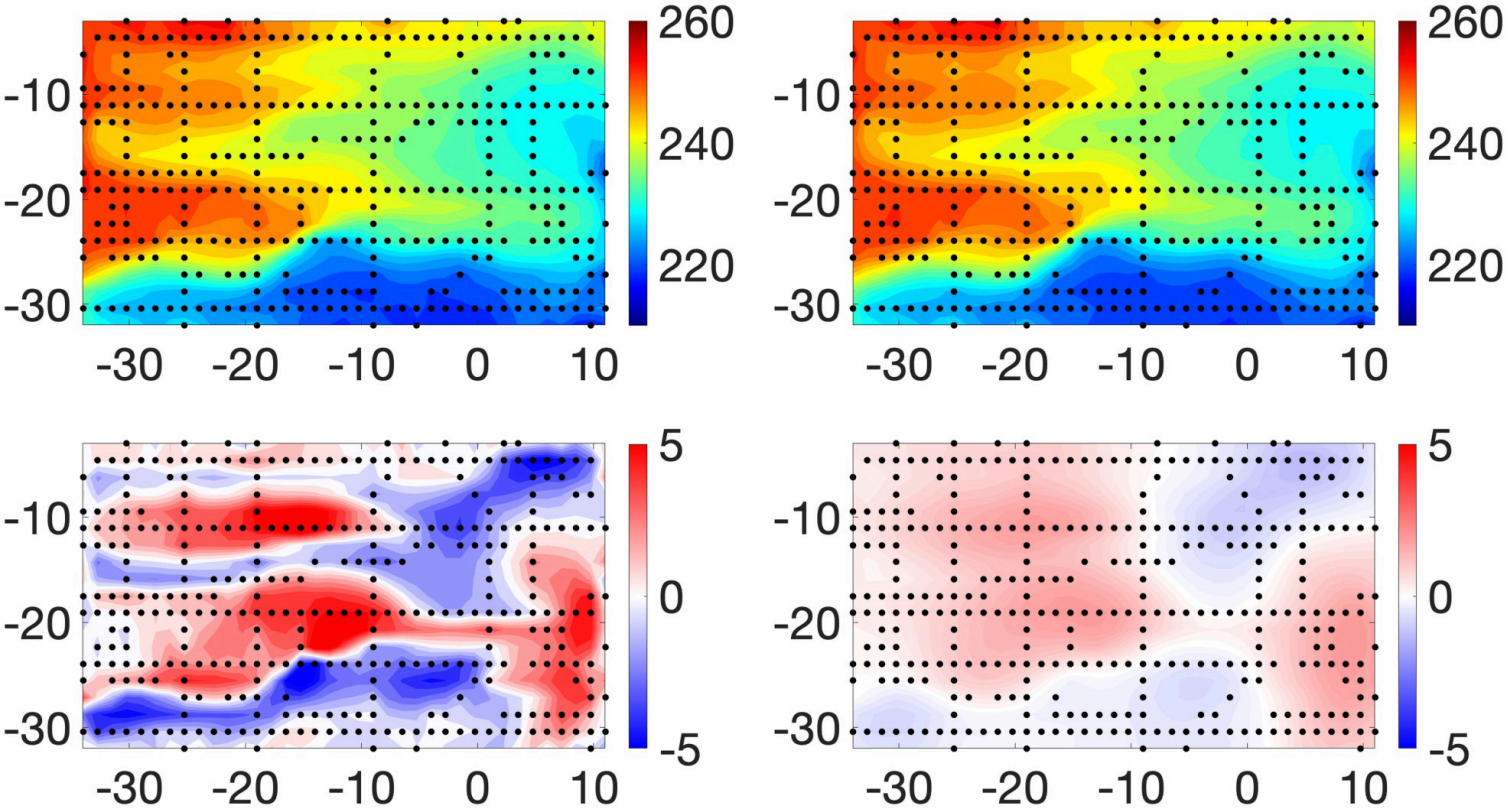
Results with different priors on β. Posterior means of **X** and ***β*** where *δ*_1_ = 0.1 (roughest, left panels) and *δ*_1_ = 1.0 (smoothest, right panels).

**Fig 10:**
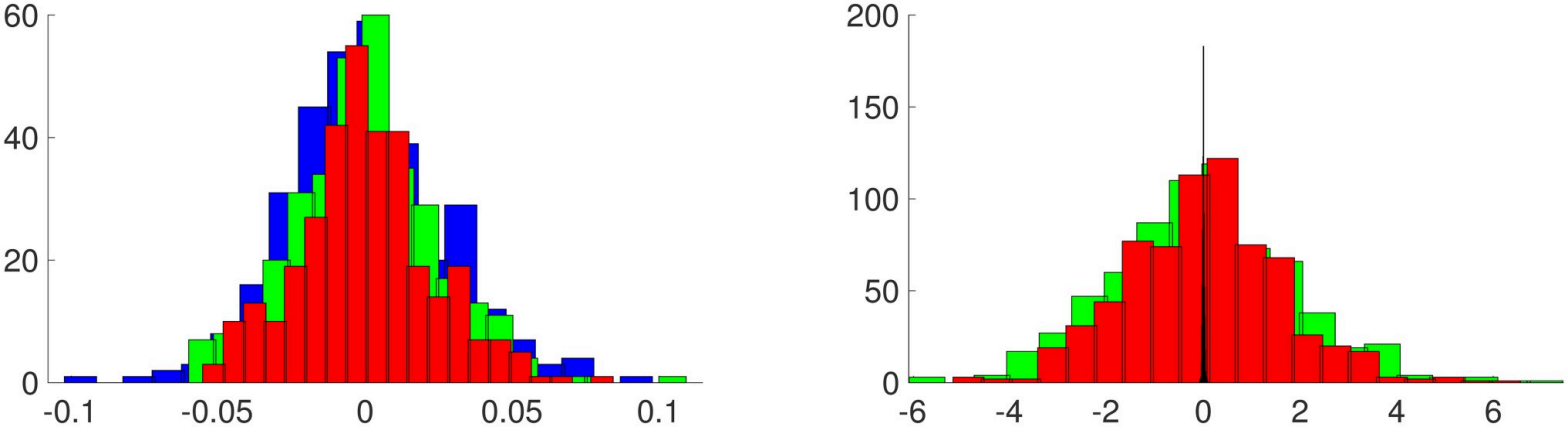
Histograms of residuals. The observation residuals $Y^{o}-{\hat{Y}}^{o}$ (left) and computer model residuals $Y^{c}-{\hat{Y}}^{c}$ (right) for the three choices of prior models: *δ*_1_ = 0.1 (blue), *δ*_1_ = 0.5 (red), *δ*_1_ = 1.0 (green).

**Varying GRMF priors on** ***β***: Recall the GMRF prior for ***β*** given in Eq (31). The parameter *δ*_1_ controls the smoothness of realizations of the model. As *δ*_1_ grows, the degree of local spatial correlation grows, leading to the appearance of increasing smoothness. To assess the degree of smoothness, Fig 8 shows realizations of ***β*** generated from a GMRF with *δ*_1_ set to *δ*_1_ = 0.01, 0.1, 0.5, 1.0. These draws do not necessarily look “like” the posterior mean of ***β*** we estimate, since this posterior mean is ultimately determined by the model and the data, as desired. The role of these draws is to show how much smoothness is imposed by the prior versus by data/statistical model. As we see, the data play the main role in determining the bias. Throughout the examples above, for the prior distribution on ***β*** we set *δ*_1_ = 0.5. We performed additional simulations using the setup of Example 1, but with *δ*_1_ = 0.1 and 1.0. For comparison with Fig 3, in Fig 9 we display posterior means for the process **X** and biases ***β*** where we used *δ*_1_ = 0.1 (left panels) and *δ*_1_ = 1.0 (right panels). Naturally, the choice of the prior for ***β*** has an effect on the ensuing inference, as observed in the posterior means for the two cases. When *δ*_1_ = 0.5 and 1.0, the posterior means *E*(***β*** | **Y**^*o*^, **Y**^*c*^) appear much smoother than typical draws from the corresponding priors (see Fig 8), indicating that the data play a crucial role. In the left panel of Fig 10, we overlay histograms of residuals $Y^{o}-{\hat{Y}}^{o}$ for the three cases *δ*_1_ = 0.1 (blue), *δ*_1_ = 0.5 (red) and *δ*_1_ = 1.0 (green). Visually, it appears that the two additional priors (*δ*_1_ = 0.1, *δ*_1_ = 1.0) yield slightly larger residuals for observations. The message is different when analyzing the computer model residuals. When *δ*_1_ = 0.1, the prior model for ***β*** “allows” for high gradients as can be seen in the bottom left panel of Fig 9. As such, the dangers of using uninformative (non-regularized) priors are evidenced again in the right panel of Fig 10. When setting *δ*_1_ = 0.1, the computer model residuals $Y^{c}-{\hat{Y}}^{c}$ are very small (narrow, blue histogram in the right panel of Fig 10). In this case, by exhibiting high gradinents, the estimated bias *E*(***β*** | **Y**^*o*^, **Y**^*c*^) compensates for nearly *all* of the discrepancy between **Y**^*c*^ and **X**.

## Conclusion

### Related models

In Sections 1.1 and 1.3, we contrasted numerical model error with apparent model errors such as those resulting from errors in predictor variables. Specifically, on the right hand side of Eq (2), ∂**X**/∂*t* = **h**(**X**, **Z**, ***θ***_*x*_, ***θ***_*z*_), we included **Z** to represent relevant processes and ***θ***_*z*_ are additional parameters that arise in the modeling.

Approaches to predictive problems depend on whether or not the values of the predictor variables are known or unknown. If the predictors are known, say **Z** = **z**, we can define the process model prior as [**X**∣**z**, ***θ***_*x*_, ***θ***_*z*_]. This view is very common. In some cases, observations or other estimates of **Z** are used to produce **z**. For example, [10] analyzed use of a hydrological model to predict ground-water flow (**X**). One of several inputs (**z**) they used was “a time series of measured rainfall data”.

If **Z** are modeled as unknown, our analysis depends on the sources of information regarding **Z**. As in the case of **X**, one source are observational data, say **Y**^*z*^, with data model $\left[Y^{z}|Z,\theta_{y_{z}}\right]$. Another source is a physical model, which we suppose is of the form assumed in Eq (2) and leads to a physical-statistical model (analogous to Eq (3))

$$\begin{array}{c} q\left(Z,\zeta ,\theta_{z}\right)\left(s,t\right))=0\;,\;\;\left(s,t\right)\in D\subset \tilde{D}\;, \end{array}$$ (37)

where **ζ** is a stochastic process used to represent error in the specification of **q**, and ***θ***_*z*_ is an additional unknown multivariate parameter. These issues are beyond our scope here.

Prediction of a dynamical system based on a time series of observations is another relevant setting. The example in Section 3.1 is such a case. Suppose we can break up a discrete space-time field **X** to form a time series of spatial vectors **x**_1_, …, **x**_*T*_. Model error may be modeled as a time series ***η***_1_, …, ***η***_*T*_. We typically incorporate observational data vectors **y**_1_, …, **y**_*T*_ sequentially. The details are much like those associated with the familiar *Kalman filtering* procedure, so we do not present them here.

### Discussion

Our priority here is the development of statistical prediction models that combine data and physically-based, mechanistic models and account for model error. We considered two classes of mechanistic models. In the first, we incorporated a model error process into a physical model. The resulting posterior probability distribution for model error

- improves predictions and enables quantification of associated uncertainties;
- may provide opportunities to improve physical models and perhaps lead to new science.

We also discussed the issue of selection of priors for model error and provided some illustrations.

The second class arises when models are deemed to be too large and complex for the Class I approach to be practical. Our suggestion revolves around dimension reduction. We consider low-dimensional summaries or functions of the primary processes of interest. The corresponding summaries of computer model output are modeled as *biased observations* of the true dimension-reduced processes. Combining such computer model “observations” and observational data is conceptually simple. We also indicated how this approach is very useful for combining output from multiple models.

An important issue is how to perform statistical inference for such complex models. The illustrations presented in this manuscript are fitted via a traditional Metropolis-Hastings algorithm. When necessary, classical Bayesian inference methods can be combined with more modern samplers, such as Hamiltonian Monte Carlo (see [30] for a recent overview), or Approximate Bayesian Computation [31, 32].

We believe that the use of low-dimensional summaries of large-scale computer model output is necessary and hence pervasive. For example, climate system models produce state vectors of dimensions in 100’s of millions. Such massive model output becomes useful when dimension reduced variables are studied. Further, summaries such as a time series of model estimates of global average temperatures are useful when explaining climate change to policy makers and the public.

We view the biases described here as unknown, inaccessible functions of model errors present in large-scale model output. We discussed some of the difficulties in formulating prior distributions for these biases. Adding to the difficulties, the functions leading to biases may be state- and/or parameter-dependent. Gaussian processes and hierarchical (deep) Gaussian processes are a very flexible class of models, capable of capturing a wide range of behaviors, and thus they are the go-to choice as statistical models for these unknowns. Nevertheless, The importance and prevalence of dimension-reduced variables derived from model output indicates that our suggestions merit further research.

## Supporting information

S1 Data The results in this manuscript are based on simulated data. The file suporting_information.zip contains the MATLAB code and the synthetic data that was used to produce all the figures in this manuscript.
(ZIP)
