# Supplementary material for: Excursions in the Bayesian treatment of model error
Source: PLoS One. 2023 Jun 2;18(6):e0286624. doi: 10.1371/journal.pone.0286624 (PMC10237458; doi:10.1371/journal.pone.0286624)
Supplement: S1 Data — The file suporting_information.zip contains the MATLAB code and the synthetic data that was used to produce all the figures in this manuscript. (ZIP) [file pone.0286624.s001.zip › Figure1/m_map/map.html]

M\_Map: A Mapping package for Matlab


- Introduction
- Gallery
- Getting M\_Map
- Release Notes
- Users Guide
- Example Code
- Citation
- Acknowledgements

Last changed 20/Apr/2021. Questions and comments to rich@eos.ubc.ca

# M\_Map:

## A mapping package for Matlab

---

You have collected your data, loaded it into Matlab, analyzed everything to
death, and now you want to make a simple map showing how it relates to the world.

But you can't.

Instead you have to figure out how to save all your data, and then
read it into another program (like, for example GMT), and then spend all that
extra time figuring out why it doesn't give you what you expected it
would...or you can invest in Matlab's own mapping toolbox (with a similarly steep learning curve)... or not!

## Announcing M\_Map v1.4n! (released Sept/2021)

M\_Map is a set of mapping tools written for Matlab (it also works under Octave). M\_Map includes:

1. Routines to project data in 20 different projections
   (and determine inverse mappings), using spherical and ellipsoidal earth-models.
2. A grid generation routine to make nice axes with limits either
   in lat/long terms or in planar X/Y terms.
3. A coastline database (with 1/4 degree resolution).
4. A global elevation database (1 degree resolution).
5. Hooks into freely available high-resolution coastline and
   bathymetry databases.
6. Other useful stuff.

---

## Gallery

|  |  |  |  |  |
| --- | --- | --- | --- | --- |
|  |  |  |  |  |
|  |  |  |  |  |
|  |  |  |  |  |
|  |  |  |  |  |
| |  |  |  | |
|  |  |  |  |  |

  
  


---

## How to get M\_Map

You can download the M\_Map toolbox either as a gzipped tar-file ,
or as zip archive
(Click on these links to download - note, some problems with this have been reported by users of the Chrome
web browser). If you are unpacking the zip
file MAKE SURE YOU
ALSO UNPACK SUBDIRECTORIES! Both are around 650k in size. Once you have
this
archive, read the Getting started section
of the User's guide to correctly
install this toolbox, and sections 8.6
and 9.3 to install ETOPO1
and GSHHS respectively.

A number of examples are available to
highlight the various capabilities of M\_Map (thumbnails are shown
above).

M\_Map is a programming toolbox. However, you can also explore
mapping using MAP-LAB,
a a MATLAB-based Graphical User Interface (GUI) designed to produce maps and visualize data sets
related to geodetic, geophysical and oceanographic applications, which is built on top of M\_Map.
MAP\_Lab is available here.


---

## Release Notes

New in release 1.4n (Sep/2021):

1. Created a separate `/data` directory for coastline databases (instead of defaulting them into
   `/private`).
2. Updated OCTAVE compatability

New in release 1.4m (Feb/2020):

1. Added cylindrical equal-area projection for completeness (don't recommend using it though!)
2. Added `m_ginput` to assist in mouse-driven point selection.
3. Added date argument to `m_coord` so you can use the 13th IGRF model for geomagnetic coordinates at any date between 1900 and 1925.
4. Fixed bugs in `m_mag2geo` and `m_geo2mag` that made them work incorrectly in some circumstances, and added
   documentation about their use to the users guide.
5. Added `m_northarrow` to show the direction of north.
6. Added `m_windrose` to show wind rose diagrams on the map.

New in release 1.4k (Mar/2019):

1. Finally - a way to handle pixellated data with `m_image`!
2. Checked in Octave up to v4.4.1

New in release 1.4j (May/2018):

1. Shaded relief mapping with `m_shadedrelief`.
2. An independent grid for UTM maps with `m_utmgrid`

New in release 1.4i (Jan/2018):

1. Finally sorted out the major "2014b and later matlab graphics engine" related problems with `m_grid`, as well as many
   minor graphics improvements.- `m_windbarb` and `m_streamline` for meteorologists, also `m_scatter`.- A set of useful colourmaps in `m_colmap`, including a perceptually uniform "jet-like" colourmap.- A colourbar function `m_contfbar` made to work with `contourf` plots.- `m_annotation` and `wysiwyg` to help you add arrows and things.
         - Updated the documentation so it doesn't have that "1990s" look.
         - Added link to a Chinese translation of the Users Guide, as well as to the mapping GUI MAP-LAB which is built on top of M\_Map.
         - List of coded examples updated, and a few new ones added.

New in release 1.4h (Nov/2017) are

1. ETOPO2v2 and ETOPO1 support.- Handled some warnings about BITMAX that arise in Matlab 2014b. Note - I have only been able to
     fix or workaround SOME of the really bad bugs in 2014b. Use with caution (or better yet don't use). EPS
     output in particular is really awful, even if the screen plot looks "nice". Fortunately mathworks
     "may consider fixing this in future releases".- Added option to output national+state or national borders only in `m_gshhs`.

New in release 1.4g (Jan/2014) are

1. `m_ellipse.m` to plot tidal ellipses (say, from  T\_Tide )- Option to use either decimal-degrees or degrees/decimal-minutes in grid axis labels- Matlab 2013b has a bad bug in the `dataaspectratio` axes property that affects pretty much everything graphical. This version of
       M\_Map has a workaround that MOSTLY works, but it would
       be better to avoid using 2013b.

New in release 1.4f (May/2012) are

1. `m_shaperead.m` to read SHAPEFILES! (so Natural Earth can be
   used for political boundaries instead of DCW, among other opportunities)
2. gshhs version 2 support
3. partial OCTAVE compatibility

New in release 1.4e (May/2010)

1. GSHHS Now comes with the WDB rivers and borders database, and
   links to them have been added via `m_gshhs.m`
2. Ellipsoidal Albers and Lambert conic projections included

New in release 1.4d (Oct/2007) are

1. Modifications to work around bugs in matlab7 `contourf`
2. Robinson projection
3. A few compatibility issues with current matlab versions- Upgraded hooks into some databases.

New in release 1.4b (Jun/2006) are

1. m\_hatch for hatched and speckled patches
2. old-fashioned speckled coastlines (good for B&W pics - see Example 13).
3. m\_lldist now also returns points on great circle geodesics.
4. `m_fdist, m_idist`, and `m_geodesic` for geodesics on an ellipsoidal
   earth.

New in release 1.4 (Nov/2005) are

1. `m_pcolor`
2. `m_coord` (to allow for geographic and geomagnetic coordinate
   systems)
3. A very few minor bug fixes.
4. Some hints about and examples of adding satellite image data to
   your maps.

---

## Example Code

#### 1. M\_Map Logo

```
m_proj('ortho','lat',48','long',-123');
m_coast('patch','r');
m_grid('linest','-','xticklabels',[],'yticklabels',[]);

patch(.55*[-1 1 1 -1],.25*[-1 -1 1 1]-.55,'w'); 
text(0,-.55,'M\_Map','fontsize',25,'color','b',...
    'verticalalignment','middle','horizontalalignment','center');
```

#### 2. Lambert Conformal Conic projection of North American Topography

```
m_proj('lambert','long',[-160 -40],'lat',[30 80]);
m_coast('patch',[1 .85 .7]);
m_elev('contourf',[500:500:6000]);
m_grid('box','fancy','tickdir','in');
colormap(flipud(copper));
```

#### 3. Stereographic projection of North Polar regions

```
% Note that coastline is drawn OVER the grid because of the order in which
% the two routines are called

m_proj('stereographic','lat',90,'long',30,'radius',25);
m_elev('contour',[-3500:1000:-500],'edgecolor','b');
m_grid('xtick',12,'tickdir','out','ytick',[70 80],'linest','-');
m_coast('patch',[.7 .7 .7],'edgecolor','r');
```

#### 4. Two Interrupted Projections of the World's Oceans

```
subplot(211);
Slongs=[-100 0;-75 25;0 45; 25 145;45 100;145 295;100 295];
Slats= [   8 80;-80  8;  8  80;-80   8; 8   80;-80   0; 0 80];
for l=1:7
    m_proj('sinusoidal','long',Slongs(l,:),'lat',Slats(l,:));
    m_grid('fontsize',6,'xticklabels',[],'xtick',[-180:30:360],...
                'ytick',[-80:20:80],'yticklabels',[],'linest','-','color',[.7 .7 .7]);
    m_coast('patch','g');
end
xlabel('Interrupted Sinusoidal Projection of World Oceans');

% In order to see all the maps we must undo the axis limits set by m_grid calls:
set(gca,'xlimmode','auto','ylimmode','auto');


subplot(212);
Slongs=[-100 43;-75 20; 20 145;43 100;145 295;100 295];
Slats= [  0  90;-90  0;-90   0; 0  90;-90   0;  0  90];
for l=1:6
    m_proj('mollweide','long',Slongs(l,:),'lat',Slats(l,:));
    m_grid('fontsize',6,'xticklabels',[],'xtick',[-180:30:360],...
                'ytick',[-80:20:80],'yticklabels',[],'linest','-','color','k')
    m_coast('patch',[.6 .6 .6]);
end
xlabel('Interrupted Mollweide Projection of World Oceans');

set(gca,'xlimmode','auto','ylimmode','auto');
```

#### 5. Oblique Mercator Projection with quiver and contour data

```
%% Nice looking data
[lon,lat]=meshgrid([-136:2:-114],[36:2:54]);
u=sin(lat/6);  
v=sin(lon/6);

m_proj('oblique','lat',[56 30],'lon',[-132 -120],'aspect',.8);
subplot(121);
m_coast('patch',[.9 .9 .9],'edgecolor','none');
m_grid('tickdir','out','yaxislocation','right',...
            'xaxislocation','top','xlabeldir','end','ticklen',.02);
hold on;
m_quiver(lon,lat,u,v);
xlabel('Simulated surface winds');
subplot(122);
m_coast('patch',[.9 .9 .9],'edgecolor','none');  
m_grid('tickdir','out','yticklabels',[],...
              'xticklabels',[],'linestyle','none','ticklen',.02);
hold on;
[cs,h]=m_contour(lon,lat,sqrt(u.*u+v.*v));
clabel(cs,h,'fontsize',8);
xlabel('Simulated something else');
```

#### 6. Miller Projection with Great Circle

```
% Plot a circular orbit
lon=[-180:180];
lat=atan(tan(60*pi/180)*cos((lon-30)*pi/180))*180/pi;
m_proj('miller','lat',82);
m_coast('color',[0 .6 0]);
m_line(lon,lat,'linewi',3,'color','r');
m_grid('linestyle','none','box','fancy','tickdir','out');
m_northarrow(-150,0,40,'type',4,'linewi',.5);
```

#### 7. Lambert Conformal Projection with high-resolution bathymetry of Western Mediterranean

```
m_proj('lambert','lon',[-10 20],'lat',[33 48]); 

[CS,CH]=m_etopo2('contourf',[-5000:500:0 250:250:3000],'edgecolor','none');
 m_grid('linestyle','none','tickdir','out','linewidth',3);

colormap([ m_colmap('blues',80); m_colmap('gland',48)]);
brighten(.5);

ax=m_contfbar(1,[.5 .8],CS,CH);
title(ax,{'Level/m',''}); % Move up by inserting a blank line
```

#### 8. Demonstration of fancy vectors

```
m_vec; % See code in m_vec.m for details
```

#### 9. Zoom in on Prince Edward Island to show different coastline resolutions

```
% Example showing the default coastline and all of the different resolutions
% of GSHHS coastlines as we zoom in on a section of Prince Edward Island.

    clf
    axes('position',[.35 .6 .37 .37]);
    m_proj('albers equal-area','lat',[40 60],'long',[-90 -50],'rect','on');
    m_coast('patch',[0 1 0]);
    m_grid('linestyle','none','linewidth',2,'tickdir','out',...
           'xaxisloc','top','yaxisloc','right','fontsize',6);
    m_text(-69,51,'Standard coastline','color','r','fontweight','bold');
    m_ruler([.5 .9],.8,3,'fontsize',8)
    
    axes('position',[.09 .5 .37 .37]);
    m_proj('albers equal-area','lat',[40 54],'long',[-80 -55],'rect','on');
    m_gshhs_c('patch',[.2 .8 .2]);
    m_grid('linestyle','none','linewidth',2,'tickdir','out',...
           'xaxisloc','top','fontsize',6);
    m_text(-80,52.5,'GSHHS\_C (crude)','color','m','fontweight','bold');
    m_ruler([.5 .9],.8,2,'fontsize',8);
      
    axes('position',[.13 .2 .37 .37]);
    m_proj('albers equal-area','lat',[43 48],'long',[-67 -58],'rect','on');
    m_gshhs_l('patch',[.4 .6 .4]);
    m_grid('linestyle','none','linewidth',2,'tickdir','out','fontsize',6);
    m_text(-66.5,43.5,'GSHHS\_L (low)','color','m','fontweight','bold');
    m_ruler([.5 .9],.8,3,'fontsize',8);
     
    axes('position',[.35 .05 .37 .37]);
    m_proj('albers equal-area','lat',[45.8 47.2],'long',[-64.5 -62],'rect','on');
    m_gshhs_i('patch',[.5 .6 .5]);
    m_grid('linestyle','none','linewidth',2,'tickdir','out',...
           'yaxisloc','right','fontsize',6);
    m_text(-64.4,45.9,'GSHHS\_I (intermediate)   ','color','m',...
          'fontweight','bold','horizontalalignment','right');
    m_ruler([.5 .8],.1,3,'fontsize',8);
     
    axes('position',[.5 .1 .37 .37]);
    m_proj('albers equal-area','lat',[46.375 46.6],'long',[-64.2 -63.7],'rect','on');
    m_gshhs_h('patch',[.6 .7 .6]);
    m_grid('linestyle','none','linewidth',2,'tickdir','out',...
           'xaxisloc','top','yaxisloc','right','fontsize',6);
    m_text(-64.18,46.4,'GSHHS\_H (high)','color','m','fontweight','bold');
    m_ruler([.5 .8],.2,3,'fontsize',8);
     
    axes('position',[.55 .35 .37 .37]);
    m_proj('albers equal-area','lat',[46.55 46.65],'long',[-63.97 -63.77],'rect','on');
    m_gshhs_f('patch',[.7 .9 .7]);
    m_grid('linestyle','none','linewidth',2,'tickdir','out',...
           'xaxisloc','top','yaxisloc','right','fontsize',6);
    m_text(-63.95,46.56,'GSHHS\_F (full)','color','m','fontweight','bold');
    m_ruler([.5 .8],.2,3,'fontsize',8);
```

#### 10. Tracklines and UTM projection

```
    m_proj('UTM','long',[-72 -68],'lat',[40 44]);
    m_gshhs_i('color','k');
    m_grid('box','fancy','tickdir','in');
    m_ruler(1.2,[.5 .8]);
    
    % fake up a trackline
    lons=[-71:.1:-67];
    lats=60*cos((lons+115)*pi/180);
    dates=datenum(1997,10,23,15,1:41,zeros(1,41));

    m_track(lons,lats,dates,'ticks',0,'times',4,'dates',8,...
           'clip','off','color','r','orient','upright');
    
    m_northarrow(-68.5,43.4,.4,'type',2);
```

#### 11. Range rings

```
m_proj('hammer','clong',170);
m_grid('xtick',[],'ytick',[],'linestyle','-');
m_coast('patch','g');
m_line(100.5,13.5,'marker','square','color','r');
m_range_ring(100.5,13.5,[1000:1000:15000],'color','b','linewi',2);
xlabel('1000km range rings from Bangkok');
```

#### 12. Speckled boundary

```
    bndry_lon=[-128.8 -128.8 -128.3 -128 -126.8 -126.6 -128.8];
    bndry_lat=[49      50.33  50.33  50   49.5   49     49];
    
    clf;
    m_proj('lambert','long',[-130 -121.5],'lat',[47 51.5],'rectbox','on');

    m_gshhs_i('color','k');              % Coastline...
    m_gshhs_i('speckle','color','k');    % with speckle added

    m_line(bndry_lon,bndry_lat,'linewi',2,'color','k');     % Area outline ...
    m_hatch(bndry_lon,bndry_lat,'single',30,5,'color','k'); % ...with hatching added.

    m_grid('linewi',2,'linest','none','tickdir','out','fontsize',12);
    title({'Speckled Boundaries','for nice B&W presentation','(best in postscript format)'});
    m_text(-128,48,{'Pacific','Ocean'},'fontsize',18);
    
    m_northarrow(-122.5,50.2,.8,'type',3,'linewi',2);
```

#### 13. Blue Ocean

```
    m_proj('miller','lat',[-77 77]);   
    m_coast('patch',[.7 1 .7],'edgecolor','none'); 
    m_grid('box','fancy','linestyle','-','gridcolor','w','backcolor',[.2 .65 1]);
    
    cities={'Cairo','Washington','Buenos Aires'}; 
    lons=[ 30+2/60  -77-2/60   -58-22/60];
    lats=[ 31+21/60  38+53/60  -34-45/60]; 
    for k=1:3
      [range,ln,lt]=m_lldist([-123-6/60 lons(k)],[49+13/60  lats(k)],40); 
      m_line(ln,lt,'color','r','linewi',2); 
      m_text(ln(end),lt(end),sprintf('%s - %d km',cities{k},round(range)));
    end;
    title('Great Circle Routes','fontsize',14,'fontweight','bold');
    
    set(gcf,'color','w');   % Need to do this otherwise 'print' turns the lakes black
```

#### 14. One Ocean Projection

```
    %  This projection shows all the oceans connected to each other - the outside ring
    %  is the Asian coastline (Thanks to M B-O for this idea)
    % otherwise its just an example of different map types.
    
     m_proj('azimuthal equal-area','radius',156,'lat',-46,'long',-95,'rot',30);

     ax1=subplot(2,2,1,'align');
      m_coast('patch','r');
      m_grid('xticklabel',[],'yticklabel',[],'linestyle','-','ytick',[-60:30:60]);
        
     ax2=subplot(2,2,2,'align');
      m_elev('contourf',[-7000:1000:0 500:500:3000],'edgecolor','none');
      colormap(ax2,[m_colmap('blues',70);m_colmap('gland',30)]);  
      caxis(ax2,[-7000 3000]);       
      m_grid('xticklabel',[],'yticklabel',[],'linestyle','-','ytick',[-60:30:60]);

        
     ax3=subplot(2,2,3,'align');
      colormap(ax3,[m_colmap('blues',70);m_colmap('gland',30)]);  
      caxis(ax3,[-7000 3000]);       
      m_elev('image');
      m_grid('xticklabel',[],'yticklabel',[],'linestyle','-','ytick',[-60:30:60]);

        
     ax4=subplot(2,2,4,'align');
      colormap(ax4,[m_colmap('blues')]);  
      caxis(ax4,[-8000 000]);       
      m_elev('shadedrelief','gradient',.5);
      m_coast('patch',[.7 .7 .7],'edgecolor','none');
      m_grid('xticklabel',[],'yticklabel',[],'linestyle','-','ytick',[-60:30:60]);

     ha = axes('Position',[0 0 1 1],'Xlim',[0 1],'Ylim',[0  1],'Box','off',...
               'Visible','off','Units','normalized', 'clipping' , 'off');
      text(0.5, 0.98,'This projection shows all oceans connected to each other',...
               'horiz','center','fontsize',20);
```

#### 15. Stepped jet colormap

```
    % get delta-SA data from the TEOS-10 gsw atlas at 2500 dbar

    [LG,LT]=meshgrid(0:360,-86:89);
    dSA=ones(size(LG));
    dSA(:)=gsw_deltaSA_atlas(3000*dSA(:),LG(:),LT(:));


    % Rearrange data to lie in the longitude limits I give for the
    % projection

    ind=[31:361 1:30]; % Move left side to right
    dSA=dSA(:,ind);
    LT=LT(:,ind);
    LG=LG(:,ind);LG(LG>30)=LG(LG>30)-360; %...and subtract 360 to some longitudes

    clf;
    m_proj('robinson','lon',[-330 30]);

    m_pcolor(LG,LT,dSA*1000);

    m_coast('patch',[.7 .7 .7],'edgecolor','none');
    m_grid('tickdir','out','linewi',2);

    % This is a perceptually uniform jet-like color scale, but in m_colmap
    % we can add some simple graduated steps to make the pcolor look a little
    % more like a contourf
    colormap(m_colmap('jet','step',10));

    h=colorbar('northoutside');
    title(h,'\deltaSA/(g/kg) at 2000 dbar','fontsize',14);
    set(h,'pos',get(h,'pos')+[.2 .05 -.4 0],'tickdir','out')

    set(gcf,'color','w');   % Need to do this otherwise 'print' turns the lakes black
```

#### 16. Bathymetry

```
load /ocean/rich/home/dens14/VENTS
lp=load('/ocean/rich/home/dens14/Linep_201402');
 
m_proj('lambert','long',[-160 -115],'lat',[32 60]);
[CS,CH]=m_etopo2('contourf',[-7000:1000:-1000 -500 -200 0 ],'edgecolor','none');
m_gshhs_f('patch',[.7 .7 .7],'edgecolor','none');
h1=m_line(vents.lon,vents.lat,'marker','s','color',[0 .5 0],...
          'linest','none','markerfacecolor','w','clip','point');
h2=m_line(lp.POS(:,2),lp.POS(:,1),'marker','o','color','r','linewi',2,...
          'linest','none','markersize',8,'markerfacecolor','w');
m_grid('linest','none','tickdir','out','box','fancy','fontsize',16);
legend([h1(1),h2(1)],'Known Hydrothermal vents','Line-P Stations','location','southwest');


colormap(m_colmap('blues'));  
caxis([-7000 000]);

[ax,h]=m_contfbar([.55 .75],.8,CS,CH,'endpiece','no','axfrac',.05);
title(ax,'meters')

set(gcf,'color','w');  % otherwise 'print' turns lakes black
```

#### 17. Shaded Relief (Example 1)

```
m_proj('lambert','lat',[5 24],'long',[105 125]);

set(gcf,'color','w')   % Set background colour before m_image call

caxis([-6000 0]);
colormap(flipud([flipud(m_colmap('blues',10));m_colmap('jet',118)]));
m_etopo2('shadedrelief','gradient',3);
 
m_gshhs_i('patch',[.8 .8 .8]);
 
m_grid('box','fancy');

ax=m_contfbar(.97,[.5 .9],[-6000 0],[-6000:100:000],'edgecolor','none','endpiece','no');
xlabel(ax,'meters','color','k');
```

#### 18. Shaded Relief (Example 2)

```
m_proj('utm','ellipse','grs80','zone',10,'lat',[49+15.7/60 49+21/60],...
        'long',[-123-15/60 -123-3/60]);      

% Uses multibeam bathymetry with 10m horizontal resolution
% Already regularly gridded in UTM coords with vector x2/y2, and
% matrix Z2.
caxis([-150 0]);
colormap([m_colmap('water',128)]);
m_shadedrelief(x2,y2,-Z2,'lightangle',-45,'gradient',8,'coord','z');

% Add some contours
hold on;

[cs,h]=contour(x2,y2,Z2,[0:20:150],'color','k');
clabel(cs,h,'fontsize',6);
hold off;

% Land parts from a previously saved high-resolution coastline
col=[255 214 140]/255; % CHS chart land colour

m_usercoast('/ocean/rich/more/mmapbase/bcgeo/PNW.mat','patch',col);
m_usercoast('/ocean/rich/more/mmapbase/bcgeo/PNWrivers.mat','patch',col);

% Lat/long AND a UTM grid
 
m_grid('tickdir','out','fontsize',12,'linest','none','xaxisloc','top','yaxisloc','right');
m_utmgrid('xcolor','b','ycolor','b','linest','-'); 

m_ruler([.5 .8],.9,'tickdir','out','ticklen',[.007 .007]);
m_northarrow(-123-4.5/60,49+19.5/60,1/60,'type',4,'aspect',1.5);

xlabel('Vancouver Harbour','color','k');
```

#### 19. Wind Roses

```
clf;set(gcf,'color','w'); 
 
m_proj('oblique','lon',[-125.3 -122.6],'lat',[50.1 48.6] ,'dir','vert','aspect',0.45)

% Database 1 coastline
m_usercoast('/ocean/rich/more/mmapbase/bcgeo/PNW.mat',...
            'patch',[.8 .8 .8],'edgecolor','k');
% Database 2 river
m_usercoast('/ocean/rich/more/mmapbase/bcgeo/PNWrivers.mat',...
            'patch',[.8 .8 .8],'edgecolor','k');
% Elevation database in structure SOGbath
hold on; m_contourf(SOGbath.lon,SOGbath.lat,SOGbath.Z,[500 500],...
             'edgecolor','none','facecolor',[.7 .7 .7]); hold off;

m_grid('linewi',2,'tickdir','out','ticklen',.03,'linestyl','none','color','k');
m_northarrow(-125.4,49.7,.2,'type',2);
title({'Winter','ONDJFM',' '},'fontsize',14);


% Wind data in struct arrays - pass each parameter to M_WINDROSE as a 1xN 
% cell array of data in the order you want them plotted (ones on top later
% in list)

levels=[0:4:24];  % Binning for speeds

k=[1 15  18 6 3 19 4 5 7 8 10 9 11 12 2 13 14 16 17 ]; % change order to 
                                                       % make overlaps look nicer

m_windrose({Winter(k).lon},{Winter(k).lat},{Winter(k).wdir},{Winter(k).wspd},...
           'size',.17,'nspeed',levels,'alpha',1);
colormap(m_colmap('jet'));
caxis([0 24]);

axb=m_contfbar(.8,[.6 .9],levels,levels);
axb.YLabel.String='Wind speeds m/s';
axb.YTick=levels;
```

#### 20. Colourmaps

```
 % This command generates the figure
 % In each subplot title is the M_COLMAP call that 
 % generated the colourmap displayed.
 %
 % Uses of these colourmaps can be seen
 % in other maps in this gallery.

 m_colmap demo
```

## Examples of satellite data manipulation

#### 1. Global SST (or any variable on a global Lat/Long grid)

```
 % NOAA/NASA Pathfinder AVHRR SST product
 % https://podaac.jpl.nasa.gov/sst/
 
 [P,map]=imread('../m_mapWK/199911h54ma-gdm.hdf');
 
 % Documentation for the 54km dataset gives
 % this formula for temperature
 P=0.15*double(P)-3; % deg C
 
 %...and defines this Lat/Long grid for the data
 Plat=90-.25-[0:359]*.5;Plon=-180+.25+[0:719]*.5;
 
 % Since the grid is rectangular in lat/long (i.e. not
 % really a projection at all, although it is included in
 % m_map under the name 'equidistant cyldindrical'), we 
 % don't want to use the 'image' technique. Instead... 
 % Create a grid, offsetting by half a grid point to account
 % for the flat pcolor
 [Plg,Plt]=meshgrid(Plon-0.25,Plat+0.25);
 
 m_proj('hammer-aitoff','clongitude',-150);
  
 % Rather than rearranging the data so its limits match the
 % plot I just draw it twice (you can see the join at 180W
 % because of the quirks of flat pcolor) (Note that 
 % all the global projections have 360 deg ambiguities)
 m_pcolor(Plg,Plt,P);shading flat;colormap(map);
 hold on;
 m_pcolor(Plg-360,Plt,P);shading flat;colormap(map);
 
 m_coast('patch',[.6 1 .6]);
 m_grid('xaxis','middle');

 % add a standard colorbar.
 h=colorbar('h');
 set(get(h,'xlabel'),'string','AVHRR SST Nov 1999');
```

#### 2. SSM/I Ice cover (data provided on a fixed grid) (HDF format)

```
%Near-Real-Time SSM/I-SSMIS EASE-Grid Daily Global Ice Concentration and 
% Snow Extent, Version 5
%
% Brodzik, M. J. and J. S. Stewart. 2016. Near-Real-Time SSM/I-SSMIS EASE-Grid 
% Daily Global Ice Concentration and Snow Extent, Version 5. Boulder, Colorado 
% USA. NASA National Snow and Ice Data Center Distributed Active Archive Center. 
% doi: https://dx.doi.org/10.5067/3KB2JPLFPK3R.

Q=hdfinfo('NISE_SSMISF18_20171124.HDFEOS')
%  Q.Attributes(2).Value says
%                UpperLeftPointMtrs=(-9036842.762500,9036842.762500)
%                LowerRightMtrs=(9036842.762500,-9036842.762500)
%                 ProjParams=(6371228,0,0,0,0,90000000,0,0,0,0,0,0,0)
%  and
%  Q.Attributes(3).Value says
%  'Data Value     Parameter
%     0              snow-free land
%     1-100          sea ice concentration percentage
%     101            permanent ice (Greenland, Antarctica)
%     102            not used
%     103            dry snow
%     104            wet snow
%     105-251        not used
%     252            mixed pixels at coastlines (unable to reliably apply microwave algorithm)
%     253            suspect ice value
%     254            corners(undefined)
%     255            ocean


P=hdfread('NISE_SSMISF18_20171124.HDFEOS','Northern Hemisphere','fields','Extent');
P(P==255)=105;   % Put ocean at top of indices
P(P>105)=0;

% According to web site this is is the projection info. I make te radius
% of my map less than the actual data field though.
m_proj('azimuthal equal-area','latitude',90,'radius',47,'rectbox','on');

 clf
% Plot data as an image
offs=9036842.762500/6371228; % Convert projection coords to units of earth radii
image([-offs offs],[offs -offs],P); set(gca,'ydir','normal');
colormap([.2 .5 .2;  % 0
           jet(100); % 1-100
           1 1 1;    % Greenland
           0 0 0  ;
           .9 .9 .9; % dry snow
           .8 .8 .8; % wet snow
           0 0 .5]); % 105 - now ocean
caxis([0 105]);

m_coast('color','k');
m_grid('linewi',2,'tickdir','out');
title({'SSM/I Ice cover Nov 24, 2017',''},'fontsize',14,'fontweight','bold');

hh=colorbar('h');
set(hh,'tickdir','out');
xlabel(hh,'% Ice cover');
```

#### 3. Aerial photos on an UTM grid

```
 % This image comes from the TerraServer
 % (http://terraserver.microsoft.com/ - now greatly altered)
 % and has been georeferenced to UTM coords. The UTM projection
 % uses UTM coordinates on the screen (as long as the ellipse
 % parameter is set to something other than the default),
 [P,map]=imread('../m_mapWK/oncehome.jpeg');
 
 % Set the projection limits to the lat/long of image
 % corners.
 m_proj('UTM','long',[-71-6/60-30/3600 -71-4/60-43/3600],...
             'lat',[42+21/60+13/3600  42+22/60+7/3600],'ellipse','wgs84');
 
 clf;
 image([326400 328800],[4692800 4691200],P);set(gca,'ydir','normal');
 m_grid('tickdir','out','linewi',2,'fontsize',14);
 title('A home for certain nerds','fontsize',16);
```

#### 4. A subset of a global dataset (HDF format)

```
  % Ocean colour data from http://seawifs.gsfc.nasa.gov/SEAWIFS.html
  %
  % Take a 4km weakly average dataset and plot a map for the Strait of
  % Georgia and outer coast. Note that most of this code is used
  % for reading in and subsetting the data.
  
  LATLIMS=[47 51];
  LONLIMS=[-130 -121];
 
  % Note - This is probably not the most efficient way to read and
  % handle HDF data, but I don't usually do this...
  %
  % First, get the attribute data
  PI=hdfinfo('../m_mapWK/A20040972004104.L3m_8D_CHLO_4KM');
  % And write it into a structure
  pin=[];
  for k=1:59
     nm=PI.Attributes(k).Name;nm(nm==' ')='_';
     if isstr(PI.Attributes(k).Value),
          pin=setfield(pin,nm,PI.Attributes(k).Value);
      else
         pin=setfield(pin,nm,double(PI.Attributes(k).Value));
      end
  end;
  
  % lon/lat of grid corners
  lon=[pin.Westernmost_Longitude:pin.Longitude_Step:pin.Easternmost_Longitude];
  lat=[pin.Northernmost_Latitude:-pin.Latitude_Step:pin.Southernmost_Latitude];
  
  % Get the indices needed for the area of interest
  [mn,ilt]=min(abs(lat-max(LATLIMS)));
  [mn,ilg]=min(abs(lon-min(LONLIMS)));
  ltlm=fix(diff(LATLIMS)/pin.Latitude_Step);
  lglm=fix(diff(LONLIMS)/pin.Longitude_Step);
  
  % load the subset of data needed for the map limits given  
  P=hdfread('../m_map/WKA20040972004104.L3m_8D_CHLO_4KM','l3m_data',...
            'Index',{[ilt ilg],[],[ltlm lglm]});
  
  % Convert data into log(Chla) using the equations given. Blank no-data.
  P=double(P);
  P(P==255)=NaN;
  P=(pin.Slope*P+pin.Intercept);   % log_10 of chla
 
  LT=lat(ilt+[0:ltlm-1]);LG=lon(ilg+[0:lglm-1]);
  [Plg,Plt]=meshgrid(LG,LT);
 
  % FINALLY....Draw the map...
  
  clf;
  m_proj('lambert','lon',LONLIMS,'lat',LATLIMS);
  m_pcolor(Plg,Plt,P);shading flat;
  m_gshhs_i('color','k');;
  m_grid('linewi',2,'tickdir','out');;
  h=colorbar;
  set(get(h,'ylabel'),'String','Chla (\mug/l)');
  set(h,'ytick',log10([.5 1 2 3 5 10 20 30]),'yticklabel',[.5 1 2 3 5 10 20 30],...
        'tickdir','out');
  title(['MODIS Chla ' ...
         datestr(datenum(pin.Period_Start_Year,1,0)+pin.Period_Start_Day) ...
         ' -> ' ...
         datestr(datenum(pin.Period_Start_Year,1,0)+pin.Period_End_Day) ],...
         'fontsize',14,'fontweight','bold');
```

#### 5. Meteorological data (netCDF format)

```
iday=156;   % the day to show

% use ncdisp(filename) to discover file contents...

lat=ncread('uwnd.10m.gauss.2017.nc','lat');
lon=ncread('uwnd.10m.gauss.2017.nc','lon');
[LN,LT]=meshgrid(lon,lat);

mtime=ncread('uwnd.10m.gauss.2017.nc','time')/24+datenum(1800,1,1,0,0,0);
u=ncread('uwnd.10m.gauss.2017.nc','uwnd',[1,1,iday],[192,94,1]);
v=ncread('vwnd.10m.gauss.2017.nc','vwnd',[1,1,iday],[192,94,1]);
prate=ncread('prate.sfc.gauss.2017.nc','prate',[1,1,iday],[192,94,1]);

m_proj('miller','lon',[100 260],'lat',[0 65]);
m_coast('patch',[.8 .8 .8]);
hold on 
[CS,CH]=m_contourf(LN,LT,prate'*1e3,[0.05:.05:.7],'edgecolor','none');
m_windbarb(LN,LT,u' ,v',2,'units','m/s','linewi',1,'color','r');
hold off;
m_grid('box','fancy','tickdir','out');

ax=m_contfbar([.3 .7],.05,CS,CH);
set(ax,'fontsize',12)
xlabel(ax,'Mean Daily Precipitation Rate/(kg/m^2/s)');

title(['North Pacific Surface Winds : ' datestr(mtime(iday))],'fontsize',16);

colormap(flipud(m_colmap('Blues')))
```

#### 6. ARGO drifter tracks (netCDF format)

```
% Argo stuff
% Go to:
%         https://www.usgodae.org/cgi-bin/argo_select.pl
% Select area you want and date range, download all profiles
% tar -xvzf them into directory...
basname='./argo';

% base map
m_proj('lambert','lons',[-150 -124],'lat',[40 60],'rectbox','on');
[cs,h]=m_etopo2('contourf',[-7000:500:0],'edgecolor','none');
m_gshhs_l('patch',[.5 .8 0],'edgecolor','none');
m_grid('linewi',2,'layer','top');
caxis([-7000 000]);
m_contfbar(.92,[.2 .5],cs,h,'endpiece','no','axfrac',.02);
colormap(m_colmap('blue'));   
title('Argo float trajectories NE Pacific (2017)');

% Add ARGO float trajectories
%  1) draw a scale arrow
vecscl=0.015;
m_vec(vecscl ,-126,58,-0.01,0,'r','shaftwidth',2,'headlength',10,...
      'key',{'1 cm/s','Mean Drift'},'centered','yes');
 
dirs=dir(basname);
m=0;
for k=3:length(dirs)
   profname=dir([dirs(k).folder '/' dirs(k).name]);
   for l=3:length(profname)
       fname=[profname(l).folder '/' profname(l).name '/' profname(l).name '_Rtraj.nc'];
       %ncdisp(fname);
       
       argo.id=ncread(fname,'PLATFORM_NUMBER');
       argo.mtime=ncread(fname,'JULD')+datenum(1950,1,1);
       argo.lat=ncread(fname,'LATITUDE');
       argo.lon=ncread(fname,'LONGITUDE');
        
       % Long are stored between -180 and +180; this removes artificial
       % jumps which might happen just left of the map limits.
       argo.lon(argo.lon>0)=argo.lon(argo.lon>0)-360;
       
        
       ii=find(isfinite(argo.lon));
       if any(ii )
          m_line(argo.lon(ii),argo.lat(ii),'color',[0 0 0]);
          
          % Sometimes first point is an error of some sort so skip it
          Dlon=argo.lon(ii([2 end]));
          Dlat=argo.lat(ii([2 end]));
          t=diff(argo.mtime(ii([2 end]))); % time between the points
         
          % Distance between the points
          [d,a12]=m_idist(Dlon(1),Dlat(1),Dlon(2),Dlat(2));
          
          % Store stuff
          m=m+1;
          spd(m)=d/(t*86400) ;% m/s 
                   
          % Find midpoint on geodesic and store as well
          [Clon(m),Clat(m),a21(m)]=m_fdist(Dlon(1),Dlat(1),a12,d/2);
        end
   end
          
end
Clon=rem(Clon-360,360); % Get it into the right range
a21=rem(a21-180,360);   % I need the opposite direction

% Draw all the 'mean speed' arrows, centered at the midpoint of the
% geodesic between first and last points.
m_vec(vecscl ,Clon,Clat,spd.*sind(a21),spd.*cosd(a21),'r',...
           'centered','yes','shaftwidth',2,'headlength',10);
```

#### 7. SAR image of internal waves (HDF-5 format)

```
fname='SAR_IMP_1PNESA19920724_190439_00000018C086_00199_05354_0000.h5';
%h5disp(fname)   % See the structure

titlestr=h5readatt(fname,'/metadata/MPH','STATE_VECTOR_TIME');
datsize=double([ h5readatt(fname,'/bands/Amplitude','raster_width') ...
                 h5readatt(fname,'/bands/Amplitude','raster_height')]);

tielat=h5read(fname,'/tie_point_grids/latitude');
tielon=h5read(fname,'/tie_point_grids/longitude');
stp=[h5readatt(fname,'/tie_point_grids/latitude','sub_sampling_x') ...
     h5readatt(fname,'/tie_point_grids/latitude','sub_sampling_y') ];

% Pull out a subsection in by [2000 2500] from one corner and in 
%  [600 2000] from the opposite corner
istart=[2000 2500];
strd=[3 3];
cnt=fix([(datsize(1)-istart(1)-600)/strd(1) (datsize(2)-istart(2)-2000)/strd(2)]);
% ...and read.
subimg=h5read(fname,'/bands/Amplitude',istart,cnt,strd);

% ....smooth it a bit...
subf=filter2(ones(3,3)/9,subimg);

% Now generate lat/lon for all pixels by interpolating from
% the tie points.
Ty=[0:size(tielat,2)-1]*stp(2)+1;
Tx=[0:size(tielat,1)-1]*stp(1)+1;
Iy=istart(2)+[0:size(subimg,2)-1]*strd(2);
Ix=istart(1)+[0:size(subimg,1)-1]*strd(1);
sublat=interp2(Ty',Tx,tielat,Iy',Ix);
sublon=interp2(Ty',Tx,tielon,Iy',Ix);


% Now make the map

m_proj('lambert','lon',[-123-25/60 -122-40/60],'lat',[48+42/60 49+9/60]);
m_pcolor(sublon,sublat,subf);shading flat;
m_grid('box','fancy','tickdir','out');
m_ruler(1.03,[.15 .5],'ticklen',[.01]);
caxis([0 450]);
colormap(gray);
title(titlestr)
```

#### 8. Shaded Relief from a high-resolution topography (netCDF format)

```
clat=[48+46/60 49+5/60];clon=[-125-15/60 -124-55/60]; % Barkley Sound

%% Read data from a netcdf file
fname='/ocean/rich/more/mmapbase/noaa_bc3/barkley_sound_1_navd88_2016.nc';
lat=ncread(fname,'lat');
lon=ncread(fname,'lon');
ilat=lat>=clat(1) & lat<=clat(2);
ilon=lon>=clon(1) & lon<=clon(2);
Z=ncread(fname,'Band1',[ find((ilon),1,'first') find((ilat),1,'first')],...
                       [ sum(ilon) sum(ilat)],[1 1]);


m_proj('equidistant','lon',clon,'lat',clat);   % Projection

%   The shaded relief version (right hand plot))

subplot(1,2,2);
caxis([-300 1210]);  % 1210 chosen by manual adjustment
                     % since  'waterline" appears to be at about Z=2 (vertical datum for
                     % bathymetry is 'lowest normal tide')
colormap([m_colmap('blues',32);m_colmap('gland',128)]);   % Colormap sizes chosen because...
                                                          % ... 32/128 = (300+2)/(1210-2)

m_shadedrelief(lon(ilon),lat(ilat),Z');   

m_grid('box','fancy','grid','none','fontsize',14);

m_contfbar( [.3 .7],.98, Z',[-300:1210],...
            'axfrac',.02,'endpiece','no','levels','match','edgecolor','none');          

% The normal contour version (left hand plot))

subplot(1,2,1);
m_contourf(lon(ilon),lat(ilat),Z',[-300:25:-25 2 50:50:200 300:100:1200 1210]);

caxis([-300 1210]);   
colormap([m_colmap('blues',32);m_colmap('gland',128)]);

m_grid('box','fancy','tickdir','out','grid','none','fontsize',14);

m_contfbar( [.3 .7],.98, Z',[-300:25:-25 2 50:50:200 300:100:1200 1210],...
            'axfrac',.02,'endpiece','no','levels','match');
```

#### 9. Google Maps Static API

```
% Axis limits 
lms=[ -123-[14 8]/60  49+[10 13.5]/60 ];   

% plot_google_maps.m from the mathworks user contributed files database:
% https://www.mathworks.com/matlabcentral/fileexchange/27627-zoharby-plot_google_map
% accesses the static google maps map tiles, returning a lat/long gridded
% image that is just bigger than the given limits.
clf;
axis(lms);
[Glon,Glat,Gimg]=plot_google_map('maptype','satellite','refresh',0,'autoaxis',0);
clf;

% Now draw it!
 
m_proj('utm','lat',lms(3:4),'lon',lms(1:2));
m_image(Glon,Glat,Gimg); 
m_grid('tickdir','out','box','fancy');
m_ruler([.4 .9],1.05,6,'tickdir','in','ticklen',.01);
```


---

## Citation

If you decide that M\_Map made a significant contribution to a project, and you want
acknowledge that contribution with a citation (thanks!), I recommend something like the following:

- Pawlowicz, R., 2020. "M\_Map: A mapping package for MATLAB", version 1.4m, [Computer software], available
  online at www.eoas.ubc.ca/~rich/map.html.

---

## Acknowledgements

A number of people have helped out with suggestions, code fixes,
etc. I am especially grateful for the work done by E. Firing, D.
Byrne, M. Mann, J. Pringle, J. E. Nilsen, M.Halverson, Jamie Tsoa, Shi Weiheng, and W. Brown who have all
contributed code.
