# Supplementary material for: Excursions in the Bayesian treatment of model error
Source: PLoS One. 2023 Jun 2;18(6):e0286624. doi: 10.1371/journal.pone.0286624 (PMC10237458; doi:10.1371/journal.pone.0286624)
Supplement: S1 Data — The file suporting_information.zip contains the MATLAB code and the synthetic data that was used to produce all the figures in this manuscript. (ZIP) [file pone.0286624.s001.zip › Figure1/m_map/mapug.html]

M\_Map Users Guide


- Introduction
- Gallery
- Getting M\_Map
- Release Notes
- Users Guide
- Example Code
- Citation
- Acknowledgements

Last changed 20/Apr/2021. Questions and comments to rich@eos.ubc.ca

# M\_Map:

## Users Guide v1.4

---


## Table of Contents

(Note - a Chinese translation of an earlier version of this guide can be
found here)

1. Getting started
2. Specifying projections
   1. Azimuthal projections
   2. Cylindrical and Pseudo-cylindrical projections
   3. Conic projections
   4. Miscellaneous global projections
   5. Yeah, but which projection should I use?
   6. Map scales
   7. Map coordinate systems - geographic and geomagnetic
3. Coastlines and Bathymetry
   1. Coastline options
   2. Topography/Bathymetry options
4. Customizing the axes
   1. Grid lines and labels
   2. Titles and x/ylabels
   3. Legend Boxes
   4. Scale bar
   5. North Arrow
5. Adding your own data
   1. Drawing lines, text,
      arrows, patches, hatches, speckles, and contours
   2. Drawing images and pcolor
   3. Drawing shaded relief maps
   4. Drawing tracklines
   5. Drawing range rings and geodesics
   6. Drawing tidal ellipses and wind roses
   7. Converting longitude/latitude to projection coordinates
   8. Converting projection to longitude/latitude coordinates
   9. Computing distances between points
   10. Annotation and mouse input
   11. Colour and Colormaps
   12. Colourbars with Contourmaps
6. More complex maps
7. Removing features from a map
8. Adding your own coastlines
   1. Reading and Handling coastline data
   2. ESRI Shapefiles
   3. Projection conversions
   4. Coastline Extractor
   5. DCW political boundaries
   6. Natural Earth political boundaries
   7. GSHHS(G) high-resolution coastline database
      1. Installing GSHHS
      2. Using GSHHS effectively- Adding your own topography/bathymetry
     1. Sandwell and Smith Bathymetry
     2. TerrainBase 5-minute global bathymetry/topography
     3. ETOPO 2- and 1- minute global bathymetry/topography
   - M\_Map toolbox contents and description
   - Known Problems and Bugs
   - OCTAVE Compatibility Issues
   - Changes since last release

---

## 1. Getting started

First, get all the files, either as a  zip archive or
a  gzipped
tar-file and unpack them. If you are unpacking the zip file MAKE
SURE YOU ALSO UNPACK SUBDIRECTORIES! Now, start up Matlab (version 5 or
higher). Make sure that the toolbox is in your path. This can be done
simply by `cd`'ing to the correct directory.

Alternatively, if you have unpacked them into directory `/users/rich/m_map`
(and `/users/rich/m_map/private`), then you can add this to
your search path:

```
path(path,'/users/rich/m_map');
```

or

```
addpath /users/rich/m_map
```

To follow along with this document, you would then use a Web-browser to
open `file:/users/rich/m_map/map.html`,
that is, this HTML document.

Note: you may want to install M\_Map as a toolbox accessible to all
users. To do this, unpack the files into `$MATLAB/toolbox/m_map`, add
that directory to the list defined in  `$MATLAB/toolbox/local/pathdef.m`, and
update the cache file using

```
rehash toolboxcache
```

Instructions for installing an (optional) high-resolution
bathymetry database are given in  here, and
instructions
for installing the (optional) high-resolution GSHHS coastline database
is given in  here. However, we should first
check that the basic setup is OK.

To see an example map, try this:

```
m_proj('oblique mercator');
m_coast;
m_grid;
```

This is a line map of the Oregon/British Columbia coast, using an
Oblique Mercator projection (A few more complex maps can be generated
by running the demo function `m_demo`).

The first line initializes the projection. Defaults are set for the
different projection, so you can easily see what a specific projection
looks like, but all projections have a number of optional parameters as well.
To get the same map without using the defaults, you would use

```
m_proj('oblique mercator','longitudes',[-132 -125], ...
           'latitudes',[56 40],'direction','vertical','aspect',.5);
```

The exact meanings of the various options is given in 
Section 2. However, notice that longitudes are specified using a *signed*  notation - East longitudes are positive, whereas West
longitudes are negative (Also note that a decimal degree notation
is used, so that a longitude of 120 30'W is specified as -120.5).

The second line draws a coastline, using the 1/4 degree database.
Coastlines with greater resolution can be drawn, using your own
database (see also  Section 8). `m_coast`
can be called with various line parameters. For example,

```
m_coast('linewidth',2,'color','r');
```

draws a thicker red coastline. Filled coastlines can also be drawn,
using the  `'patch'`  option (followed by any of the usual
PATCH property/value pairs:

```
m_coast('patch',[.7 .7 .7],'edgecolor','none');
```

draws a coastline with a gray fill and no border.

The third line superimposes a grid. Although there are many
possible options that can be used to customize the appearance of the
grid, defaults can always be used (as in the example). These options
are discussed in  Section 4. You can get a list of
the options using the GET syntax: 

```
m_grid get
```

which acts somewhat like the  `get(gca)`  syntax for
regular plots.

Finally, suppose you want to show and label the location of, say, a
mooring at 129W, 48 30'N.

```
[X,Y]=m_ll2xy(-129,48.5);
line(X,Y,'marker','square','markersize',4,'color','r');
text(X,Y,' M5','vertical','top');
```

`m_ll2xy`  (and its inverse `m_xy2ll`)
convert from longitude/latitude coordinates to those of the projection.
Various clipping options can also be specified in converting to
projection coordinates. If you are willing to accept default clipping
setting, you can use the
built-in functions  `m_line`  and  `m_text` :

```
m_line(-129,48.5,'marker','square','markersize',4,'color','r');
m_text(-129,48.5,' M5','vertical','top');
```

Finally (!), we may want to alter the grid details slightly. Note
that, a given map must only be initialized once.

```
clf
m_proj('oblique mercator');  % repeated here so cut-n-paste simplified
m_coast('patch',[.7 .7 .7],'edgecolor','none');
m_grid('xlabeldir','end','fontsize',10);
m_line(-129,48.5,'marker','square','markersize',4,'color','r');
m_text(-129,48.5,' M5','vertical','top');
```


---

## 2. Specifying projections

In order to get a list of the current projections,

```
m_proj get
```

or

```
m_proj('set');
```

Which currently return the following list:

```
Available projections are:
     Stereographic          
     Orthographic 
     Azimuthal Equal-area    
     Azimuthal Equidistant
     Gnomonic
     Satellite
     Albers Equal-Area Conic
     Lambert Conformal Conic
     Mercator
     Miller Cylindrical
     Equidistant Cylindrical
     Cylindrical Equal-Area
     Oblique Mercator
     Transverse Mercator
     Sinusoidal
     Gall-Peters
     Hammer-Aitoff
     Mollweide
     Robinson
     UTM
```

If you want details about the possible options for any of these
projections, add its name to the above command, e.g.

```
m_proj('set','stereographic');
```

which returns

```
     'Stereographic'                                   
     <,'lon<gitude>',center_long>                      
     <,'lat<itude>', center_lat>                       
     <,'rad<ius>', ( degrees | [longitude latitude] )>
     <,'rec<tbox>', ( 'on' | 'off' )>
```

You can also get details about the current projection. For example, in
order to see what the default parameters are for the sinusoidal
projection, we first initialize it, and then use the  `'set'` 
option:

```
m_proj('sinusoidal');
m_proj get

Current mapping parameters -
  Projection: Sinusoidal  (function: mp_tmerc)
  longitudes: -90  30 (centered at -30)    
  latitudes: -65  65             
  Rectangular border: off
```

In order to initialize a projection, you usually specify some location
parameters that define the geometry of the projection (longitudinal
limits, central parallel, etc.), as well as parameters that define the
extent
of the map (whether it is in a rectangular axis, what the border points
are, etc.). These vary slightly from projection to projection.

Two useful properties for projections are (1) the ability the
preserve angles for differentially small regions, and (2) the ability
to preserve area. Projections satisfying the first condition are called
 *conformal*, those satisfying the second are called *equal-area*. No projection can be both. Many projections (especially global
projections) are neither, instead an attempt has been made to aesthetically balance
the errors in both conditions.

Note: Most projections are currently  *spherical* 
rather than ellipsoidal. UTM is an ellipsoidal projection, and both the lambert conformal
conic and albers equal-area conic can be specified with ellipses if desired. This
is sometime useful when you have data (e.g. from a GIS package) at scales of Canadian provinces
or US states, which are often mapped using one of these projections.
It is unlikely that using a spherical earth model will be a problem (or an advantage) in
normal usage.

Let's go through the list of available projections:

### - Azimuthal projections

Azimuthal projections are those in which points on the
globe are projected onto a flat tangent plane. Maps using these
projections have the property that direction or azimuth from the center
point to all other points is shown correctly. Great circle routes
passing through the central point appear as straight lines (although
great circles not passing through the central point may appear curved).
These maps are usually drawn with circular boundaries. The following
parameters specify the center point of an azimuthal projection map:

```
  <,'lon<gitude>',center_long>
  <,'lat<itude>', center_lat>
```

Maps are aligned so that the specified longitude is vertical at
the map center, with its northern end at the top (but see option `rotangle`
below in order to rotate this orientation). Then the extent of the map
is defined by

```
   <,'rad<ius>', ( degrees | [longitude latitude] )>
```

Either an angular
distance in degrees can be given (e.g. 90 for a hemisphere), or the
coordinates of a point on the boundary can be specified. Then,

```
  <,'rec<tbox>', ( 'on' | 'off' | 'circle' )>
```

is used to specify the map boundary. The default is to enclose the map
in a circular boundary (chosen using either of the latter two options),
but a rectangular one can also be specified. However, rectangular maps
are usually better drawn using a cylindrical or conic projection of some sort.
Finally,

```
   <,'rot<angle>', degrees CCW>
```

rotates the figure so that the central longitude
is not vertical.

THe azimuthal projections include:

#### - Stereographic

The stereographic projection is conformal, but not
equal-area. This projection is often used for polar regions.

#### - Orthographic

This projection is neither equal-area nor conformal,
but resembles a perspective view of the globe.

#### - Azimuthal Equal-Area

Sometimes called the Lambert azimuthal equal-area
projection, this mapping is equal-area but not conformal.

#### - Azimuthal Equidistant

This projection is neither equal-area nor conformal,
but all distances and directions from the central point are true.

#### - Gnomonic

This projection is neither equal-area nor conformal,
but all straight lines on the map (not just those through the center)
are great circle routes. There is, however, a great degree of
distortion at the edges of the map, so maximum radii should be kept
fairly small - 20 or
30 degrees at most.

#### - Satellite

This is a perspective view of the earth, as seen by
a satellite at a specified altitude. Instead of specifying a `radius`
for the map, the viewpoint altitude is specified:

```
 <,'alt<itude>',altitude_fraction >
```

the numerical value assigned to this property
represents the height of the viewpoint in units of earth radii. For
example, a satellite in an orbit of radius 3 earth radii would have an
altitude of
2.

### - Cylindrical and Pseudo-cylindrical Projections

Cylindrical projections are formed by projecting
points onto a plane wrapped around the globe, touching only along some
great circle. These are very useful projections for showing regions of great
lateral extent, and are also commonly used for global maps of
mid-latitude regions only. Also included here are two pseudo-cylindrical
projections, the sinusoidal and Gall-Peters, which have some similarities to the
cylindrical projections (see below).

These maps are usually drawn with rectangular
boundaries (with the exception of the sinusoidal and sometimes the
transverse mercator).

#### - Mercator

This is a conformal map, based on a tangent cylinder
wrapped around the equator. Straight lines on this projection are rhumb
lines (i.e. the track followed by a course of constant bearing). The
following properties affect this projection: 

```
 <,'lon<gitude>',( [min max] | center)>
```

Either longitude limits can be set, or a central
longitude defined implying a global map.

```
 <,'lat<itude>', ( maxlat | [min max])>
```

Latitude limits are most usually the same in
both N and S latitude, and can be specified with a single value, but
(if
desired) unequal limits can also be used. DO NOT USE MERCATOR FOR A MAP THAT DOES NOT CONTAIN THE EQUATOR!!!

#### - Miller Cylindrical

This projection is neither equal-area nor conformal,
but "looks nice" for world maps. Properties are the same as for the
Mercator, above.

#### - Cylindrical Equal-Area

An equal-area projection. You really shouldn't use this one, since it greatly
distorts shapes near the poles, but it is included here for completeness.

#### - Equidistant cylindrical

This projection is neither equal-area nor conformal.
It consists of equally-spaced latitude and longitude lines, and is very
often used for quick plotting of data. It is included here simply so
that such maps can take advantage of the grid generation routines. Also
known as the Plate Carree. Properties are the same as for the Mercator, above.

#### - Oblique Mercator

The oblique mercator arises when the great circle
of tangency is arbitrary. This is a useful projection for, e.g., long
coastlines or other awkwardly shaped or aligned regions. It is
conformal but not equal area. The following properties govern this
projection:

```
    <,'lon<gitude>',[ G1 G2 ]>  
    <,'lat<itude>', [ L1 L2 ]>
```

Two points specify a great circle, and thus the
limits of this map (it is assumed that the region near the shortest of
the two arcs is desired). The 2 points (G1,L1) and (G2,L2) are thus at
the center of either the top/bottom or left/right sides of the map
(depending on the `'direction'` property).

```
   <,'asp<ect>',value>
```

This specifies the size of the map in the direction
perpendicular to the great circle of tangency, as a proportion of the
length shown. An aspect ratio of 1 results in a square map, smaller
numbers result in skinnier maps. Aspect ratios >1 are possible, but not
recommended.

```
    <,'dir<ection>',( 'horizontal' | 'vertical' )
```

This specifies whether the great circle of tangency
will be horizontal on the page (for making short wide maps), or
vertical (for tall thin maps).

WARNING - at SOME times, for certain combinations of G1/G2 L1/L2 endpoints, the coastline mapping algorithms fail
because of a numerical instability affecting the mapping of points on the opposite side of the world - you can see weird lines
going across your map when you try to plot a coastline. The work-around for this is to alter the G1/G2 or L1/L2 slightly.

#### - Transverse Mercator

The Transverse Mercator is a special case of the
oblique mercator when the great circle of tangency lies along a
meridian of longitude, and is therefore conformal. It is often used for
large-scale maps and charts. The following properties govern this projection:

```
    <,'lon<gitude>',[min max]> 
    <,'lat<itude>',[min max]>
```

These specify the limits of the map.

```
    <,'clo<ngitude>',value>
```

Although it makes most sense in general
to specify the central meridian as the meridian of tangency (this is
the default), certain map classification systems (noteably UTM) use only a
fixed set of central longitudes, which may not be in the map center.

```
    <,'rec<tbox>', ( 'on' | 'off' )>
```

The map limits can either be based on
latitude/longitude (the default), or the map boundaries can form an
exact rectangle. The difference is small for large-scale maps. Note: Although this
projection is similar to the Universal Transverse Mercator (UTM) projection, the
latter is actually ellipsoidal in nature.

#### - Universal Transverse Mercator (UTM)

UTM maps are needed only for high-quality maps of
small regions of the globe (less than a few degrees in longitude). This
is an ellipsoidal projection. Options are similar to those of the
Transverse Mercator, with the addition of

```
     <,'zon<e>', value 1-60> 
    
     <,'hem<isphere>',value 0=N,1=S>
```

These are computed automatically if not specified.
The ellipsoid defaults to `'normal'`, a spherical earth of
radius 1 unit, but other options can also be chosen using the following
property:

```
    <,'ell<ipsoid>', ellipsoid>
```

For a list of available ellipsoids try `m_proj('set','utm')`.

The big difference between UTM and all the
other projections is that for ellipsoids other than `'normal'`
the projection coordinates are in meters, so-called "easting" and "northing". To take full
advantage of this it is often useful to call `m_proj` with `'rectbox'`
set to `'on'` and not to use the long/lat grid generated
by `m_grid` (since the regular matlab grid will be in units
of meters).

#### - Sinusoidal

This projection is usually called
"pseudo-cylindrical" since parallels of latitude appear as straight
lines, similar to their appearance in cylindrical projections tangent
to the equator. However, meridians curve together in this projection in
a sinusoidal way (hence the name), making this map equal-area.

#### - Gall-Peters

Parallels of latitude and meridians both appear as
straight lines, but the vertical scale is distorted so that area is
preserved. This is useful for tropical areas, but the distortion in
polar areas is extreme.

### - Conic Projections

Conic projections result from projecting onto a cone
wrapped around the sphere. The vertex of the cone lies on the
rotational axis of the sphere. The cone is either tangent at a single
latitude, or can intersect the sphere at two separated latitudes. It is
a useful projection for mid-latitude areas of large east-west extent.
The following properties affect these projections:

```
  <,'lon<gitude>',[min max]> 
  <,'lat<itude>',[min max]>
```

These specify the limits of the map.

```
  <,'clo<ngitude>',value>
```

The central longitude appears as a vertical on the
page. The default value is the mean longitude, although it may be set
to any value (even one outside the limits).

```
  <,'par<allels>',[lat1 lat2]>
```

The standard parallels can be specified. Either one or
two parallels can be given:

```
  <,'rec<tbox>', ( 'on' | 'off' )>
```

The default is a parallels at 1/3 and 2/3 of the latitudinal range.

The map limits can either be based on
latitude/longitude (the default), or the map boundaries can form an
exact rectangle which contain the given limits. Unless the region being mapped is small, it
is best to leave this  `'off'` .

The default is to use a spherical earth model for the mapping transformations. However, ellipsoidal coordinates can
also be specified. This tends to be useful only for doing coordinate transformations (e.g., if a particular
gridded database in in this kind of projection, and you want to find lat/long data), since
the difference would be impossible to see by eye on a plot.
The particular ellipsoid used can be chosen using the following
property:

```
    <,'ell<ipsoid>', ellipsoid>
```

For a list of available ellipsoids try `m_proj('set','albers')`.

Finally, if you just want to use `M_Map` as an engine to transform
between projected coordinates in some database and lat/long, it is useful to be able to explicitly specify the origin of the coordinate
system that was originally used (origins are sometimes set at the lower boundary of a projection so that all Y values are positive). This can be done be setting:

```
    <,'ori<gin>', [long lat]>
```

But note that "false eastings" and "false northings" are not handled by `m_proj`, instead you must
correct for them explicitly using

```
     [long,lat]=m_xy2ll(x-false_easting,y-false_northing);
```

if you are trying to do this.

#### - Albers Equal-Area Conic

This projection is equal-area, but not conformal

#### - Lambert Conformal Conic

This projection is conformal, but not equal-area.

### - Miscellaneous global projections

There are a number of projections which don't really
fit into any of the above categories. Mostly these are global
projections (i.e. they show the whole world), and they have been
designed to be "pleasing to the eye". I'm not sure what use they are in
general, but they make
nice logos!

#### - Hammer-Aitoff

An equal-area projection with curved meridians and
parallels.

#### - Mollweide

Also called the Elliptical or Homolographic
Equal-Area Projection. Parallels are straight (and parallel) in this
projection.

Note that example 4 shows a rather
sophisticated use for viewing the oceans, designed to reduce distortion.

A more standard map
can be made using

```
    m_proj('mollweide');
    m_coast('patch','r');
    m_grid('xaxislocation','middle');
```

#### - Robinson

Not equal-area OR conformal, but supposedly "pleasing to the eye".

### - Yeah, but which projection should I use?

Well, it depends really on how large an area you are
mapping. Usually, maps of the whole world are Mercator, although often
the Miller Cylindrical projection looks better because it doesn't
emphasize the polar areas as much. Another choice is the Hammer-Aitoff
or Mollweide (which has meridians curving together near the poles).
Both are equal-area. It's probably not a good idea to use these
projections for maps that don't have the equator somewhere near the
middle. The Robinson projection is not equal-area or conformal, but was the choice of National Geographic (for a while, anyway),
and also appears in the IPCC reports.

If you are plotting something with a large
north/south extent, but not very wide (say, North and South America, or
the North
and South Atlantic), then the Sinusoidal or Mollweide projections will
look pretty good. Another choice is the Transverse Mercator, although
that
is usually used only for very large-scale maps (i.e., ones covering a very small area).

For smaller areas within one hemisphere or other
(say, Australia, the United States, the Mediterranean, the North
Atlantic) you might pick a conic projection. The differences between
the two available conic projections are subtle, and if you don't know
much about projections it probably won't make much difference which one
you use.

If you get smaller than that, it doesn't matter a
whole lot which projection you use. One projection I find useful in
many cases is the Oblique Mercator, since you can align it along a long
(but narrow) coastal area. If map limits along lines of
longitude/latitude are OK, use a Transverse Mercator or Conic
Projection. The UTM projection is also useful if you want to make a grid in meters, since the
projection coordinates (i.e., "eastings and northings") are in meters.

Polar areas are traditionally mapped using a
Stereographic projection, since everyone seems to think it looks nice to have a
"bullseye" pattern of latitude lines (the fact that lines of longitude come together at the poles also makes them
an almost irresistable destination for polar scientists and explorers).

If you want to get a quick idea of what any
projection looks like, it is simple to do so - default parameters for all functions are set for
a "typical" usage. For example, to see a stereographic map, try:

```
  m_proj('stereographic');  % Example for stereographic projection
  m_coast;
  m_grid;
```

### - Map scales

M\_Map usually scales the map so that it fits exactly
within the current axes. If you just want a nice picture (which is
mostly the case) then this is exactly what you need. On the other hand,
sometimes you want to print things out at some exact scale (i.e. if you
really much prefer sitting at your desk with a ruler and a piece of
paper trying to figure out how far apart Bangkok and Tokyo are). Use
the `m_scale` primitive for this - for a 1:250000 map, call

```
  m_scale(250000);
```

after you have drawn everything (Be careful - a
1:250000 map of the world is a lot bigger than 8.5"x11" sheet of
paper).

This option is usually only useful for
large-scale maps, i.e. maps of very small areas).

If you wish to know the current scale, calling `m_scale`
without any parameters will calculate and return that value.

To return to the default scaling call `m_scale('auto')`.

(PS - If you do want to find distances from Bangkok
to anywhere, plot an azimuthal equidistant projection of the world
centered on Bangkok (13 44'N, 100 30'E), and choose a fairly small
scale, like 1:200,000,000). Another option would be to use range rings, see example 11.

### - Map coordinate systems - geographic and geomagnetic.

Latitude/Longitude is the
usual coordinate system for maps. In some cases UTM coords are also
used, but these are really just a simple transformation based on the
location of the equator and certain lines of longitude. On the other
hand, there are occasions when a coordinate system based on some other
set of axes is useful. For example, in space
physics data is often projected in coordinates based on the magnetic
poles. M\_Map has a limited capability to deal with data in these other
coordinate systems. `m_coord` allows you to change the coordinate system from
geographic to geomagnetic, using data provided by the 
13th International Geomagnetic Reference Field (IGRF-13).

The following code gives you the idea - grids and boxes
are shown in the two different coordinate systems:

```
lat=[25*ones(1,100) 50*ones(1,100) 25]; % Outline of a box
lon=[-99:0 0:-1:-99 -99];

subplot(121);
m_coord('IGRF-geomagnetic',datenum(2000,1,1)); % Treat all lat/longs as geomagnetic
                                               % with coordinate system for the year 
                                               % 2000
m_proj('stereographic');
m_coast('patch',[.5 1 .5],'edgecolor','none');
m_grid;
m_line(lon,lat,'color','r','linewi',3);   % "lat/long" assumed geomagnetic ...
                                          %     ... on the geomagnetic map
m_coord('geographic');                    % Switch to assuming geographic
m_line(lon,lat,'color','b','linewi',3');  % Now they are treated as geographic
  
subplot(122); 
m_coord('geographic');                    % Define all in geographic 
m_proj('stereographic'); 
m_coast('patch',[.5 1 .5],'edgecolor','none'); 
m_grid; 
m_line(lon,lat,'color','b','linewi',3); 
m_coord('IGRF-geomagnetic',datenum(2000,1,1)); % Now specify geomagnetic coords
m_line(lon,lat,'color','r','linewi',3);
```


Note that this option is not
used very much, hence is not fully supported. In particular, filled
coastlines may not work properly in all projections.

If you just want to convert a set of coordinates between geographic and geomagnetic,
you can do this without setting a projection first as long as you call `m_coord` to
define the geomagnetic coordinate system.
For example:

```
m_coord('IGRF-geomagnetic',datenum(2019,6,1));  % define the coord system
[gLON,glAT]=m_geo2mag(LON,LAT);   % Convert geographic to geomagnetic
[LON,LAT]=m_mag2geo(gLON,gLAT);   % This is the inverse
```

---

## 3. Coastlines and Bathymetry

M\_Map includes two fairly simple databases for coastlines and
global elevation data. Highly-detailed databases are not included in
this release because they are a) extremely large and b) extremely
time-consuming to
process (loops are inherently involved). If more detailed maps are
required,  section 8  and  section 9 
give instructions on how to add some freely-available high-resolution
datasets.


## - Coastline options

M\_Map includes a 1/4 degree resolution coastline database. This
is suitable for maps covering large portions of the globe, but is
noticeably coarse for many large-scale applications. The M\_Map
database is accessed using the  `m_coast`  function.
Coastlines can be drawn as simple lines,
using

```
  m_coast('line', ...optional line arguments );
```

or

```
  m_coast( optional line arguments );
```

where the optional arguments are all the standard arguments for
specifying line style, width, color, etc. Coastlines can also be drawn
as filled
patches using

```
  m_coast('patch', ...optional patch arguments );
```

where the optional trailing arguments are the standard patch
properties. For example,

```
  m_coast('patch',[.7 .7 .7],'edgecolor','g');
```

draws gray land, outlined in green. When patches are being drawn, lakes
and inland seas are given the axes background colour.

Many older (ocean) maps are created with speckled land
boundaries, which looks
very nice in black and white. You can get a speckled boundary with

```
  m_coast('speckle', ....optional m_hatch arguments);
```

which calls `m_hatch`. This only looks nice if there aren't
too many
very tiny islands and/or lakes in the image (see Example 13).

Note that line coastlines are usually drawn rather rapidly.
Filled coastlines take considerably more time to generate (because map limits
are not necessarily rectangular, clipping must be accomplished in m-files).

## - Topography/Bathymetry options

M\_Map can access a 1-degree resolution global elevation database
(actually, this database is is included in the Matlab distribution,
used by `$MATLAB/toolbox/matlab/demos/earthmap.m`). A
contour map of elevations at default levels can be drawn using

```
m_elev;
```

Different levels can also be specified:

```
m_elev('contour',LEVELS, optional contour arguments);
```

For example, if you want all the contours to be dark blue, use:

```
m_elev('contour',LEVELS,'edgecolor','b');
```

Filled contours are also possible:

```
m_elev('contourf',LEVELS, optional contourf arguments);
```

Finally, if you want to simply extract the elevation data for your own
purposes,

```
[Z,LONG,LAT]=m_elev([LONG_MIN LONG_MAX LAT_MIN LAT_MAX]);
```

returns rectangular matrices for depths `Z` at locations `LONG,LAT`.

---

## 4. Customizing axes

### - Grid lines and labels

In order to get the perfect grid, you may want to
experiment with different grid options. Two functions are useful here,
`m_grid` which draws a grid, and `m_ungrid` which erases the current grid
(but leaves all coastlines and user-specified data alone). Try

```
  m_proj('Lambert');
  m_coast;
  m_grid;
```

to get a Lambert conic projection of North America.
Now try

```
  m_ungrid
```

The coastline is still there, but the grid has
disappeared and the axes shows raw X/Y projection coordinates. Now, try
this:

```
  m_grid('xtick',10,'tickdir','out','yaxislocation','right','fontsize',7);
```

The various options that can be changed include:

```
    'box',( 'on' | 'off' | 'fancy' )
```

which specifies whether or not an outline box is drawn.
Three types of outline boxes are available: `'on'`, the
default, is a a simple line. Two types of fancy outline boxes are
available. If `'tickdir'` is `'in'`, then
alternating black and white patches are made (see example 2). If `'tickdir'` is
set to `'out'`, then a more complex line pattern is drawn
(see example 6). Fancy boxes are in
general only available for maps bounded by lat/long limits (i.e. not
for azimuthal projections), but if this option is chosen
inappropriately a warning message is issued.

The number/location of ticks on the longitude grid is given by

```
   'xtick',( num | [value1 value2 ...])
```

If a single
number is specified, grid lines/values are drawn for approximately that
number of equally-spaced locations (the number is only approximate
because the M\_GRID attempts to find "nice" intervals, i.e. it rounds to
even increments). Exact locations can be specified by using a vector of
location values. There is an analogous  `'ytick'` 
property.

Special labels can be specified using

```
   'xticklabels',[label1;label2 ...]
```

Labels can either be numerical values
(which are then formatted by M\_GRID), or string values which are used
without change. There is an analogous  `'yticklabels'` 
property.

Longitude labels are either middled onto the ends of their respective
grid lines (and drawn perpendicular to those lines), or are drawn
extending outwards from the ends of those lines.

```
   'xlabeldir', ( 'middle' | 'end' )
```

There is an analogous `'ylabeldir'`  property.

The lengths of tickmarks (as a fraction of plot width) is specified using

```
   'ticklen',value
```

and their direction is set inwards or outwards with

```
   'tickdir',( 'in' | 'out' )
```

If `'box'`
is set to `'fancy'`, the `tickdir` specifies the form of the fancy
outline box (their is the `'in'` version and the `'out'` version).

Axis labels can be produced either in decimal degrees (`'dd'`) or degrees-minutes (`'dm'`, default). The
`'da'` option is an abbreviated degrees-minutes format without degree marks or the N/S/E/W letters appended:

```
  'tickstyle',( 'dd' | 'da' |  'dm' )
```

A number of other (obvious) properties can also be set:

```
'color',colorspec  
'gridcolor',colorspec
'backgroundcolor',colorspec  
'linewidth', value  
'linestyle', ( linespec | 'none' )  
'fontsize',value  
'fontname',name
```

Finally,

```
  'xaxisLocation',( 'bottom' | 'middle' | 'top' )
```

specifies where the X-axis will be drawn, either at the bottom
(southermost) end, at the top (northernmost) end, or in the middle (note - for southern hemisphere maps, especially
those containing the south pole, setting this to `'top'` is recommended), and

```
   'yaxisLocation',( 'left' | 'middle' | 'right' )
```

specifies where the Y-axis will be drawn, either at the left
(westernmostmost) end, at the right (easternmost) end, or in the
middle.

Note: if you are using the UTM projection, and you want to have a grid with eastings and northings, call
`m_utmgrid` AFTER calling `m_grid`. This will draw a UTM grid, independent of the lat/long grid provided by a call
to `m_grid`. Again, you can customize many aspects of the grid by setting appropriate properties.

### - Titles and x/ylabels

Titles and x/ylabels can be added to maps using the `title`
and `x/ylabel` functions in the usual
way (this is a change from v1.0 in which the `'visible'` property had to
be explicitly set to `'on'`; this is now done within `m_grid`).

### - Legend Boxes

A legend box can be added to the map using `m_legend`.
Only some of the functionality of `legend` is currently
included. The legend box can be dragged and dropped using the mouse
button.

### - Scale Bars

A scale bar can be added to the map using `m_ruler`. The
bar is drawn horizontally or vertically, and will create a 'nice' number of ticks
(although this can be changed with another calling parameter). The location is
specified in normalized coordinates (i.e. between 0 and 1) so you can adjust
placement on the map, see Examples 9 and 10.
It is probably best to call `m_ruler` AFTER calling `m_grid`
since `m_grid` resets the normalization.

WARNING - the scalebar is probably not useful for any global
(i.e. whole-world) or even a significant-part-of-the-globe
map, because the actual ground scale is often quite distorted in some
parts of the map, but I won't stop you using it. Caveat user!

### - North Arrow

An arrow pointing north is sometimes useful to have, and this can be
added using `m_northarrow`. There are a number of different types
of arrows available, see Examples 6, 10,
and 12. The arrow is located at a specified longitude/latitude,
which MAY be outside the map borders (i.e. it isn't clipped to the map boundaries), and is sized
in units of degrees of latitude. The arrow is drawn as a patch, so the usual patch properties
can be set. For example,

```
 m_northarrow(-150,0,40,'type',4,'linewi',.5);
```

draws the type 4 arrow, with thin outlines, as below:

---

## 5. Adding your own data

The purpose of M\_Map is to allow you to map your own data!
Once a suitable grid and (possibly) a coastline have been chosen, you
can add your own lines, text, or contour plots using built-in M\_Map
drawing functions which handle the conversion from longitude/latitude
coordinates to projection coordinates. These drawing functions are very
similar to the standard Matlab plotting functions, and are described in
the next section.

Sometimes you may want to convert between longitude/latitude and
projection coordinates without immediately plotting the data. This
might happen if you want to interactively select points using `ginput`,
or if you want to draw labels tied to a specific point on the screen
rather than a particular longitude/latitude. Projection conversion
routines are described in sections 5.5 and 5.6. Once raw longitude/latitude coordinates are
converted into projection coordinates, standard Matlab plotting
functions can be used.

Maps are drawn to fit within the boundaries of the plot axes. Thus
their scale is somewhat arbitrary. If you are interested in making a
map to
a given scale, e.g. 1:200000 or something like that, you can do so by
using the `m_scale` primitive, see 
section 2.6
. The data units are the projection coordinates, which are
distances
expressed as a fraction of earth radii. To get a map "distance" between
two points, use the Cartesian distance between the points in the
projection
coordinate system and multiply by your favourite value for the earth's
radius, usually around 6370 km (exception - the UTM projection uses
coordinates
of northing and easting in meters, so no conversion is necessary).

CAUTION: One problem that sometimes occurs is that data does not
appear on the plot due to ambiguities in longitude values. For example,
if plot longitude limits are [-180 180], a point with a longitude of,
say, 200,
may not appear in cylindrical and conic projections. THIS IS NOT A BUG.
Handling the clipping in "wrapped around" curves requires adding points
(rather than just moving them) and is therefore incompatible with
various
other requirements (such as keeping input and output matrices the same
size
in the conversion routines described below).

### - Drawing lines, text, arrows, patches, hatches, speckles and contours

For most purposes you do not need to know what the
projection coordinates actually are - you just want to plot something
at a specified longitude/latitude. Most of the time you when you want
to plot something on a map you want to do so by specifying
longitude/latitude coordinates, instead of the usual X/Y locations. To
do so in M\_Map, replace calls to  `plot, line, text, quiver,
patch, annotationcontour, and contourf`  with M\_Map equivalents that
recognize longitude/latitude coordinates by prepending "m\_" to the
function name. For example,

```
  m_plot(LONG,LAT,...line properties)      % draw a line on a map (erase current plot) 
  m_line(LONG,LAT,...line properties)      % draw a line on a map 
  m_text(LONG,LAT,'string')                % Text   
  m_quiver(LONG,LAT,U,V,S)                 % A quiver plot 
  m_patch(LONG,LAT,..patch properties)     % Patches.  
  m_annotation('line',LON,LAT)             % Annotation
```

Each of these functions will handle the coordinate
conversion internally, and will return a vector of handles to the
graphic objects if desired. The only difference between these functions
and the
standard Matlab functions is that the first two arguments MUST be
longitude
and latitude.

One caveat applies to `m_patch`. For
compatibility reasons this uses the same code that applies to coastline
filling. Coastlines come either as either "islands" or "lakes", and
M\_Map keeps track of the difference by assuming curves are oriented so
that the filled area ("land") is always on the right as we go around
the curve. This is slightly different than the convention used in `patch`
which always fills the inside. Keeping track of this difference is
relatively straightforward in a Cartesian system, but not so easy in
spherical coordinates. In the absence of other information `m_patch`
tries to do the right thing, but (especially when the patch intersects
a map boundary) it can get confused. If a patch isn't filling
correctly, try reversing the order of points using `flipud`
or `fliplr`.

Data gridded in longitude and latitude can also
be contoured:

```
  m_contour(LONG,LAT,VALUES)
  m_contourf(LONG,LAT,VALUES)
```

Again, these functions will return handles to graphics
objects, allowing (for example) the drawing of labelled contours:

```
  [cs,h]=m_contour(LONG,LAT,VALUES)
  clabel(cs,h,'fontsize',6);
```

Fancy arrows (i.e. with width, head shape, and
colour specifications) can be generated using  `m_vec.m` .
See the on-line help for more details about the use of `m_vec`.

You can also get hatched areas by calling `m_hatch`:

```
  m_hatch('single',LONG,LAT,...hatch properties)    % Interior Single Hatches. 
  m_hatch('cross',LONG,LAT,...hatch properties)     % Interior Crossed Hatches.
```

Note that this call does not generate the edge lines (an additional `m_line`
is required for this. In addition, we can speckle the inside edges of
patches using:

```
  m_hatch('speckle',LONG,LAT,...speckle properties)  % Speckled edges.
```

See the on-line help and/or Example
12 for more details about using `m_hatch`.

### - Drawing images and pcolor

Gridded data in matlab can be handled with either a) `pcolor`, usually for small grids, whose vertices are
specified in matrices the same size as that of the data, and where you might want to shade across each
grid cell, or b) `image`, often for much larger pixellated datasets where each value will be mapped to a colour in
rectangular cells all of which are the same size. `pcolor` is often used for datasets that one might
also handle with `contour` or `contourf`, while `image` is used for
photographs and the like.

1. In M\_Map, if you have a georeferenced image in lat/long coordinates (i.e.
   each data row is along a line of constant latitude, each column a line
   of equal longitude), then you can use `m_image`. That is,

   ```
   m_image(LON,LAT,DATA)
   ```

   will accept data in which `LON` and `LAT` are either two-element vectors
   which give the coordinates of the first and last row or column, or are vectors the same size as the number of
   rows or columns of `DATA` (this is a little more flexible than `image` since it
   means that rows can be unequally spaced in longitude). The data matrix will be regridded into map coordinates and
   displayed using `image`. Generally this will be a good idea only when the data pixels are
   already too small to see (see this example). Note that `DATA`
   can either by an NxM double precision floating point matrix (say, a bathymetry database), or an NxMx3 uint8 "truecolor"
   image (say, one derived from optical remote sensing).

   It is also sometimes useful to use `m_image` solely to transform the data in an NxM array,
   following it with a `m_shadedrelief` call to actually display the data

   ```
   [IM,X,Y]=m_image(LON,LAT,DATA);
   m_shadedrelief(X,Y,IM,'coords','map')
   ```

   Note that you should set the figure background colour (e.g., `set(gcf,'color','w')`)
   before calling `m_image` because pixels that
   appear outside the map boundaries are set to that background colour - see This example
2. If your georeferenced image is in lat/long coordinates, but all points in a row do NOT have the latitude,
   then use `m_pcolor` with `shading
   flat`. This is reasonably satisfactory (although it can be slow for
   large images), but you SHOULD offset your coordinates by
   one-half of the pixel spacing. This is because of the different
   behaviors of `pcolor` and `image` when given the same
   data. (see this example).

   Note: you must be careful with `m_pcolor` near map boundaries. Ideally
   one would want data to extend up to (but not across) a map boundary
   (i.e. polygons are clipped). However, due to the way in which matlab
   handles surfaces this is not easily done. Instead - unless you are
   using a simple cylindrical or conic projection - you will probably get
   a ragged edge for the coloured surface.

   Also, be aware that `image` will center the (i,j) pixel at the location of
   the (i,j)th entry of the X/Y matrices,as long as these matrices are regular in their spacing. If they
   are irregular, then pixels are spaced evenly between the 1st and last entries in the matrix. However
   `p_color` with `shading flat` will draw a panel between
   the (i,j),(i+1,j),(i+1,j+1),(i,j+1) coordinates of the X/Y matrices
   with a color corresponding to the data value at (i,j). Thus everything
   will appear shifted by one half a pixel spacing. This can be avoided with `shading interp`
   but then the computational time to create an image is greatly increased.- If your figure has already been placed in some projection, and
     if you know the exact parameters of that projection, you can probably
     use a straight `image` call and then overplot an M\_Map map. For example,
     polar satellite images are often given on an equally-spaced grid in a polar stereographic projection.
     In this case you should use m\_ll2xy to get the screen coordinates of
     the image corners, then use those points in an `image()` call before
     overplotting your data. See in particular  this example.

     HINT - check to see that coastlines overplot in the right place to make sure
     this is working correctly.

### - Drawing shaded relief maps

Shaded relief is a visualization feature used a lot by geologists and geophysicists, but generally not
by oceanographers (although perhaps it should be). The general idea is that instead of just contouring
a map (with different heights shown as different colours), as in the left example below, you additionally
shade the surface as if it was a 3D object, lit
from a single direction, say, (as a default) from the upper left corner of the map, as in the right example below.
Slopes facing towards the upper left
corner are brighter, and those facing away are darker.

Shaded relief thus lets you visually represent both the low-wavenumber variation (blobs of different colours), as well as
the high-wavenumber variation in an image (since slopes are a derivative, they amplify high-wavenumber information).

in M\_MAP, `m_shadedrelief` is used to construct such a map. However, it is generally only useful for
large scale maps covering a small area. This is because it is (essentially) a drop-in replacement for `image` showing
a true-colour image (like a photograph) and hence can only be used when all the pixels are the same size (this means that all
points are on a GRID in screen coordinates, and the spacing between points is always EQUAL), and the map axes limits
are a RECTANGLE. That is:

1. You should call `m_shadedrelief` only AFTER calls to `colormap` and `caxis`, because these are
   needed to determine the shading in the true-colour image.
2. `m_shadedrelief` most straightforward use is as a backdrop to maps with
   a rectangular outline box - either a cylindrical projection, or some
   other projection with `m_proj(...'rectbox','on')`.
   The simplest way of not running into problems is
   1. if your elevation data is in LAT/LON coords, use `m_proj('equidistant cylindrical',...)`
   2. if your elevation data is in UTM coords (meters E/N), use `m_proj('utm',...)`
3. Alternatively, you can use `m_shadedrelief` AFTER calling `m_image`
   (see Section 5.2); this can be done with ANY projection or outline box.

`m_shadedrelief` accepts several options. The essence of the mapping is that slopes are calculated relative to a light source,
and then a shading function is applied:

```
       Shading = clipval*tanh(slope/gradient)
```

where the shading increases with slope angle, up until a limiting value of about `gradient` degrees, after which
shading saturates. The saturated amount of shading is set by `clipval` which is between 0 and 1, where 0 means no shading
correction and 1 means shading to white or black.

The direction of the lighting can also be changed, but the default upper left source direction seems to be an accepted standard.

Finally, the slope calculation only works correctly if we know how to interpret x/y changes with Z changes. For UTM coordinate
data, all 3 would be in meters. However, a lot of high-resolution data is provided in lat/long coordinates, and hence the x/y directions would have to be
properly scaled. FInally, pixel locations may be in map coordinates.
The `coords` option can let you specify which of the cases is in use.

```
        'coo<rds>', ( 'g<eographic>'  |  'z'  |  'm<ap>' )
```

For more information, see  this example,  this one,
or  this one.

### - Drawing tracklines

It is sometimes useful to
annotate lines representing the time-varying location of a ship, aircraft, or satellite with time
and date annotations. This can be done using  `m_track`:

```
  m_proj('UTM','long',[-72 -68],'lat',[40 44]);
  m_gshhs_i('color','k');
  m_grid('box','fancy','tickdir','out');
  
  % fake up a trackline
  lons=[-71:.1:-67];
  lats=60*cos((lons+115)*pi/180);
  dates=datenum(1997,10,23,15,1:41,zeros(1,41));
  
  m_track(lons,lats,dates,'ticks',0,'times',4,'dates',8,...
               'clip','off','color','r','orient','upright');
```

 

See the on-line help for more details about the use of `m_track`,
and the different options for setting fontsize, tick spacing, date
formats, etc.

While fiddling with the various parameters, it
is often handy to be able to erase the plotted tracks without erasing the
coastline and grid. This can be done using

```
  m_ungrid track
```

or

```
  m_ungrid('track')
```

### - Drawing range rings and geodesics

One nifty thing that is sometimes useful is the
ability to draw circles at a given range or ranges from a specific
location. This can be done using  `m_range_ring`, which has
3 required calling parameters: LONG, LAT, RANGE, followed by any number
of (optional) line specification property/value pairs. Example 11 illustrates how to use `m_range_ring`.

If you want to plot circular geodesics (i.e. curves which are
perpedicular to the range rings at all ranges), `m_lldist` can find both
distances and points along the geodesics between points. Example
13 illustrates how to use `m_lldist`.

If you care about the difference between great circle and
ellipsoidal geodesics (a very very small proportion of users I would
bet) then `m_fdist` (which computes the position at a given
range/bearing from another), `m_idist` (distance and
bearings between points), and `m_geodesic` (points along
the geodesic) can be used with a variety of (user-specified) ellipses.
The calling sequence for these is different than for `m_lldist`
for historical reasons.

### - Drawing tidal ellipses and wind roses

For oceanographers, the cyclic variation of tidal currents are shown on maps in the form of tidal ellipses.
Use `m_ellipse` to draw these ellipses, passing arguments giving the
ellipse location, lengths of semi-major and semi-minor axes, ellipse inclination and (optionally) the Greenwich
phase. In order to interpret these ellipses, imagine that the tip of a velocity vector circles around this
ellipse at a given frequency, in either the CW or CCW direction. The Greenwich phase provides the location of
the velocity vector when astronomical forcing is at its lowest at the Greenwich meridian.

Wind roses, on the other hand, are a way of displaying the speed and direction statistics of winds (or currents)
irrespective of frequency. Bars extend in
the direction of the currents, and the length of coloured segments along the bar represent the relative frequency of
observations of a particular speed range in that direction. A background set of rings are "plot axes".

The function `m_windrose` can be used to display such a rose on a map. Typically one would only
use this display if there were multiple stations for which statistics are required; single stations (or a small
number of stations) are usually shown on their own, in a slightly larger format. In the figure below, winter winds are seen
to be generally southeasterly, with northwesterlies occurring less frequently along most of the Strait of Georgia, Canada, except at the SE end where the
dominant directions are easterly or southerly. The background circle
and rings at 2, 4, and 6% at each location for which we have wind statistics are made slightly transparent
so that the coastline is visible through them in the default call. Here they
have been made fully opaque. Code to make this plot is provided in Example
19.

 

### - Converting longitude/latitude to projection coordinates

If you want to use projection coordinates (perhaps
you want to compute map areas, or distances, or you want to make a legend
in the upper left corner), the following command converts
longitude/latitude coordinates to projection coordinates.

```
  [X,Y]=m_ll2xy(LONG,LAT, ...optional clipping arguments )
```

where LONG, LAT, X, and Y are matrices of the same
size. Projection coordinates are equal to true distances near the
center of the map, and are expressed as fractions of an earth radius.
To get a distance, multiply by the radius of the earth (about 6370km).
The exception is the UTM projection which provides coordinates of
northing and easting in meters.

The possible clipping arguments are

```
   'clip','on'
```

This is the default. Columns of LONG and LAT are
assumed to form lines, and these are clipped to the map limits. The
first point outside the map is therefore moved to the map edge, and all other
points are converted the NaN.

```
   'clip','off'
```

No clipping is performed. This is sometimes useful
for debugging purposes.

```
  'clip','point'
```

Points are tested against the map limits. Those
outside the limits are converted to NaN, those inside are converted to
projection coordinates. No points are moved. This option is useful for
point data
(such as station locations).

```
   'clip','patch'
```

Points are tested against the map limits. Those
outside the limits changed into a point exactly on the limits. Those
inside are converted to projection coordinates. This option may be
useful when trying to draw patches, however it probably won't work
well.

### - Converting projection to longitude/latitude coordinates

Conversion from projection coordinates to
longitude/latitude is straightforward:

```
  [LONG,LAT]=m_xy2ll(X,Y)
```

There are no options.

### - Computing distances between points

Geodesic (great circle) distances on a spherical
earth can be computed between pairs of either geographic (long/lat) or map
(X/Y) coordinates using the functions `m_lldist` and `m_xydist`.
For example,

```
  DIST=m_lldist([20 30],[44 45])
```

computes the distance from 20E, 44N to 30E, 45N.
Alternatively, if you want to compute the distance between two points
selected by the mouse:

```
  [X,Y]=ginput(2);
  DIST=m_xydist(X,Y)
```

will return that distance. Because of the
inaccuracies implicit in a spherical earth approximation the true geodesic distances
may differ by 1% or so from the computed distances.

If you want greater accuracy, then you must calculate geodesics on an
ellipsoidal earth. There is a very accurate numerical algorithm for
doing so ( Vincenty's
algorithm), which is implemented in the functions
 `m_idist`, `m_fdist`, and  `m_geodesic`. For example,

```
 [distance,a12,a21] = m_idist(lon1,lat1,lon2,lat2,spheroid)
```

computes the `distance`  in meters between two points `(lon1,lat1)`
and `(lon2,lat2)` on the specified spheroid (`'wgs84'` is the
default, for other options see the code or use on of the options shown
by `m_proj('get','utm')`). Forward and reverse azimuths (bearings for the rest of us) `a12`
and `a21` in degrees are also computed.

`m_fdist` is used to get the location of a point at a given bearing and
distance from a specified point. Example 6 shows the use of these functions
to find a midpoint of a drifter track.

Finally, if you want to plot a geodesic on a map, then  `m_geodesic`
can be used to generate a vector of points along the elliptical geodesic between two specified
points. If you ever find yourself needing this, I'd be interested in knowing about it!

### - Annotation and Mouse Input

Adding labels and boxes to your map may be simpler with `m_annotation`, which lets you input arrow and
text location using
latitude/longitude coordinates and otherwise passes arguments through to the built-in MATLAB function `annotation.m`.
Note that this function is fragile with respect to window resizing. Since the
`annotation` coordinates are normalized to the window size, and the map's boundaries may move with respect to the window edges
if you change the aspect ratio of the plot window (by, e.g., resizing the window with the mouse), it is best to either

- refrain from changing the window size between the `m_annotation` call and a subsequent `print` call, or
- carry out operations in the order:

  ```
  m_proj('miller');   % Map first
  m_coast;
  m_grid;
  ...
  orient tall;        % Fix the orientation, and...
  wysiwyg;            % make the screen version match the (future) printed version
  m_annotation('textarrow',[-140 -122.6],[20 49.35],'string','Vancouver')
  ...
  print
  ```

Note that `m_ginput` (which works just like a better `ginput`, except that it returns long/lat coordinates) might simplify finding the correct long/lat for your annotation.

### - Colour and Colourmaps

 

The use of colour to show bathymetry/topography on a map, or values of other parameters displayed on a map,
is often critical, and `m_colmap` contains
a number of useful colourmaps to do this. These include several maps with good perceptual qualities, as well as a number which
`look good' on geographic maps. You can generate the plot above, which gives examples of different calls, using:

```
m_colmap demo
```

What does it mean to have 'good perceptual qualities'? Nowadays most people know that
Matlab's `jet` colormap has a number of shortcoming. In particular it is NOT PERCEPTUALLY UNIFORM - we
can see fine details around the yellow colour band, but the wide range of blues are hard to separate.

More modern colourmaps are designed to overcome this problem, by designing a
gradation in colour-space which looks uniform
to the human eye. One school of thought does away
with colours altogether, in favour of what is essentially a PERCEPTUALLY UNIFORM single-colour scale that goes from
dark to light. Examples
b and h above are this kind of colormap (adapted from the ColorBrewer), but so is Matlab's `parula` (more or less), `hot` and
 `gray`.
These kinds of colourmap let us distinguish 2 levels: 'high' (light) and 'low' (dark).

Such simple colourmaps are not helpful for data that has a +/- sign (e.g., current velocities), and so for this kind of
data a DIVERGING
colormap (like example d above) is often used. Diverging colourmaps let us distinguish 3 levels: 'negative','zero', and
'positive'. They are also good for 'below-average', 'average', and 'above-average'.

What if we want more gradations? There is no reason why we can't have the multiple colours of a JET-like colourmap, which makes
it much easier to identify several levels within an image, once we design in a perceptually uniform luminance scale.
Thus we can create a PERCEPTUALLY UNIFORM JET colourmap. Example a above shows a smooth version (taken from
the 
CET Perceptually Uniform Colour Maps toolbox), which shows about 5 different levels, but examples j and k also show versions with
an arbitrary (but small) number of distinct colours which are all, to the eye, about equally different to one another.

Example j is useful for false-colour satellite imagery, where values are contaminated by noise. The idea of the gradual
blurring at boundaries between colours is that it will de-emphasize speckles that would otherwise arise.

Example k is useful for numerical models, or other data that is already smooth, so quantizing to a small number of levels
will not be a problem. The sharp boundaries will then act a little like contours on the final image.

Finally, `m_colmap` includes several specialized colormaps (examples c, f, i, and k) designed to show land; these are based on
the ETOPO1 land colormap from the
Cartographic Palette Archive.

### - Colourbars with Contourmaps

Matlab has the `colorbar` command which can be used to add a scale to smoothly-plotted data (see Satellite
examples 1, 2 or
4) It can also be useful if you try to simulate contours
used a stepped colourmap as in Example 14).
But if you are actually showing filled contours, the continuous set of colours in the default colormap
does not correspond with the small number of
discrete colours shown in the contourmap. This may be important in figures like Example 15,
7 or 8.

For these latter situations use `m_contfbar`. Typical usage is

```
   m_contourf(LON,LAT,DATA,LEVELS);
   m_contfbar(Xloc,Yloc,DATA,LEVELS);
```

or

```
   [CS,CH]=m_contourf(LON,LAT,DATA,LEVELS);
   m_contfbar(Xloc,Yloc,CS,CH);
```

or other calls that involve `contourf` like

```
   [CS,CH]=m_etopo2('contourf',LEVELS);
   m_contfbar(Xloc,Yloc,CS,CH);
```

where `Xloc=[X1,X2]` and `Yloc=Y` for a horizontal scale bar at normalized height `Y`, with
sides at normalized x-locations `X1` and `X2` (normalized means between 0 and 1). For a vertical scale bar,
`Xloc=X1` and `Yloc=[Y1,Y2]`. By using the same data and levels information, the scale bar shows the
same levels as that of the filled contour image itself.

`m_contfbar` can also be used with `M_shadedrelief`:

```
   caxis([CMIN CMAX])
   colormap(map)
   m_shadedrelief(LON,LAT,DATA)
   m_contfbar(Xloc,Yloc,DATA,LEVELS);
```

where you are free to choose the `LEVELS` as you wish.

There are 4 additional parameter/value pairs that can be used to modify the appearance of the scale bar. First,
the width of the bar can be set to 0.03 of the full axis width using:

```
   'axfrac',.03
```

and triangular endpieces (signifying data outside the region of the colour scale) can be added with

```
   'endpiece',[ 'yes' | 'no']
```

If you don't like having a black edging line between the colours, choose the `'none'` option:

```
   'edgecolor',[colorspec | 'none']
```

and finally, it is possible to set the `LEVELS` vector to have levels that are outside the range
of values in `DATA`. Do you want the scale bar show these levels, or to show only the
levels that match those in the range of `DATA`? Specify with:

```
   'levels',['set' (default) | 'match']
```

---

## 6. More complex maps

For ideas on how to make more complex maps, see the Examples. Some of these maps are also
included
in the function `m_demo`.

---

## 7. Removing features from a map

Once a given map includes several elements a certain amount of
fiddling is usually necessary to satisfy the natural human urge to give
the image a certain aesthetic quality. If the image includes
complicated coastlines which take a long time to draw (e.g. those
discussed below) than clearing the figure and redrawing soon becomes
tedious. The `m_ungrid` command introduced above can be
used to selectively remove parts of the
figure. For example:

```
m_proj('lambert','long',[-160 -40],'lat',[30 80]);
m_coast;
m_range_ring(-123,49,[1e3:1e3:10e3],'color','r');
```

draws range rings at 1000km increments from my office. But I am
unsatisfied with this, and want to redraw using only 200km increments.
I can remove the effects of `m_range_ring` and redraw
using:

```
m_ungrid range_ring
m_range_ring(-123,49,[200:200:2000],'color','r');
```

In general the results of `m_ANYTHING` can be deleted
by calling `m_ungrid ANYTHING`. `m_ungrid` can recognize and delete specific elements
by searching the `'tag'` property of all plot elements,
which is set by M\_Map routines.

The `'tag'` property is also useful if you want to make your
own modifications.
For example, say you want to REMOVE every 2nd xticklabel (you like the extra
lines in a grid, but not that many labels). You can do this with:

```
handles=findobj('gca,'tag','m_grid_xticklabel');
delete(handles(2:2:end));
```

---

## 8. Adding your own coastlines

If you are interested in a particular area and want a
higher-resolution coastline than that used by `m_coast`,
the best procedure is to get one of the high-resolution databases I describe below. If this
doesn't work, first I give are some hints on how to deal with your own coastlines.

### - Reading and Handling coastline data

If you have data is stored in 2 columns (longitudes then latitudes, with line segments separated by a row
of NaNs) in a file
named "coast.dat", you can plot it (as lines) using the following:

```
load coast.dat
m_line(coast(:,1),coast(:,2));
```

Filled coastlines will require more work. First, if the coastline is in
a number of discrete segments, you have to join them all together to
make complete "islands" and "lakes". If you are lucky, (i.e. no lakes or anything else),
you *may*
achieve success with

```
load coast.dat
[X,Y]=m_ll2xy(coast(:,1),coast(:,2),'clip','patch');
k=[find(isnan(X(:,1)))];
for i=1:length(k)-1,
     x=coast([k(i)+1:(k(i+1)-1) k(i)+1],1);
     y=coast([k(i)+1:(k(i+1)-1) k(i)+1],2);
     patch(x,y,'r');
 end;
```

and then try replacing `patch` with `m_patch`.

If this does not work (e.g., because your coastline includes "lakes"),
read the comments in `private/mu_coast`,
orient the curves in the desired fashion, and use `m_usercoast`
to load your own data.

### - ESRI Shapefiles

A de facto standard for the interchange of vector data are ESRI shapefiles. A dataset
comes in (at minimum) 3 files, each with the same root name but
with `.dbf`, `.shp`, and `.shx` extensions. Files
can contain point, line or polygon information, as well as other fields in a self-describing
way. For more information see  this
description .

Many (all?) shapefiles can be read into Matlab using `m_shaperead`, which returns
a data structure containing the information in the files. However,
figuring out what to do with the contents requires you to examine the contents of the
data structure.

You can usually at least create a simple plot of the data stored in files
`datafile.shp`, `datafile.shx` and `datafile.dbf`
using

```
M=m_shaperead('datafile'); 
clf; 
for k=1:length(M.ncst), 
     line(M.ncst{k}(:,1),M.ncst{k}(:,2)); 
end;
```

If the data is already in lat/long coordinates, change the `line` to `m_line`.

### - Projection Conversions

Sometimes coastline data is already provided in the coordinates of some projection.
Usually you will want to convert this data back to
lat/long by a) calling `m_proj` with the specifications of that projection,
and b) calling `m_xy2ll` with the data you read in.

For data in UTM coordinates, this is particularly easy. For example, for data in an area around
Vancouver, Canada, we are told the ellipse parameters (grs80) for a particular dataset, so:

```
m_proj('utm','ellipse','grs80','lat',[49+15.7/60 49+21/60],...
        'long',[-123-15/60 -123-3/60]);      
[LONG,LAT]=m_xy2ll(eastings,northings);
```

In other cases, first find the projection information which is usually provided somewhere - in a README, or (perhaps)
a `.prj` file (this is especially true for shapefile information).
Examine the `.prj` text file.
As an example, the Cascadia DEM
contains data in coordinates
defined by a file `cascadia.prj`:

```
--------------cascadia.prj--------------
Projection    LAMBERT                                                           
Zunits        NO                                                                
Units         METERS                                                            
Spheroid      CLARKE1866                                                        
Xshift        0.0000000000                                                      
Yshift        0.0000000000                                                      
Parameters                                                                      
 41 30  0.000 /* 1st standard parallel                                          
 50 30  0.000 /* 2nd standard parallel                                          
-124 30  0.000 /* central meridian                                              
 38  0  0.000 /* latitude of projection's origin                                
0.00000 /* false easting (meters)                                       
0.00000 /* false northing (meters) 
---------------end of file----------------
```

so to convert from projection coordinates back to lat/long you would use:

```
m_proj('lambert','parallels',[41.5 50.5],'long',[-133 -116],...
             'lat',[39 53],'false',[-124.5 38],'ellipsoid','clrk66');
```

In another example, data from WA, USA is provided in a projection specified using:

```
-------------------beginning of .prj file----------
 PROJCS["NAD_1983_HARN_StatePlane_Washington_South_FIPS_4602_Feet",
 GEOGCS["GCS_North_American_1983_HARN",
 DATUM["D_North_American_1983_HARN",
 SPHEROID["GRS_1980",6378137.0,298.257222101]],
 PRIMEM["Greenwich",0.0],
 UNIT["Degree",0.0174532925199433]],
 PROJECTION["Lambert_Conformal_Conic"],
 PARAMETER["False_Easting",1640416.666666667],
 PARAMETER["False_Northing",0.0],
 PARAMETER["Central_Meridian",-120.5],
 PARAMETER["Standard_Parallel_1",45.83333333333334],
 PARAMETER["Standard_Parallel_2",47.33333333333334],
 PARAMETER["Latitude_Of_Origin",45.33333333333334],
 UNIT["Foot_US",0.3048006096012192]]
 -----------------end of file-----------------------
```

and we convert data back using:

```
 m_proj('lambert conformal','ellipsoid','grs80','par',[45.83333333333334 47.33333333333334],...
        'clong',-120.5,'false',[-120.5 45.33333333333334]);

  [LONG,LAT]=m_xy2ll( (X-1640416.666666667)*0.3048006096012192,Y*0.3048006096012192);
```

### - Coastline Extractor

In the past one could get high-resolution data from  The
Coastline
Extractor, but as of 2015 this web site has been decommissioned.

### - DCW political boundaries

##### As of 2011 the DCW web site has been decommissioned. The following information is retained for historical reasons only. New users see the next section on Natural Earth.

Files containing political boundaries for various countries and
US states can be downloaded from 
http://www.maproom.psu.edu/dcw/. Select an area and choose the
"download points" option (rather than "download data"). Once downloaded to your
machine use `m_plotbndry` to access and plot the desired boundary.
For example, if you downloaded various US states into a subdirectory
"states:,

```
  m_plotbndry('states/arizona','color,'r')
```

would plot arizona on the current map.

### Natural Earth Political Boundaries

Political Boundary info is available in shapefile format from
Natural Earth. Download the shapefiles for areas
you are interested in and use `m_shaperead` as described above.

### - GSHHS(G) high-resolution coastline database

When drawing maps there is always a tradeoff between the
execution time of the generating program and the resolution of the resulting map.
Included in M\_Map is a 1/4 degree coastline database which can be used
to generate very fast maps, with adequate resolution for many purposes.

However, it is often desirable to be able to make detailed maps
of limited geographic areas. For this purpose a higher-resolution
coastline database is necessary. I have not included such a database in M\_Map
because it would greatly increase the size of the package. However, I have
included m-files to access and use a popular high-resolution database called  GSHHS (as of 2016
now called GSHHG).

As distributed, GSHHG consists of a hierarchical set of
databases at different resolutions. The lowest or "crude" resolution is not as
good as the M\_Map database, although it contains many more inland lakes. The
"high" resolution consists of points about 200m apart. There is also an
even finer "full" resolution. You can install part or all of the database
(depending on how much disk space you have available). The "full"
resolution occupies 90Mb of disk space, and successively coarser
resolutions are smaller by about 1/4. Thus "high" resolution occupies
21Mb, "intermediate" uses 6Mb, and "low" uses 1.1Mb (one reason for not
always using "high" resolution is that the entire 90Mb database must be read and processed each call,
which may take some time).

#### - Installing GSHHS

1. Go to 
   https://www.ngdc.noaa.gov/mgg/shorelines/data/gshhs/.
2. Get `gshhg-bin-2.3.6.zip` (as of late 2017) and uncompress any or all of the files there - `gshhs_*.b, wdb_borders_*.b, and wdb_rivers_*.b` for coastlines, borders,
   and rivers respectively,
   in a convenient directory. One useful place is in `m_map/data`.
   GSHHS data format has changed between v1.2 and 1.3, and again for v2.0, but m\_map
   should be able to figure this out.
3. If the database files are not in subdirectory `m_map/data`, you must edit the `FILNAME`
   setting in `m_gshhs.m` to point to the appropriate files.

#### - Using GSHHS effectively

The simplest calling mechanism is identical
to that for  `m_coast` (Section 3). For
example, to draw a gray-filled high-resolution coastline, you just need

```
  m_gshhs_h('patch',[.5 .5 .5]);
```

However, execution times may be very, very long, as the
entire database must be searched and processed. I would not recommend
trying to draw world maps with the intermediate or high-resolution
coastlines! There are two ways to speed this up. The first is to
use a lower-resolution database, with fewer points. The second is
useful if you are going to
be repeatedly drawing a map (because, for example, it's the base figure
for your work). In this case I recommend that you save an intermediate
processed (generally smaller) file as follows:

```
  m_proj ...  % set up projection parameters
  
  % This command does not draw anything - it merely processes the 
  % high-resolution database using the current projection parameters 
  % to generate a smaller coastline file called "gumby"
  
  m_gshhs_h('save','gumby');
  
  % Now we can draw a few maps of the same area much more quickly
  
  figure(1);
  m_usercoast('gumby','patch','r');
  m_grid;
  
  figure(2);
  m_usercoast('gumby','linewidth',2,'color','b');
  m_grid('tickdir','out','yaxisloc','left');
  
  etc.
```

Note that there another way of getting this info, as well as river
and border info, is with the `m_gshhs.m` function,
whose first argument can be used to specify different options.

```
m_gshhs('lc','patch','r');  % Low resolution filled coastline
m_gshhs('fb1');             % Full resolution national borders
m_gshhs('ir');              % Intermediate resolution rivers
```

---

## 9. Adding your own topography/bathymetry

A number of global and regional topography databases are
available at  NCAR . Several are available
for free from  their ftp
site.

As long as the data is stored in a mat-file as a rectangular
matrix in longitude/latitude, then `m_contour` or `m_contourf`
can be used to plot that data.

### Sandwell and Smith Bathymetry

A recent new bathymetry with approximately 1km resolution in
lower latitude areas is being used by many people. This dataset is
described
at 
https://topex.ucsd.edu/marine\_topo/   and is available as a
134Mb binary file at 
ftp://topex.ucsd.edu/pub/global\_topo\_2min/  (get the file
topo\_X.Y.img where X.Y is the version number) - note as of 2017 this is now
a 729Mb binary at 
ftp://topex.ucsd.edu/pub/global\_topo\_1min/. I have
included an m-file (`mygrid_sand2.m`)
which can extract portions of the data (you will have to modify path
names within the code). Once this database (and the m-file) is
installed on your computer, you can use it in M\_Map very easily. A typical usage is
as follows:

```
  % Extract data
  [elevations,lat,lon]=mygrid_sand([long_west long_east lat_south lat_north ]);
  % Use in M_Map command
  m_contour(lon,lat,elevations);
```

For some projections, you must make sure that the 'lon' values returned
by `mygrid_sand2.m` fall within the range used in this
projection (i.e. you may have to add/subtract 360). This seems to
happen all the time for areas in the west (i.e. negative longitudes),
if you forget this you often end up with bewildering error messages
about empty vectors!

### - TerrainBase 5-minute global bathymetry/topography

THIS INFO IS KEPT FOR HISTORICAL REASONS - USE ETOPO1 (see below)

For many purposes the elevation database accessed by M\_Map
provides
adequate resolution. However, there are also many cases when more
detail
is desired. I have not included a higher-resolution database because it
would greatly increase the size of the package. However, v1.2 includes
m-files to access and plot a popular global 5-minute
bathymetry/topography database, after a few minutes of work.

This section provides instructions on how to download  TerrainBase, and
convert it from a 56Mb ASCII file to a 18Mb binary file using `m_tba2b.m`. It is then straightforward to access and plot
bathymetry from this file using  `m_tbase.m`, which is in
every way functionally identical to `m_elev` (see Section 3.2).

TerrainBase is also available on CDrom, and is also commonly
stored
in netcdf (or other) binary format somewhere on many academic networks.
If you modify  `m_tbase.m`  to access data from one of
these sources, let me know!

How to install TerrainBase:

1. get and uncompress the tbase.Z file from https://dss.ucar.edu/datasets/ds759.2/
    into the m\_map directory.
2. Run  `m_tba2b('PATHNAME')`  to store the
   resulting 18Mb binary file as  `PATHNAME/tbase.int`.
3. Delete the original ASCII file `tbase`.
4. Edit the `PATHNAME` setting in `m_tbase`
   to point to the location of this file.

That's it! Test things out with this map of the western mediterranean:

```
m_proj('lambert','lon',[-10 20],'lat',[33 48]);
m_tbase('contourf');
m_grid('linestyle','none','tickdir','out','linewidth',3);
```

### - ETOPO 2 or 1-minute global bathymetry/topography

ETOPO is a useful database, but it has undergone a number of changes in the last few years. Since it seems
to be released primarily as a netCDF file now it is possible `m_etopo2` should be completely rewritten,
but instead I have just modified the "traditional" method of using the database from binary files. Read all of the points
below and then follow the instructions in (4):

1. (2004-2014 instructions: now obsolete), there is a corrected higher-resolution (2 minute) database  ETOPO2. Download
   https://rda.ucar.edu/dsszone/ds759.3/etopo2\_2006apr/etopo2\_2006apr.raw.gz (a gzipped binary),
   gunzip it into a 116Mb file, edit the `PATHNAME` setting in `m_etopo2`
   to point to the location of this file, and then use it in the same way as `m_tbase`
   and `m_elev`. UCAR requires users to register and the second link won't work without you doing
   this (go to first link and follow instructions).
2. (2014-2017 instructions: mostly obsolete) In 2014, it was pointed out to me that the above is obsolete. First, there is a corrected 2-minute
   ETOPO database -  ETOPO2v2  which you should
   be using instead. Now, ETOPO2v2 is a little more complicated, because it comes in 4 version - big-endian
   and little-endian, in both cell-centered and grid-centered versions.

   It doesn't particularly matter if you get big- or little-endian since you can modify the `fopen`
   line in `m_etopo2` to account for this. I recommend getting the grid-centered version, since
   it works "better" when you are contouring the elevations (it will be more likely to extend all the way up
   to the map edge without weird little 'gaps').

   In any case, download one of the zipped binaries, unzip it, and then edit 4 lines in `m_etopo2`
   to set the `PATHNAME`, the filename in the `fopen` line, as well as setting the
   last option to `'b'` or `'l'` for big-endian or little-endian formats. Then
   make sure the `grid` and `resolution` parameters are set appropriately. If you
   forget (or get them wrong), code may run but it won't give the right bathymetry!
3. If you want even higher resolution bathymetry, you can also use the 1-minute
   ETOPO1. This appears to come in
   two versions: grid or cell-referenced, both little-endian. Again, I recommend the grid-referenced
   version `etopo1_ice_g_i2.bin`. Modify the relevant lines in `m_etopo2` in the same way as for ETOPO2v2.
4. (2017-present instructions: use these) As of 2017, notice that the file you want for ETOPO1, `etopo1_ice_g_i2.bin` is not available
   as a link from that page - instead they just reference a netCDF and a geo-referenced tiff version. Also, you will see two different versions
   that handle differences between the true surface and the top of the permanent icepacks in Greenland and Antarctica. Fear not! If you
   click on one of those, you fall into a directory page - click PARENT DIRECTORY, then binary, and you
   get into the place you want! For example, 
   https://www.ngdc.noaa.gov/mgg/global/relief/ETOPO1/data/ice\_surface/grid\_registered/binary/ for the top-of-the-ice data.

   As in the above instructions, download the 
   zipped binary "etopo1\_ice\_g\_i2.zip", unzip it, and then edit 4 lines in `m_etopo2`
   to set the `PATHNAME`, the filename in the `fopen` line, as well as setting the
   last option to `'b'` or `'l'` for big-endian or little-endian formats (as of 2017 this file
   seems to be available in little-endian format only). Then
   make sure the `grid` and `resolution` parameters are set appropriately. If you
   forget (or get them wrong), code may run but it won't give the right bathymetry - try a quick map to test it!

---

## 10. M\_Map toolbox contents and description

1. Contents.m - toolbox contents
2. m\_demo.m - demonstrates a few maps.

User-callable functions

1. m\_proj.m - initializes projection
2. m\_coord - sets geomagnetic or geographic coordinate system
3. m\_grid.m - draws grids
4. m\_utmgrid.m - draws a UTM grid for UTM projection maps
5. m\_scale.m - forces map to a given scale
6. m\_ruler.m - draws a scale bar
7. m\_northarrow.m - draws a north arrow
8. m\_ungrid.m - erases map elements (if you want to change
   parameters)
9. m\_coast.m - draws a coastline
10. m\_elev.m - draws elevation data
11. m\_tbase.m - draws elevation data from high-resolution database
12. m\_etopo2.m - draws elevation data from (another) high-resolution database
13. m\_gshhs.m - draws coastline from GSHHS with specified resolution
14. m\_gshhs\_c.m - draws coastline from GSHHS crude database
15. m\_gshhs\_l.m - draws coastline from GSHHS low-resolution
    database
16. m\_gshhs\_i.m - draws coastline from GSHHS
    intermediate-resolution
    database
17. m\_gshhs\_h.m - draws coastline from GSHHS high-resolution
    database
18. m\_gshhs\_f.m - draws coastline from GSHHS full resolution
    database
19. m\_plotbndry.m - draws a political boundary from the DCW
20. m\_usercoast.m - draws a coastline using a user-specified
    subset database.- m\_shaperead.m - reads ESRI shapefiles
21. m\_plot.m - draws line data in map coords
22. m\_line.m - draws line data in map coords
23. m\_text.m - adds text data in map coords
24. m\_legend.m - Draw a legend box
25. m\_quiver.m - draws arrows for vector data
26. m\_contour.m - draws contour lines for gridded data
27. m\_contourf.m - draws filled contours
28. m\_patch.m - adds patch data in map coords
29. m\_pcolor.m - draws pcolor surface
30. m\_image.m - draws image data
31. m\_streamline.m - draws streamlines
32. m\_annotation.m- annotation lines/boxes/text
33. m\_ginput.m - gets points in a ginput-like way (only better)
34. m\_shadedrelief.m shaded relief mapping
  
35. m\_track.m - draws annotated tracklines
36. m\_hatch - hatched or speckled hatches
37. m\_range\_ring.m - draws range rings
38. m\_ellipse.m - draws tidal ellipses (most requested ocean feature!)
39. m\_windrose.m - draws wind rose diagrams at specified locations.
40. m\_ll2xy.m - converts from long/lat to map coordinates
41. m\_xy2ll.m - converts from map coordinates to long/lat
42. m\_geo2mag.m - converts from magnetic to geographic coords
43. m\_mag2geo.m - the reverse
  
44. m\_lldist.m - distance between long/lat points
45. m\_xydist.m - distance between map coordinate points
  
46. m\_fdist.m - location of point at given range/bearing along
    ellipsoidal earth
47. m\_idist.m - range/bearings between points on ellipsoidal earth
48. m\_geodesic.m - points on geodesics between given points on
    ellipsoidal earth
49. m\_tba2b.m - used in installing high-resolution elevation
    database.
50. m\_vec.m - fancy arrows
51. m\_windbarb.m - fancy arrows for meteorologists
  
52. m\_contfbar.m - draws colorbars for contourf plots
53. m\_colmap.m - useful perceptually uniform colourmaps
54. mygrid\_sand2.m - reads Sandwell and Smith bathymetry file
  
55. wysiwyg.m - Sets figure window to match size/aspect of printed output

Internal functions (not meant to be user-callable)

1. private/mp\_azim.m - azimuthal projections
2. private/mp\_cyl.m - cylindrical projections (equatorial)
3. private/mp\_conic.m - conic projections
4. private/mp\_tmerc.m - transverse cylindrical projections
5. private/mp\_utm.m - elliptical universal transverse cylindrical
   projections
6. private/mp\_omerc.m - oblique cylindrical projection
7. private/mu\_util.m - various utility routines
8. private/mu\_coast.m - routines to handle coastlines.
9. private/mc\_coords.m - coordinate conversion.
10. private/mc\_igrf.m - parameters for IGRF geomagnetic coord systems.
11. private/mc\_ellips.m - parameters of different ellipsoidal earth models
12. private/clabel.m - patched version of clabel (matlab v5.1
    version does not contain capabilities for different text properties).
13. private/m\_coasts.mat - coastline data
14. private/igrf13coeffs.txt - database for IGRF coordinate systems

HTML Documentation

1. map.html - documentation intro
2. mapug.html - users guide
3. various .png in doc/ - examples.

---

## 11. Known Problems and Bugs

1. **Lakes come out black!** If plotted data is coloured white, the `print` changes it to
   black in output figures. In order to avoid this, set the figure
   background to white, i.e.

   ```
       set(gcf,'color','white')
   ```
2. **Filled coastlines go weird**. If you try to use azimuthal projections covering nearly half the
   globe, or oblique mercator projections over large areas, sometimes coastline patching fails (this is due
   to rounding errors in the projection math fpor points very near the map boundary). To solve this you can either
   - Adjust the projection parameters (lat/lon limits, radius, etc.) VERY SLIGHTLY so that the problematic
     points are no longer as close to the map boundary, or
   - Use line coastlines instead of patches.
3. **Generally weird-looking stuff that happens when you use filled contours.**
   For some reason this has been a glory-hole for all kinds of weird bugs in MATLAB. Most
   of them relate somehow to the way in which the map background interacts with
   contourf patches, and how the 'renderer' (the internal matlab code that figures out what
   goes on top of what) works, or doesn't work. Unfortunately I can't think of way that
   works around the problem in
   all cases, but if you see something weird, try:

   ```
    set(findobj('tag','m_grid_color'),'facecolor','none') 
   ```

   after the

   ```
   m_grid
   ```

   call, or

   ```
    set(gcf,'renderer','opengl');
   ```

   (under Unix you may have to do this one on starting MATLAB)
4. **Things not appearing correctly in tiff output**. Pre 2014b Matlab
   uses ghostscript to covert from ps to many other formats. But their version
   has some problems. It may be better to print to postscript and do the
   conversion (say, to tiff) yourself.

## 12. OCTAVE Compatibility Issues

From their website: "GNU Octave is a high-level interpreted language,
primarily intended for numerical computations. [...] The Octave language is quite similar
to Matlab so that most programs are easily portable."

M\_Map currently runs under Octave...mostly. `m_demo` runs pretty much perfectly (depending on which version of
Octave you are using). However,
there are features that either don't work, or don't work properly. Unfortunately the rather obscure
details of different functionalities that `M_Map` relies on can change from release to release; issues that
were "fixed" may "break" in later releases. Feel free to contact me if you have problems; I may have solutions (or I may not).

Features that don't work, or don't work well when I last tested this under Octave 6.2.0, include:

1. `m_contourf`, which is really just a call to `contourf`, doesn't work correctly sometimes,
   because the Octave `contourf` does not handle `NaN`s properly, and `NaN`s can appear if data fields extend
   beyond the map boundaries and must be clipped. Even when it does work,
   it can take a VEERRRYYY LOOOONNNNGG TIIIIMMMMEEEE to run. Staggeringly long, sometimes.
  
2. Latitude and Longitude labels don't appear to rotate, even if you specify a rotation.
  
3. `m_image` won't work for projections other than equidistant cylindrical, because this requires a call to
   the MATLAB `griddedInterpolant` function.

## 13. Changes since last release

1. Moved the default location for coastline files into a >code>/data subdirectory
2. Some minor bugfixes
