# Supplementary figures and images for: Excursions in the Bayesian treatment of model error
Source: PLoS One. 2023 Jun 2;18(6):e0286624. doi: 10.1371/journal.pone.0286624 (PMC10237458; doi:10.1371/journal.pone.0286624)

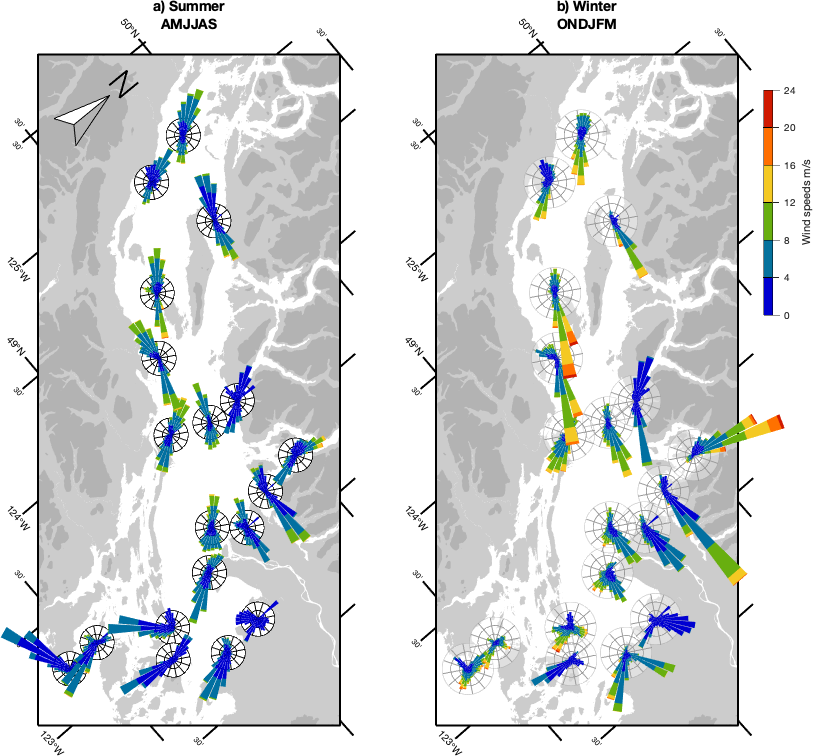

Supplement: S1 Data — The file suporting_information.zip contains the MATLAB code and the synthetic data that was used to produce all the figures in this manuscript. (ZIP) [file pone.0286624.s001.zip › Figure1/m_map/doc/exWindRose.png]

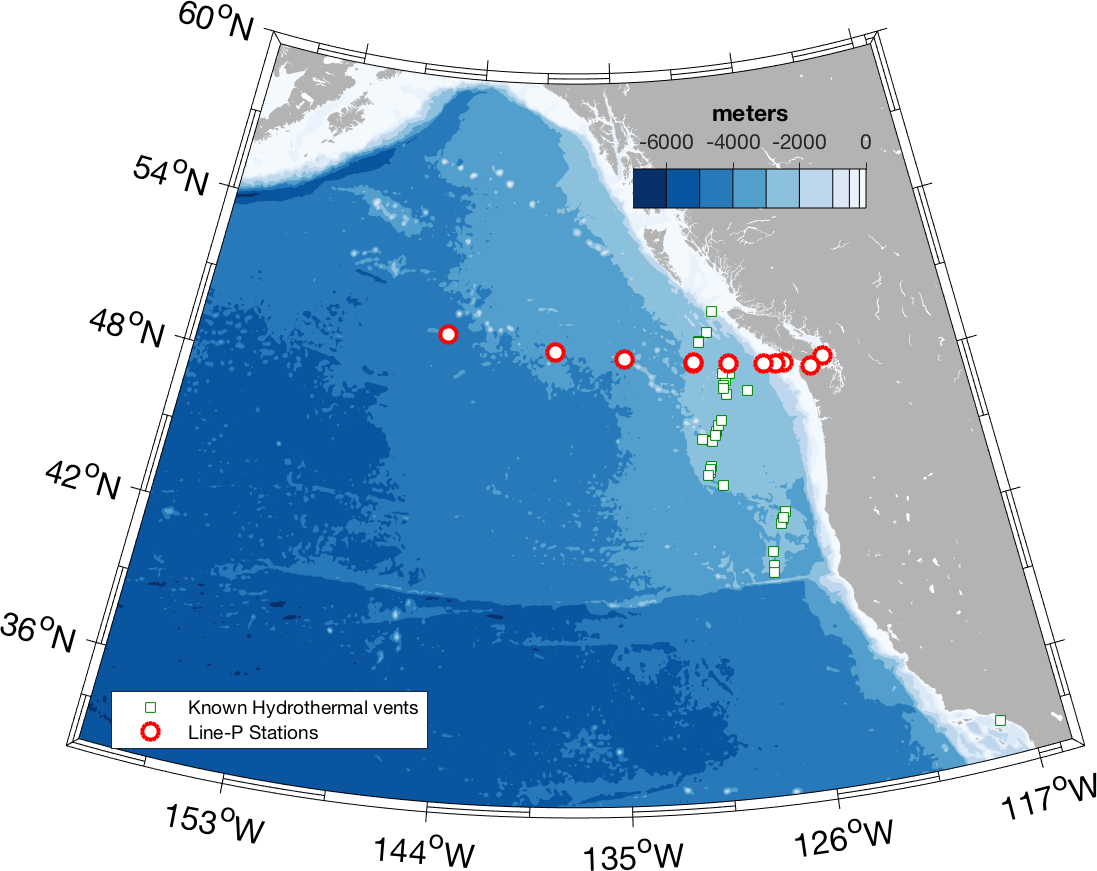

Supplement: S1 Data — The file suporting_information.zip contains the MATLAB code and the synthetic data that was used to produce all the figures in this manuscript. (ZIP) [file pone.0286624.s001.zip › Figure1/m_map/doc/exbathym.png]

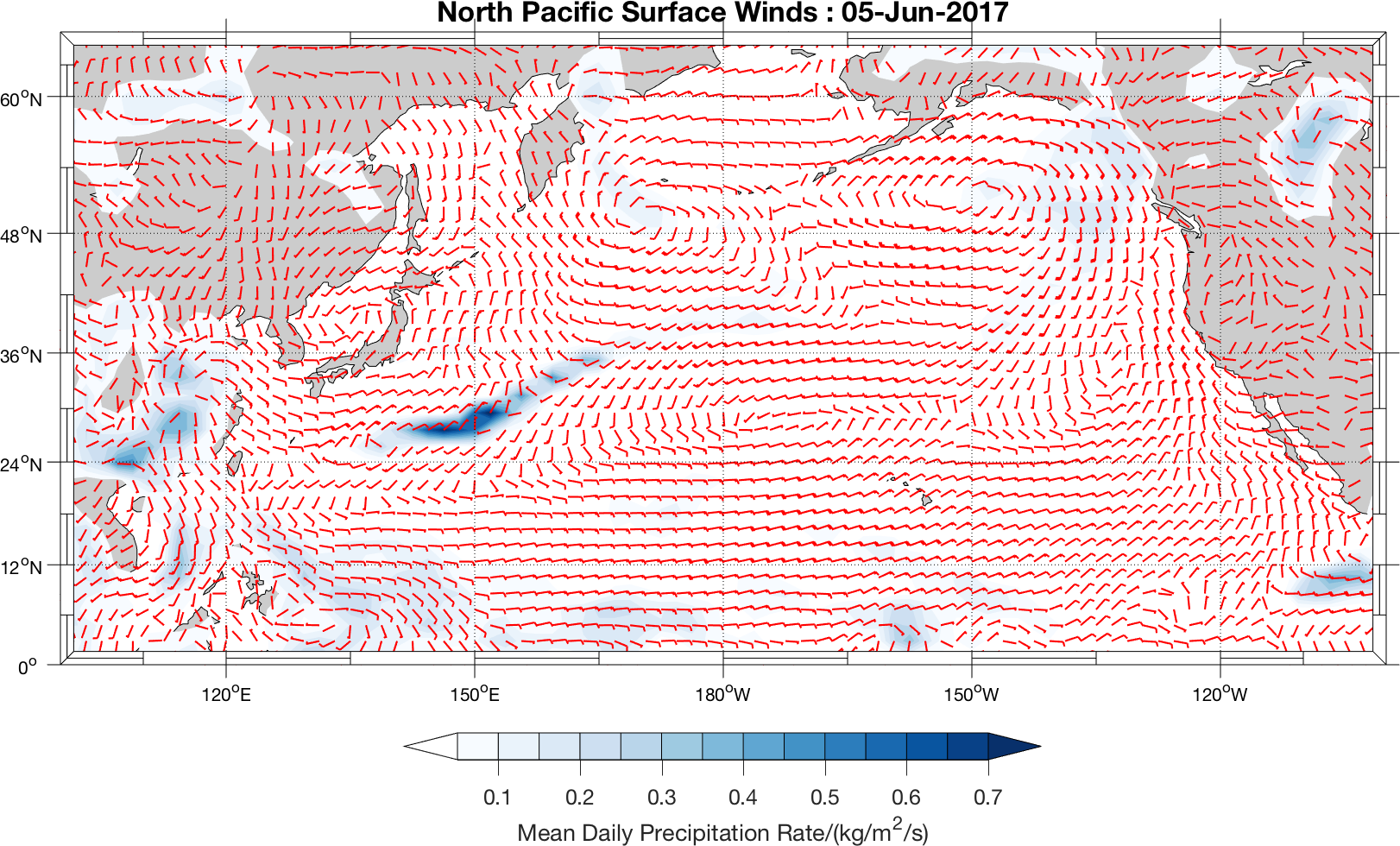

Supplement: S1 Data — The file suporting_information.zip contains the MATLAB code and the synthetic data that was used to produce all the figures in this manuscript. (ZIP) [file pone.0286624.s001.zip › Figure1/m_map/doc/exncread.png]

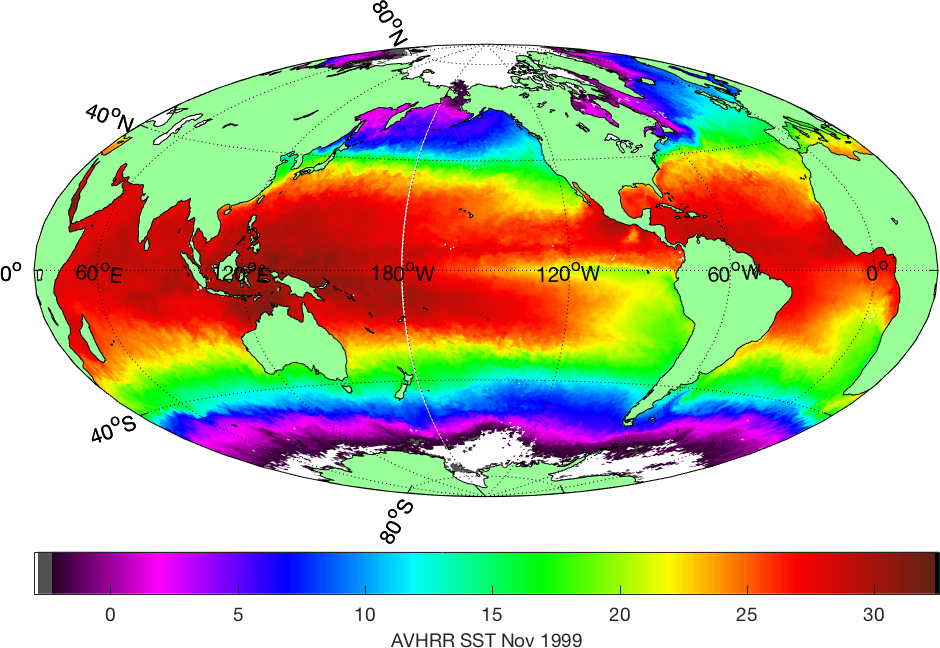

Supplement: S1 Data — The file suporting_information.zip contains the MATLAB code and the synthetic data that was used to produce all the figures in this manuscript. (ZIP) [file pone.0286624.s001.zip › Figure1/m_map/doc/exsst.png]

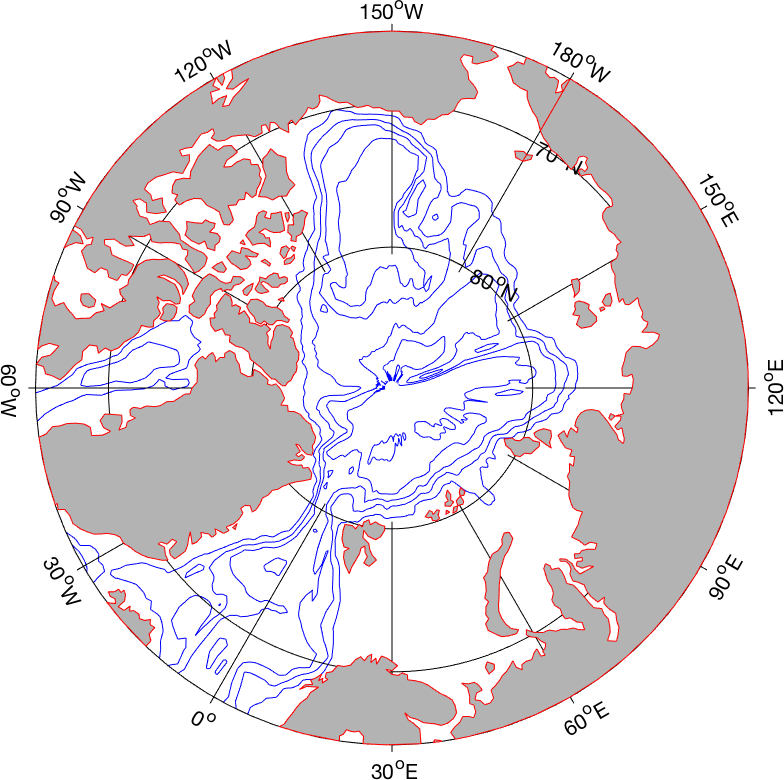

Supplement: S1 Data — The file suporting_information.zip contains the MATLAB code and the synthetic data that was used to produce all the figures in this manuscript. (ZIP) [file pone.0286624.s001.zip › Figure1/m_map/doc/exster.png]

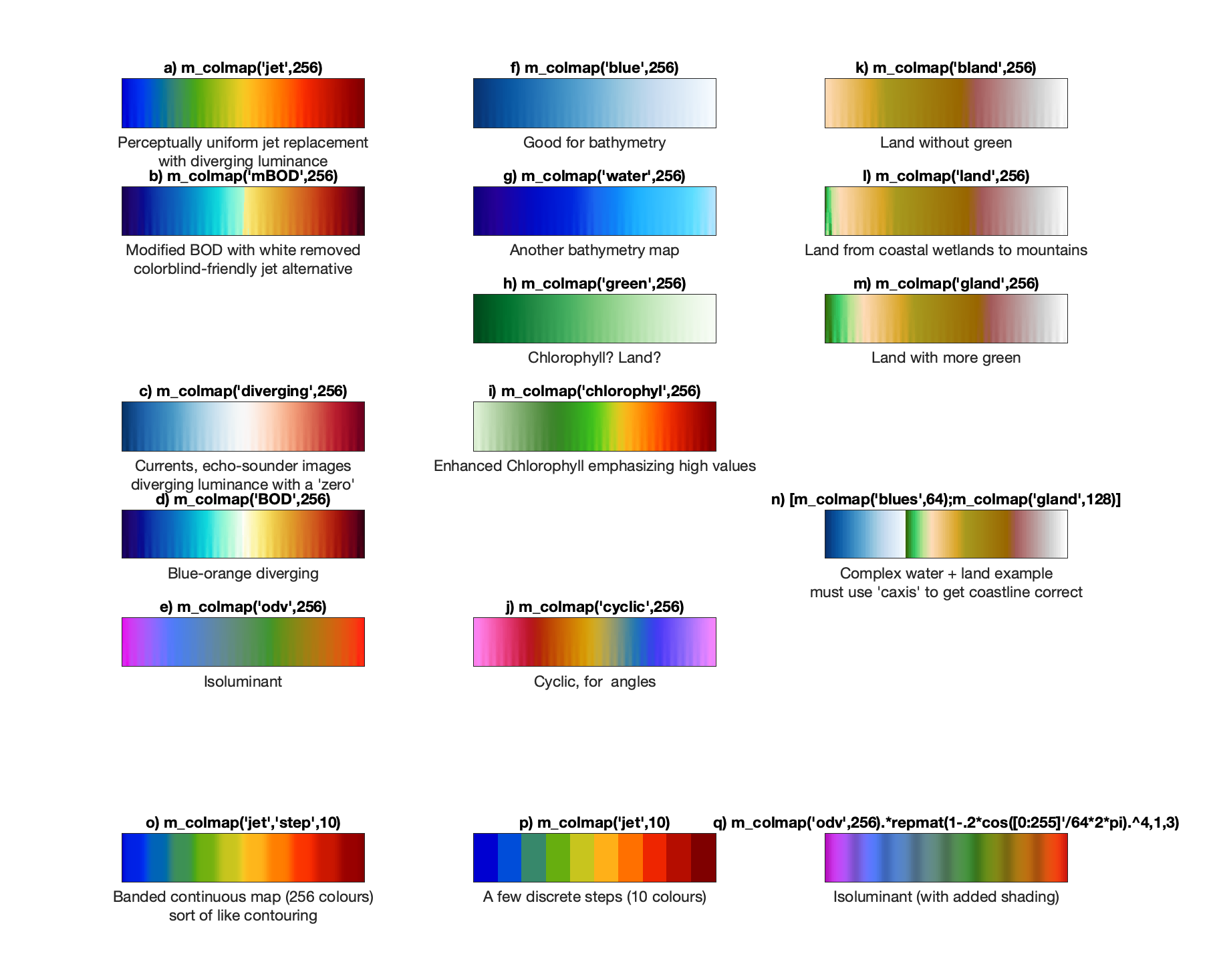

Supplement: S1 Data — The file suporting_information.zip contains the MATLAB code and the synthetic data that was used to produce all the figures in this manuscript. (ZIP) [file pone.0286624.s001.zip › Figure1/m_map/doc/exColmaps.png]

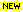

Supplement: S1 Data — The file suporting_information.zip contains the MATLAB code and the synthetic data that was used to produce all the figures in this manuscript. (ZIP) [file pone.0286624.s001.zip › Figure1/m_map/doc/new.gif]

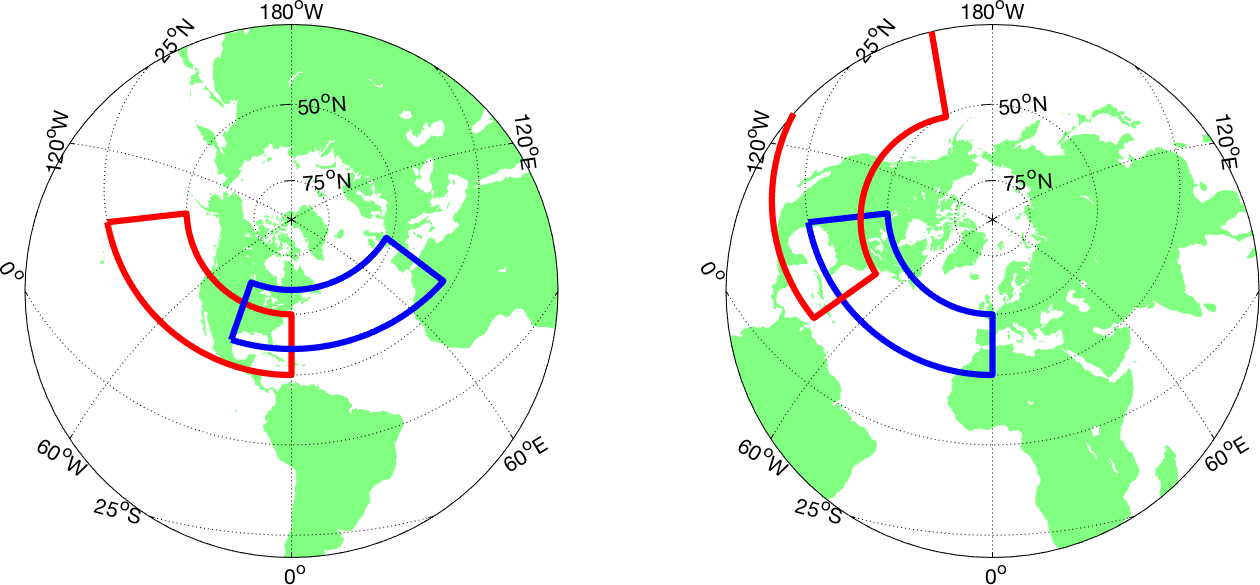

Supplement: S1 Data — The file suporting_information.zip contains the MATLAB code and the synthetic data that was used to produce all the figures in this manuscript. (ZIP) [file pone.0286624.s001.zip › Figure1/m_map/doc/exMAG.png]

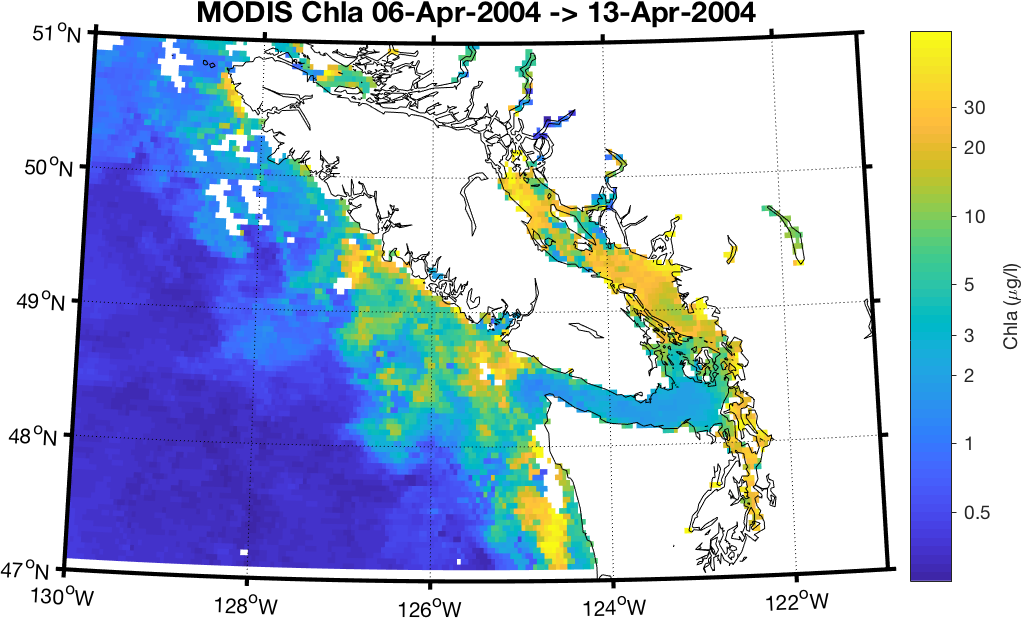

Supplement: S1 Data — The file suporting_information.zip contains the MATLAB code and the synthetic data that was used to produce all the figures in this manuscript. (ZIP) [file pone.0286624.s001.zip › Figure1/m_map/doc/exmodis.png]

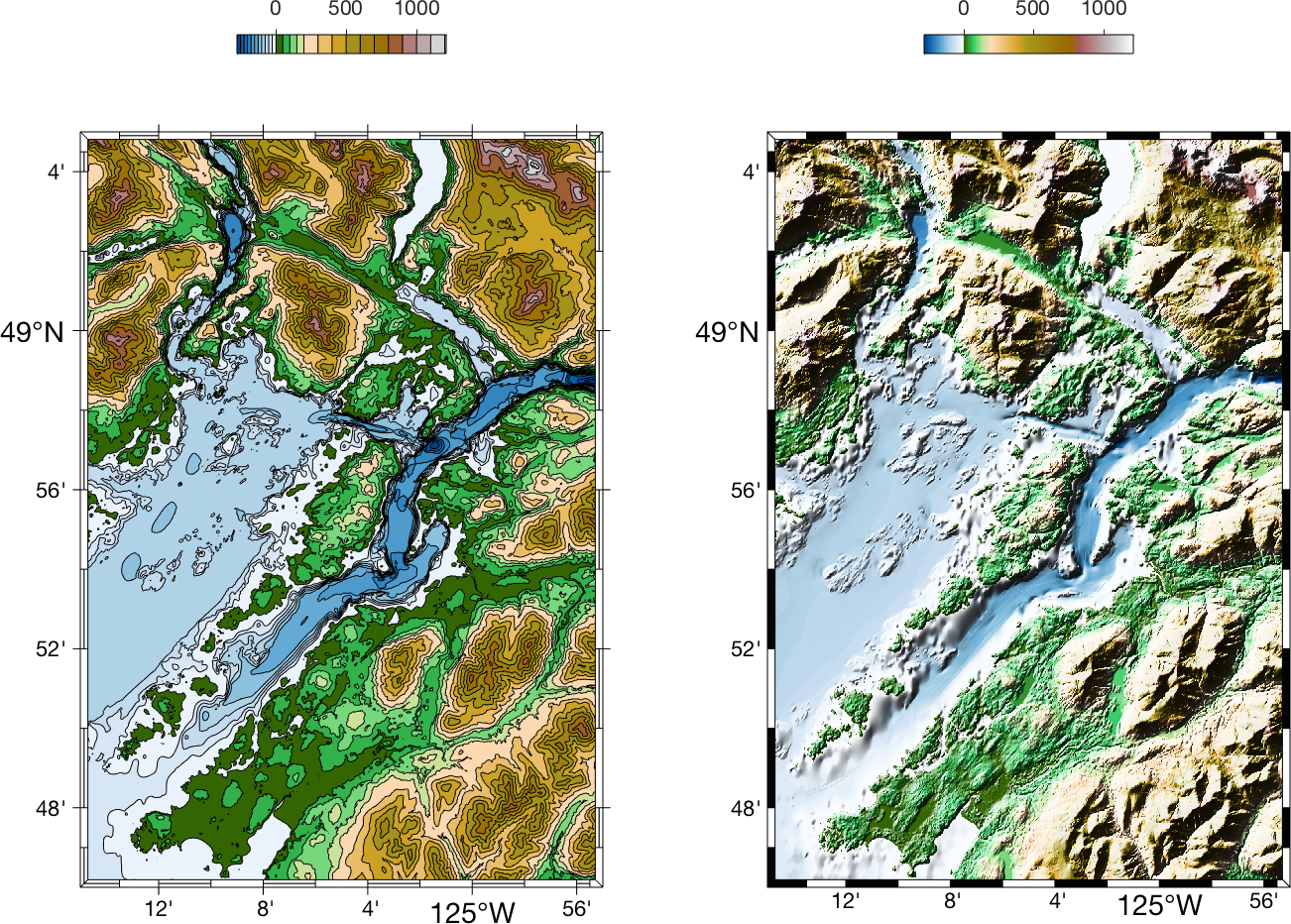

Supplement: S1 Data — The file suporting_information.zip contains the MATLAB code and the synthetic data that was used to produce all the figures in this manuscript. (ZIP) [file pone.0286624.s001.zip › Figure1/m_map/doc/shaded1.png]

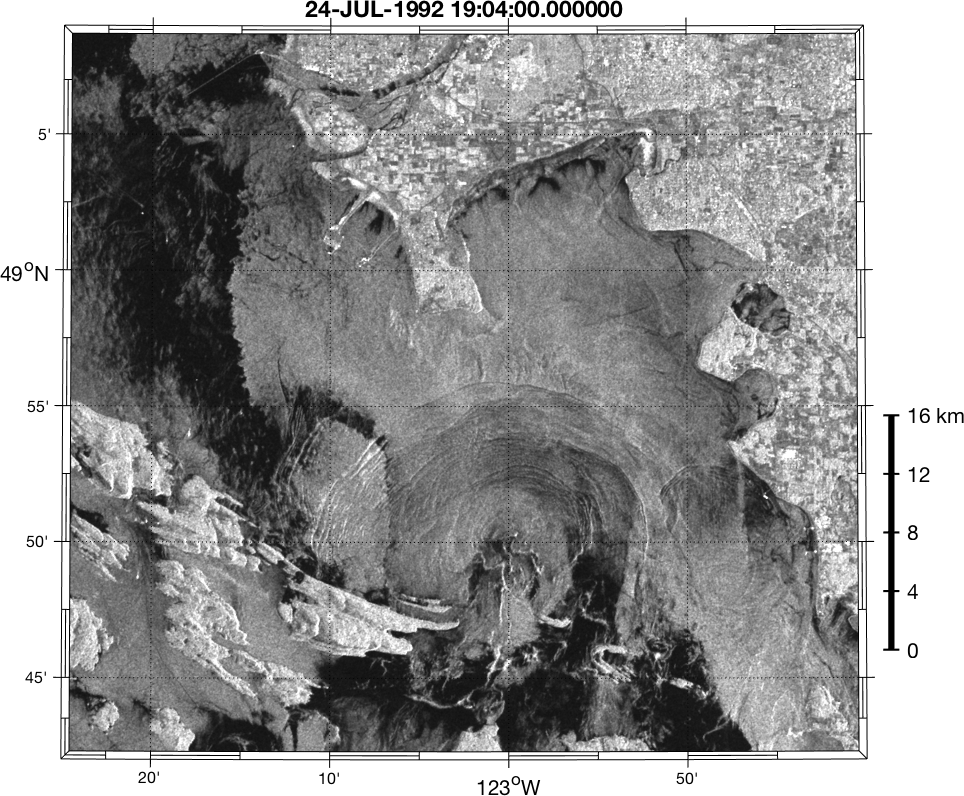

Supplement: S1 Data — The file suporting_information.zip contains the MATLAB code and the synthetic data that was used to produce all the figures in this manuscript. (ZIP) [file pone.0286624.s001.zip › Figure1/m_map/doc/exSAR.png]

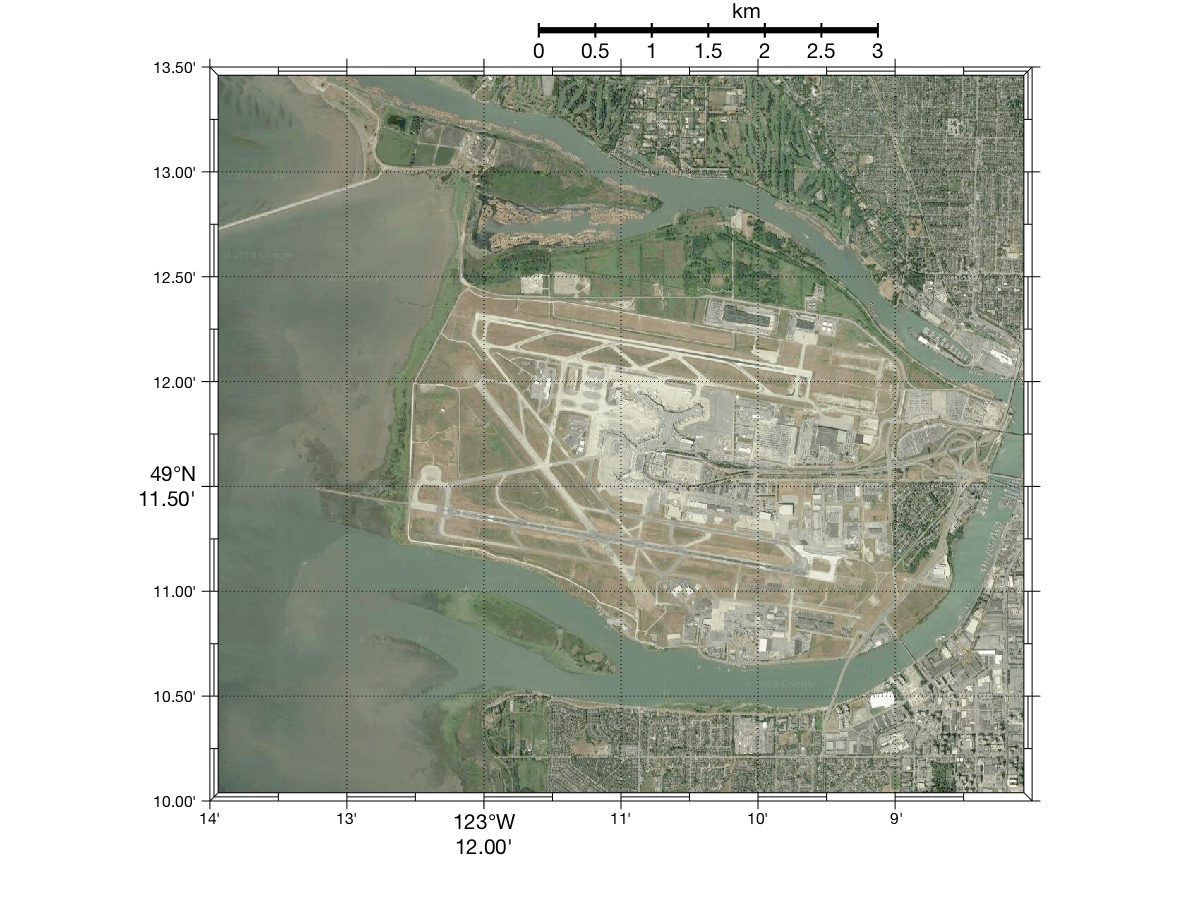

Supplement: S1 Data — The file suporting_information.zip contains the MATLAB code and the synthetic data that was used to produce all the figures in this manuscript. (ZIP) [file pone.0286624.s001.zip › Figure1/m_map/doc/exgoog.png]

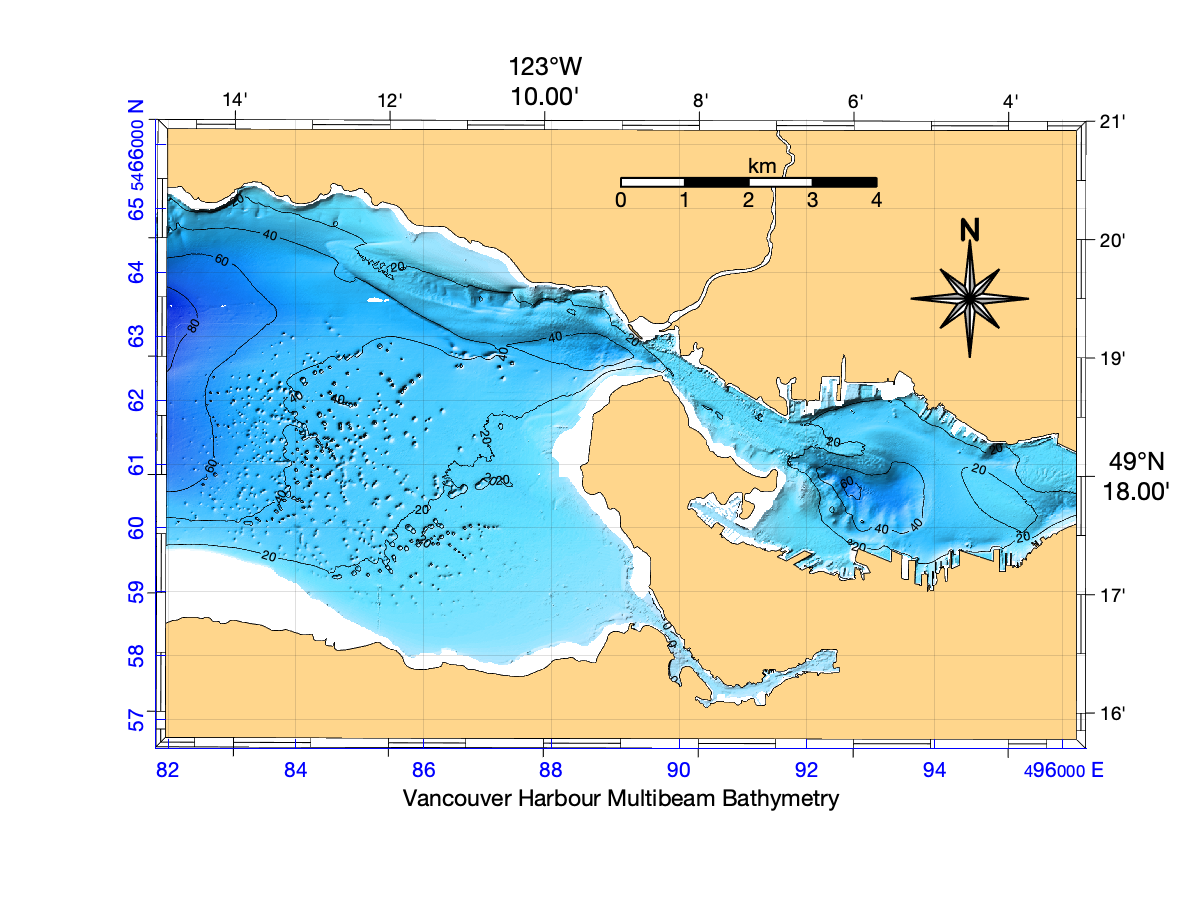

Supplement: S1 Data — The file suporting_information.zip contains the MATLAB code and the synthetic data that was used to produce all the figures in this manuscript. (ZIP) [file pone.0286624.s001.zip › Figure1/m_map/doc/VanHarb.png]

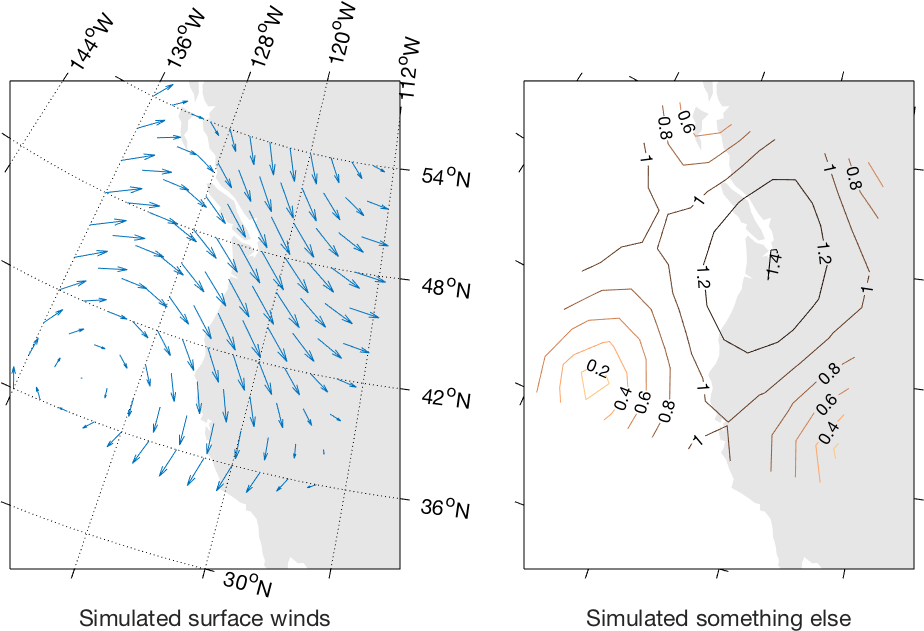

Supplement: S1 Data — The file suporting_information.zip contains the MATLAB code and the synthetic data that was used to produce all the figures in this manuscript. (ZIP) [file pone.0286624.s001.zip › Figure1/m_map/doc/exquiv.png]

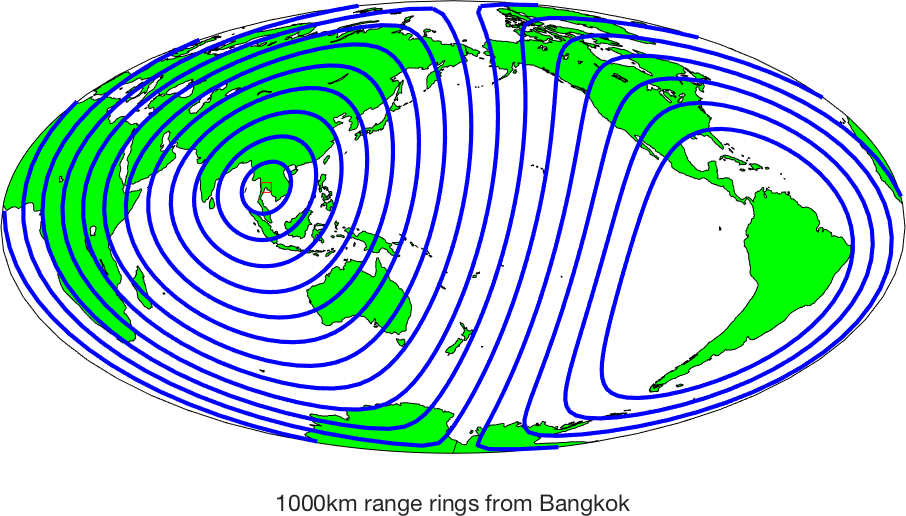

Supplement: S1 Data — The file suporting_information.zip contains the MATLAB code and the synthetic data that was used to produce all the figures in this manuscript. (ZIP) [file pone.0286624.s001.zip › Figure1/m_map/doc/exrring.png]

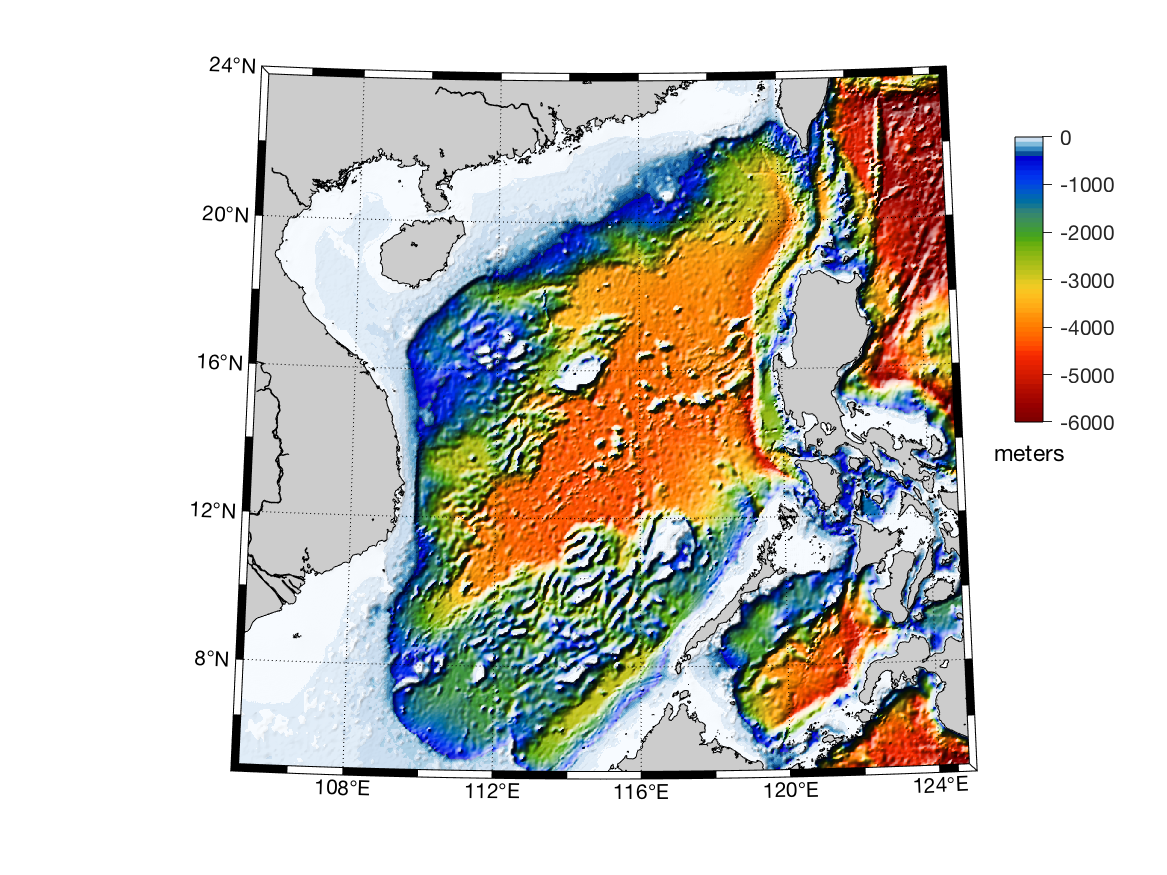

Supplement: S1 Data — The file suporting_information.zip contains the MATLAB code and the synthetic data that was used to produce all the figures in this manuscript. (ZIP) [file pone.0286624.s001.zip › Figure1/m_map/doc/SouthChinaSea.png]

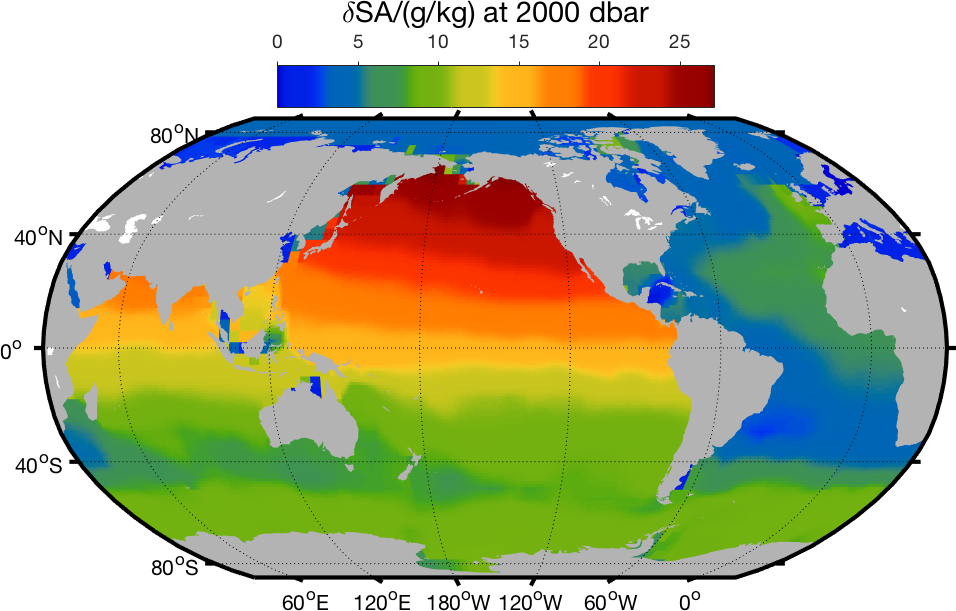

Supplement: S1 Data — The file suporting_information.zip contains the MATLAB code and the synthetic data that was used to produce all the figures in this manuscript. (ZIP) [file pone.0286624.s001.zip › Figure1/m_map/doc/exstepjet.png]

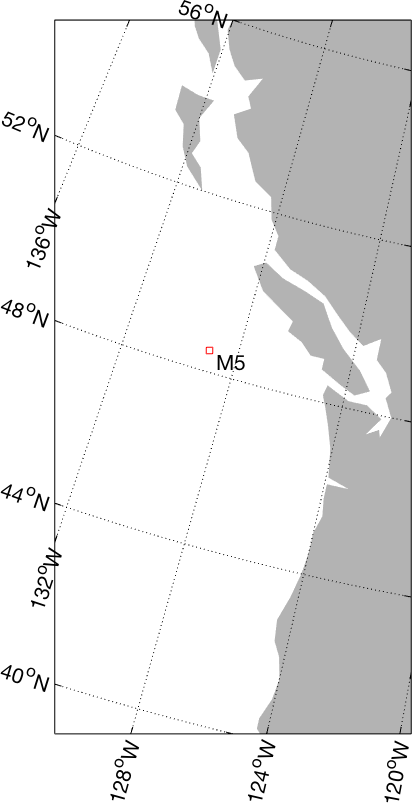

Supplement: S1 Data — The file suporting_information.zip contains the MATLAB code and the synthetic data that was used to produce all the figures in this manuscript. (ZIP) [file pone.0286624.s001.zip › Figure1/m_map/doc/exobl2.png]

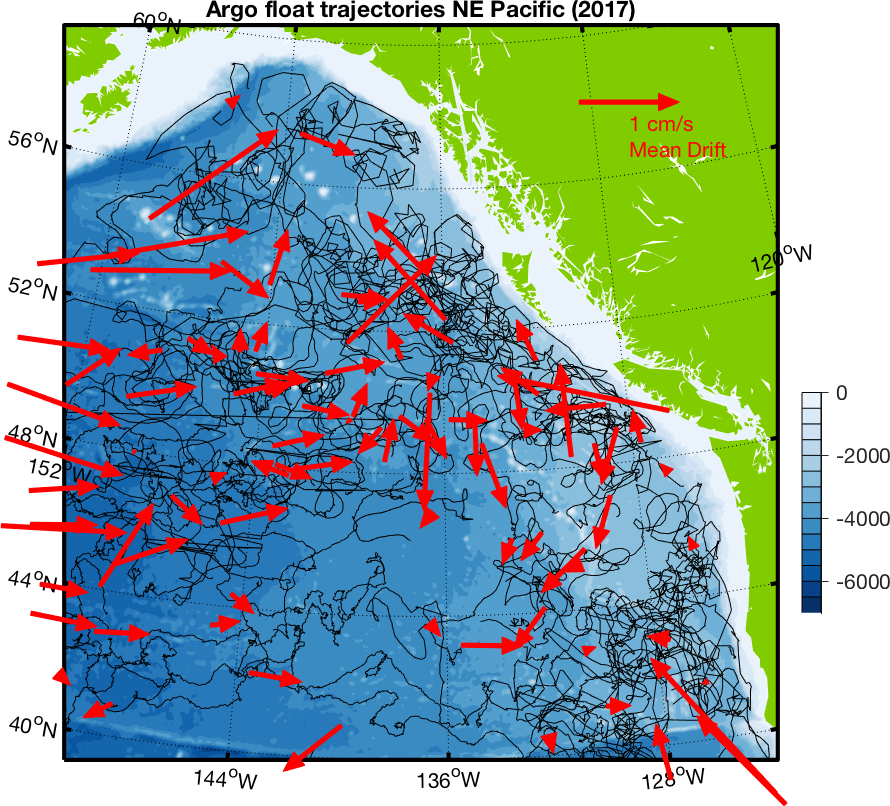

Supplement: S1 Data — The file suporting_information.zip contains the MATLAB code and the synthetic data that was used to produce all the figures in this manuscript. (ZIP) [file pone.0286624.s001.zip › Figure1/m_map/doc/exargo.png]

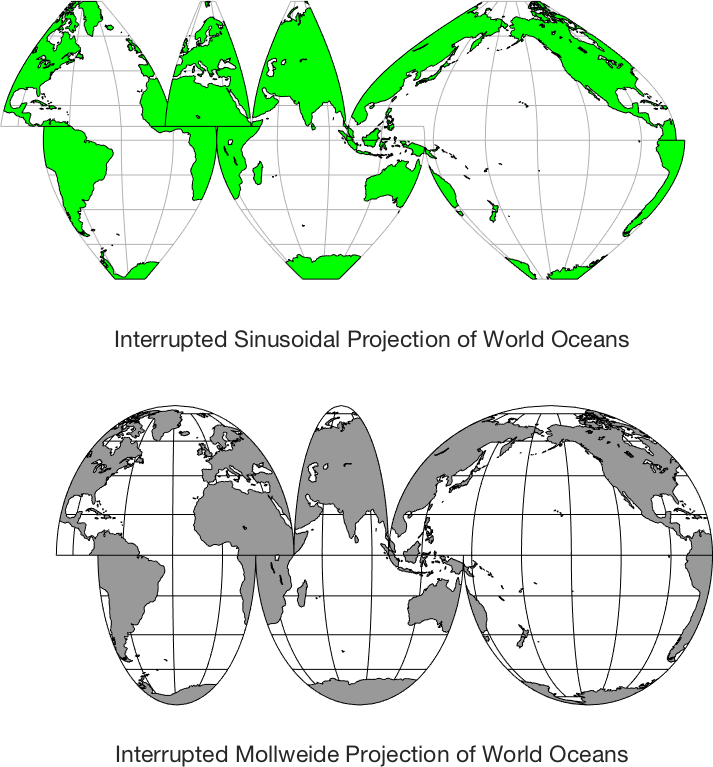

Supplement: S1 Data — The file suporting_information.zip contains the MATLAB code and the synthetic data that was used to produce all the figures in this manuscript. (ZIP) [file pone.0286624.s001.zip › Figure1/m_map/doc/exsinus.png]

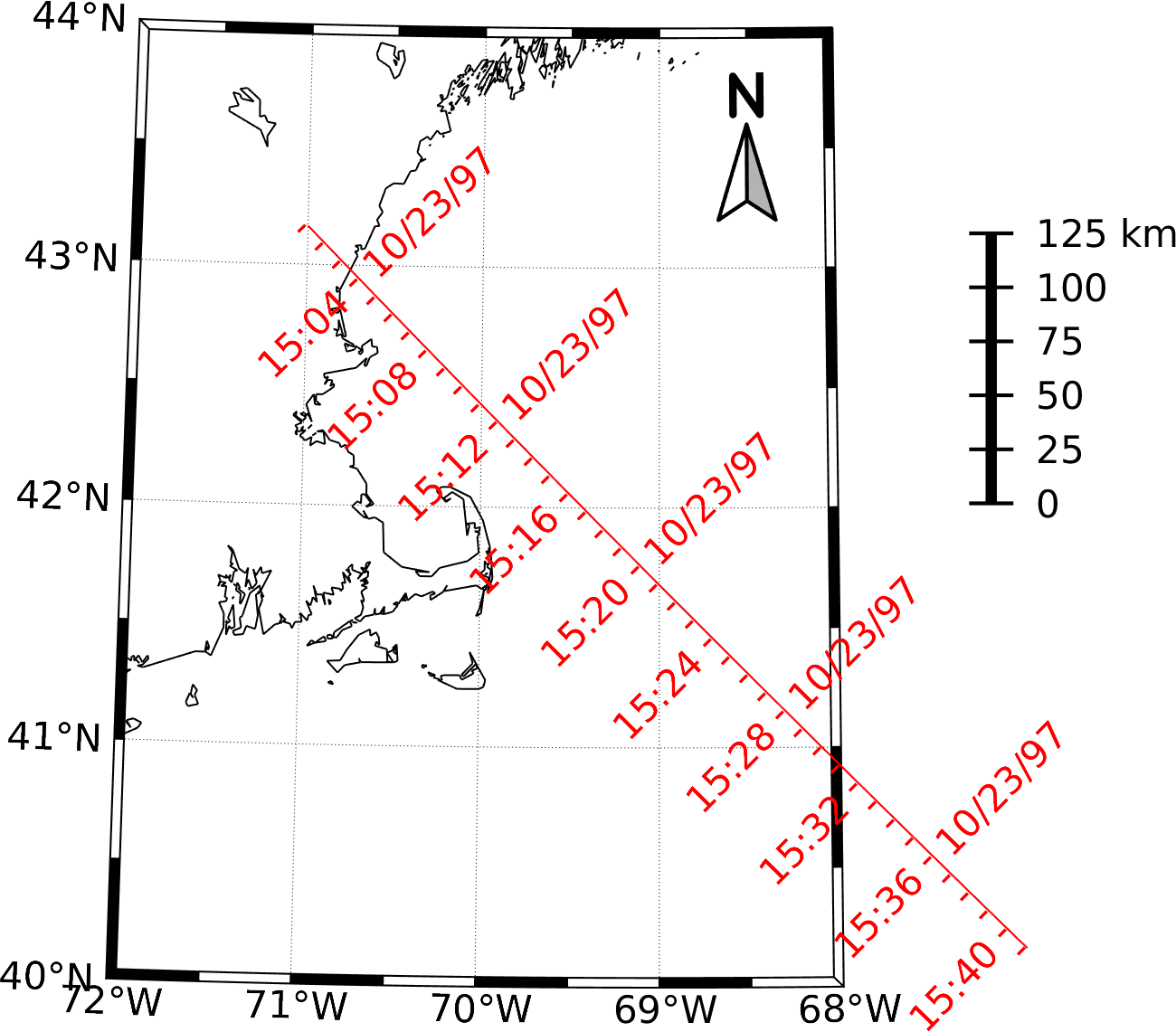

Supplement: S1 Data — The file suporting_information.zip contains the MATLAB code and the synthetic data that was used to produce all the figures in this manuscript. (ZIP) [file pone.0286624.s001.zip › Figure1/m_map/doc/extrack1.png]

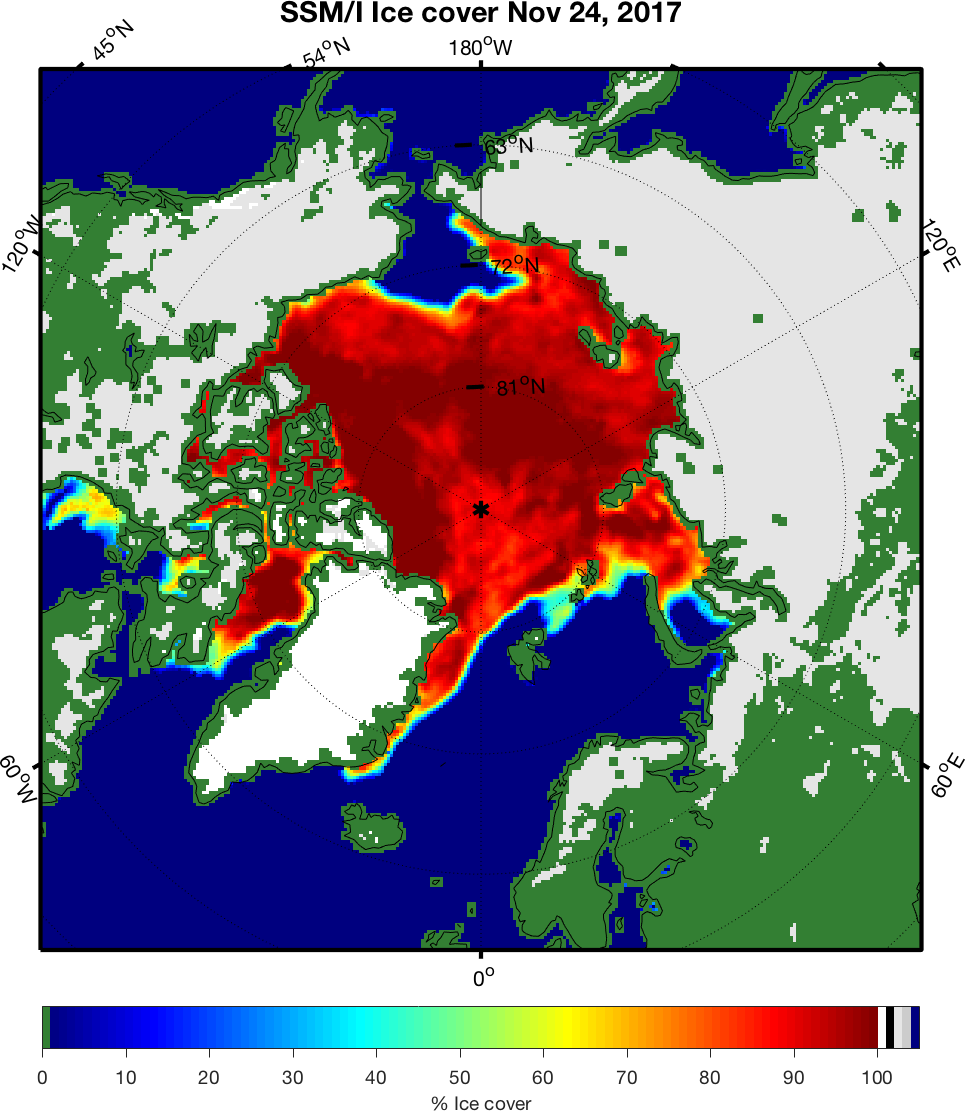

Supplement: S1 Data — The file suporting_information.zip contains the MATLAB code and the synthetic data that was used to produce all the figures in this manuscript. (ZIP) [file pone.0286624.s001.zip › Figure1/m_map/doc/ex_ssmi2.png]

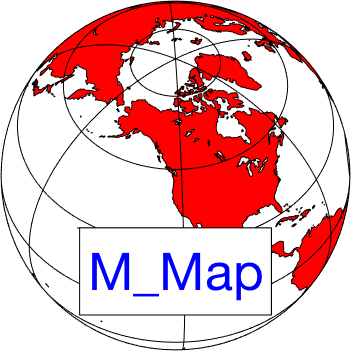

Supplement: S1 Data — The file suporting_information.zip contains the MATLAB code and the synthetic data that was used to produce all the figures in this manuscript. (ZIP) [file pone.0286624.s001.zip › Figure1/m_map/doc/mlogo.png]

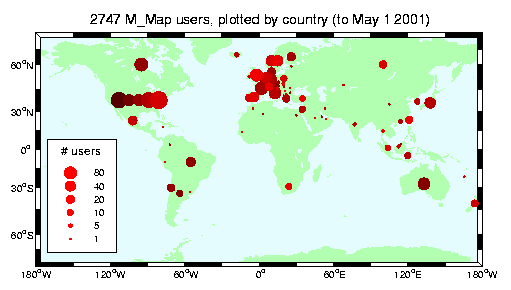

Supplement: S1 Data — The file suporting_information.zip contains the MATLAB code and the synthetic data that was used to produce all the figures in this manuscript. (ZIP) [file pone.0286624.s001.zip › Figure1/m_map/doc/usermap.gif]

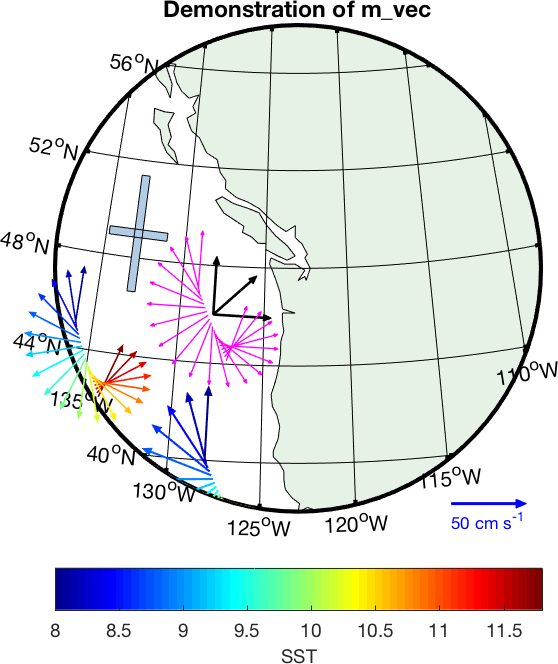

Supplement: S1 Data — The file suporting_information.zip contains the MATLAB code and the synthetic data that was used to produce all the figures in this manuscript. (ZIP) [file pone.0286624.s001.zip › Figure1/m_map/doc/exvec.png]

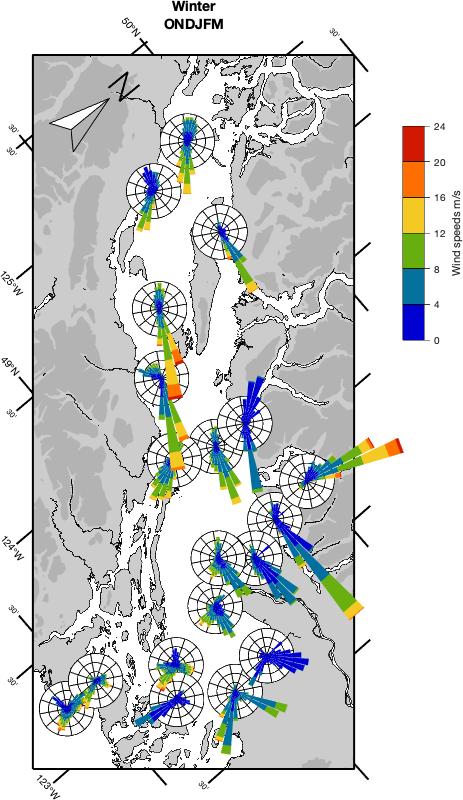

Supplement: S1 Data — The file suporting_information.zip contains the MATLAB code and the synthetic data that was used to produce all the figures in this manuscript. (ZIP) [file pone.0286624.s001.zip › Figure1/m_map/doc/exWindRose2.png]

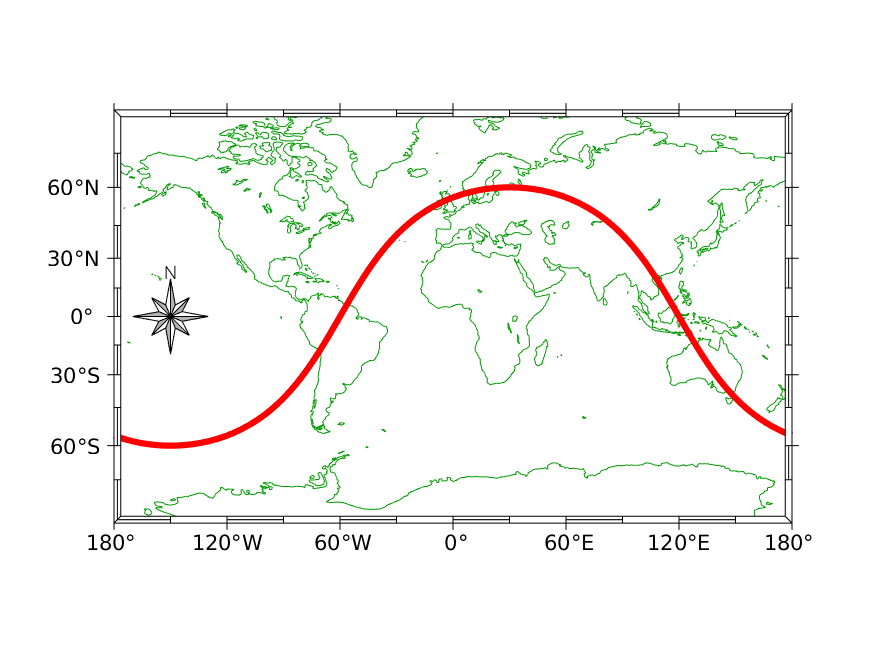

Supplement: S1 Data — The file suporting_information.zip contains the MATLAB code and the synthetic data that was used to produce all the figures in this manuscript. (ZIP) [file pone.0286624.s001.zip › Figure1/m_map/doc/exmiller.png]

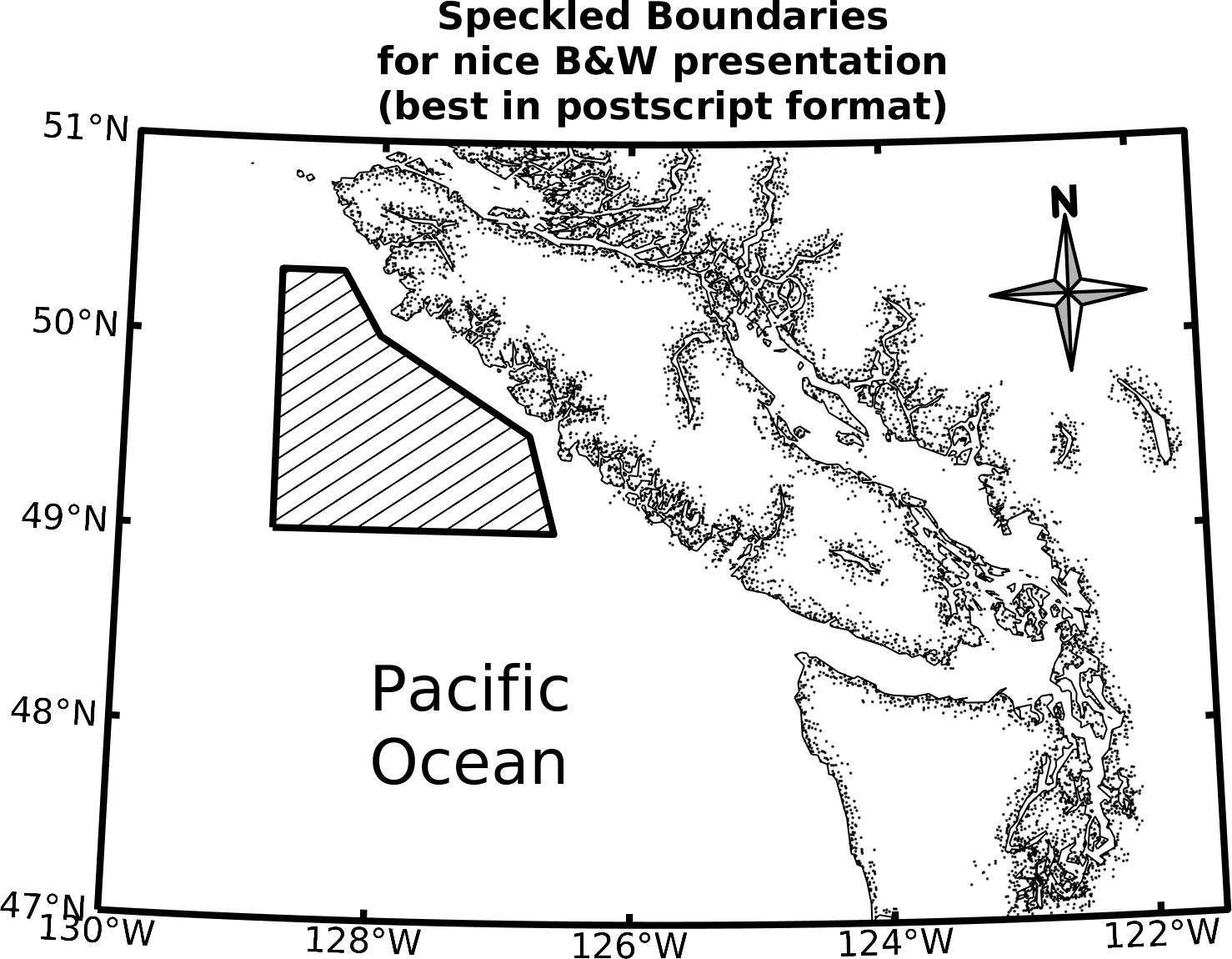

Supplement: S1 Data — The file suporting_information.zip contains the MATLAB code and the synthetic data that was used to produce all the figures in this manuscript. (ZIP) [file pone.0286624.s001.zip › Figure1/m_map/doc/exspeckle.png]

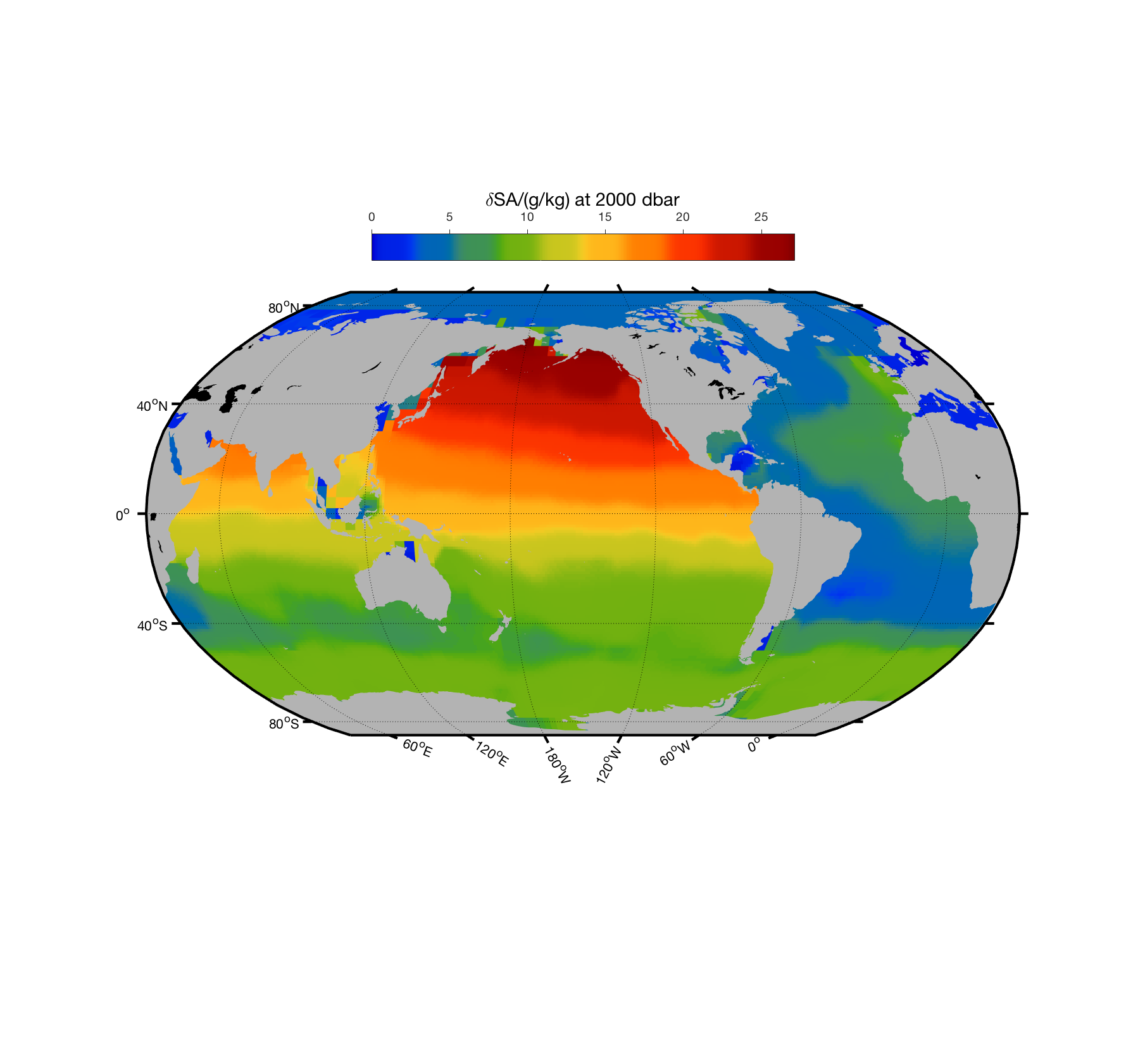

Supplement: S1 Data — The file suporting_information.zip contains the MATLAB code and the synthetic data that was used to produce all the figures in this manuscript. (ZIP) [file pone.0286624.s001.zip › Figure1/m_map/doc/stepjet.png]

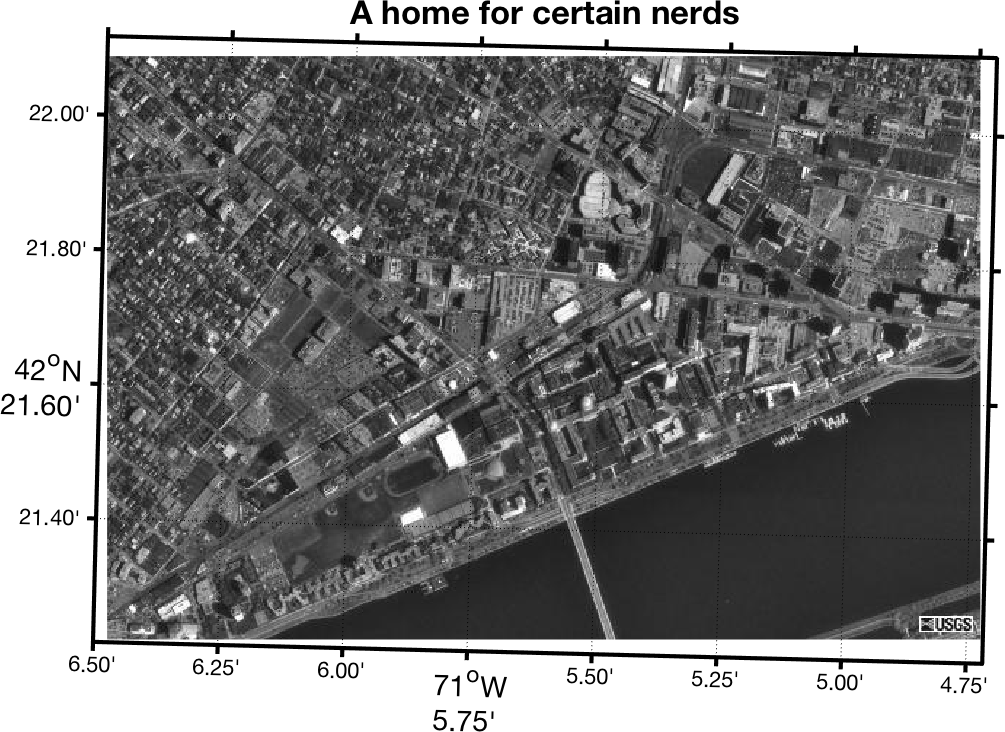

Supplement: S1 Data — The file suporting_information.zip contains the MATLAB code and the synthetic data that was used to produce all the figures in this manuscript. (ZIP) [file pone.0286624.s001.zip › Figure1/m_map/doc/exterra.png]

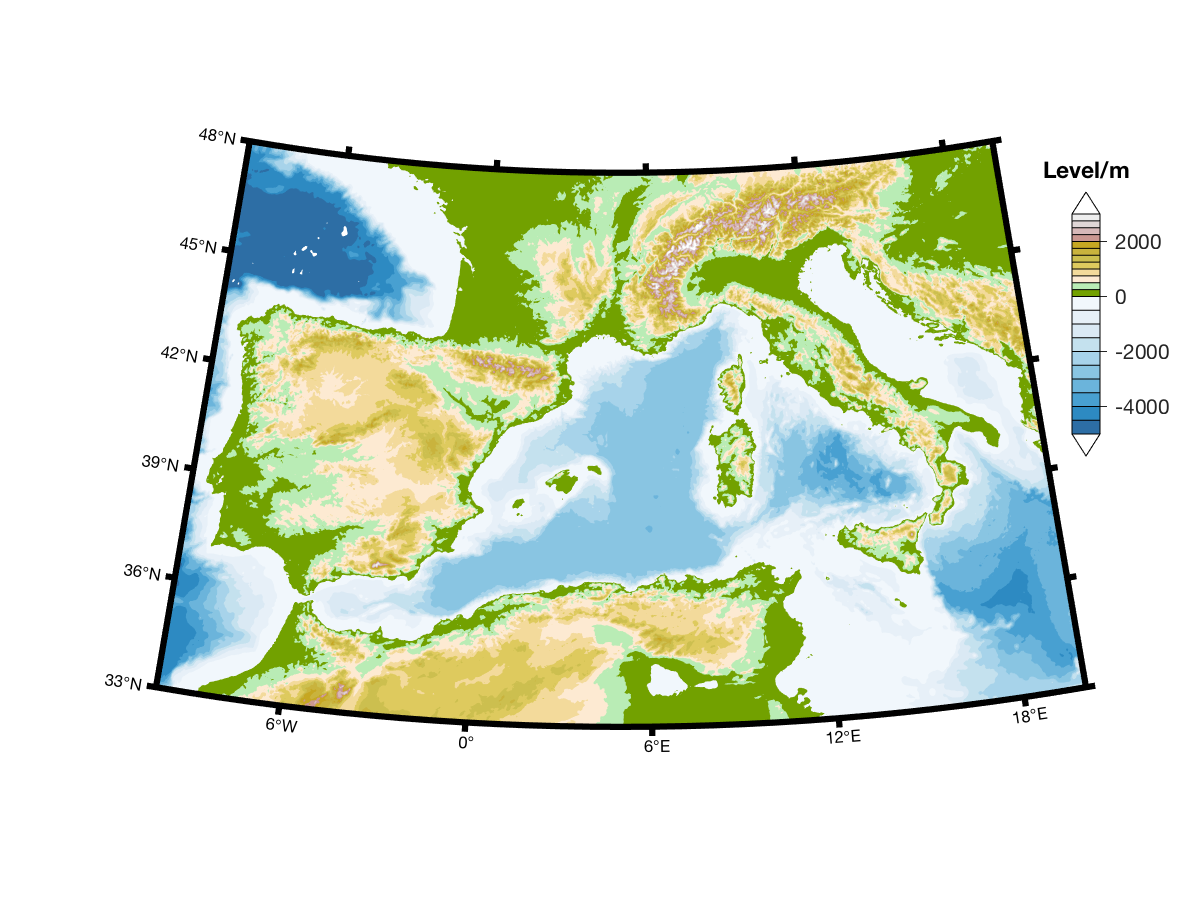

Supplement: S1 Data — The file suporting_information.zip contains the MATLAB code and the synthetic data that was used to produce all the figures in this manuscript. (ZIP) [file pone.0286624.s001.zip › Figure1/m_map/doc/extbase.png]

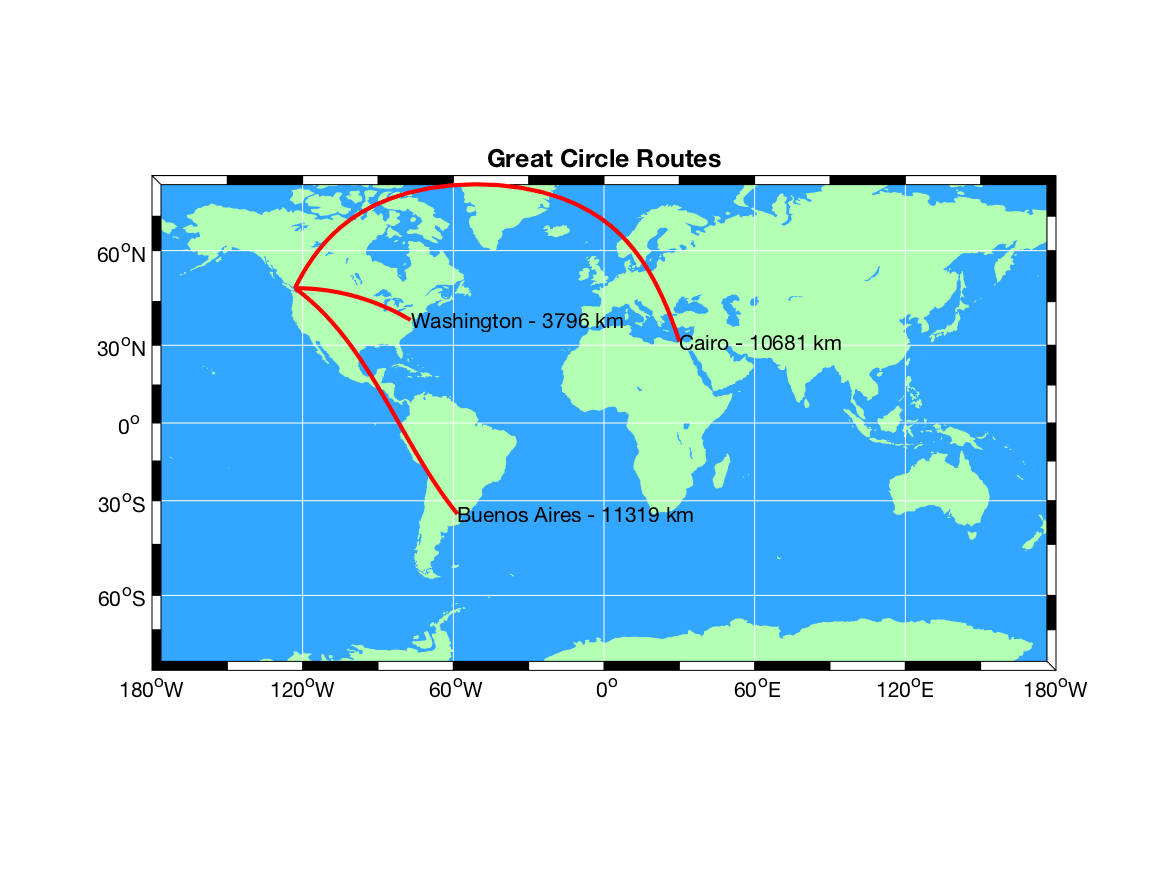

Supplement: S1 Data — The file suporting_information.zip contains the MATLAB code and the synthetic data that was used to produce all the figures in this manuscript. (ZIP) [file pone.0286624.s001.zip › Figure1/m_map/doc/exblueT.png]

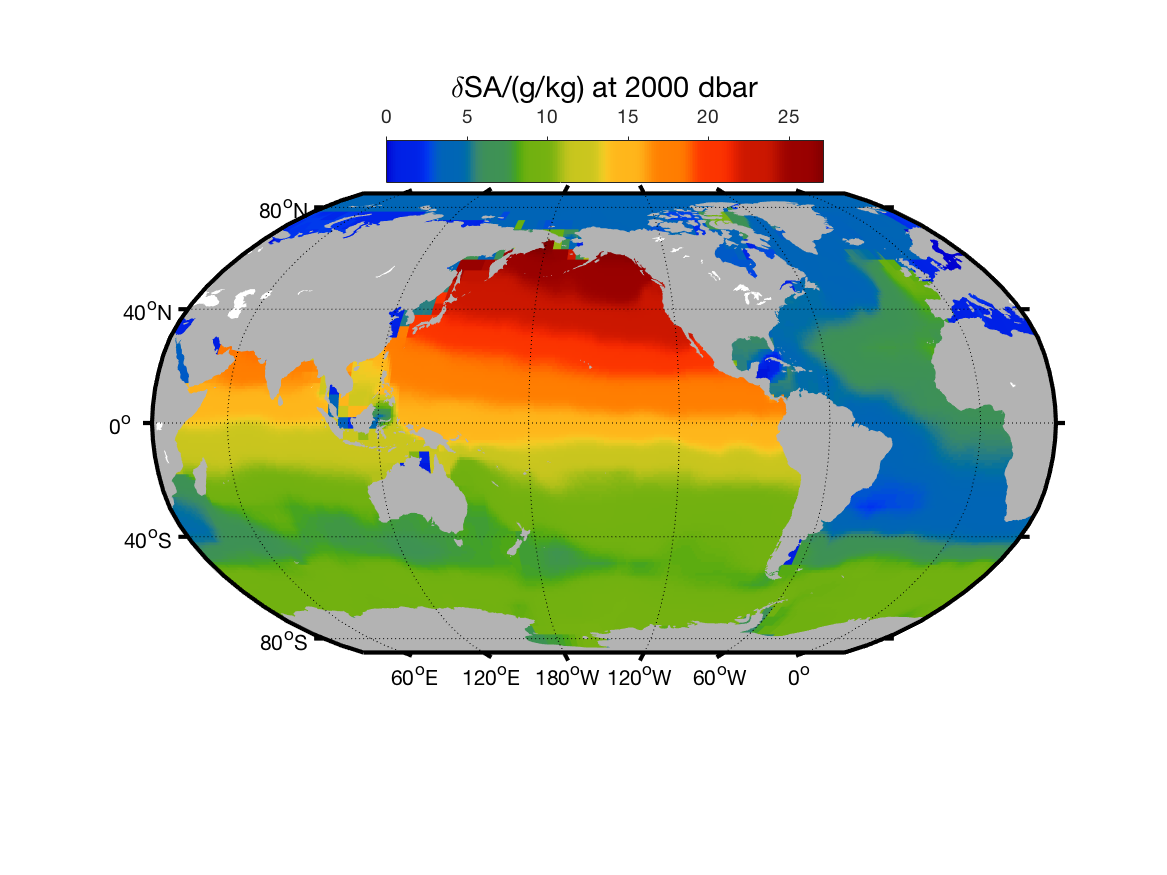

Supplement: S1 Data — The file suporting_information.zip contains the MATLAB code and the synthetic data that was used to produce all the figures in this manuscript. (ZIP) [file pone.0286624.s001.zip › Figure1/m_map/doc/exstepjetT.png]

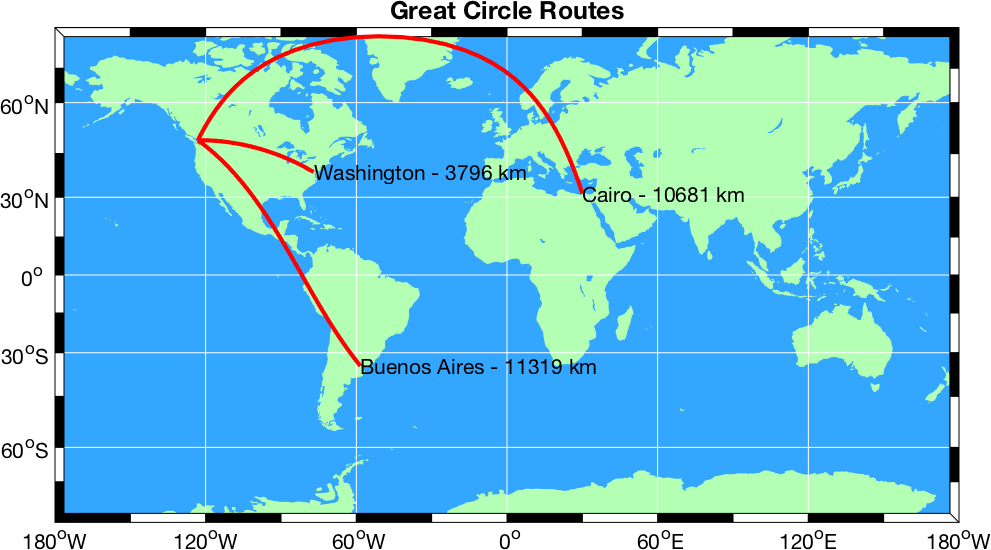

Supplement: S1 Data — The file suporting_information.zip contains the MATLAB code and the synthetic data that was used to produce all the figures in this manuscript. (ZIP) [file pone.0286624.s001.zip › Figure1/m_map/doc/exblueocean.png]

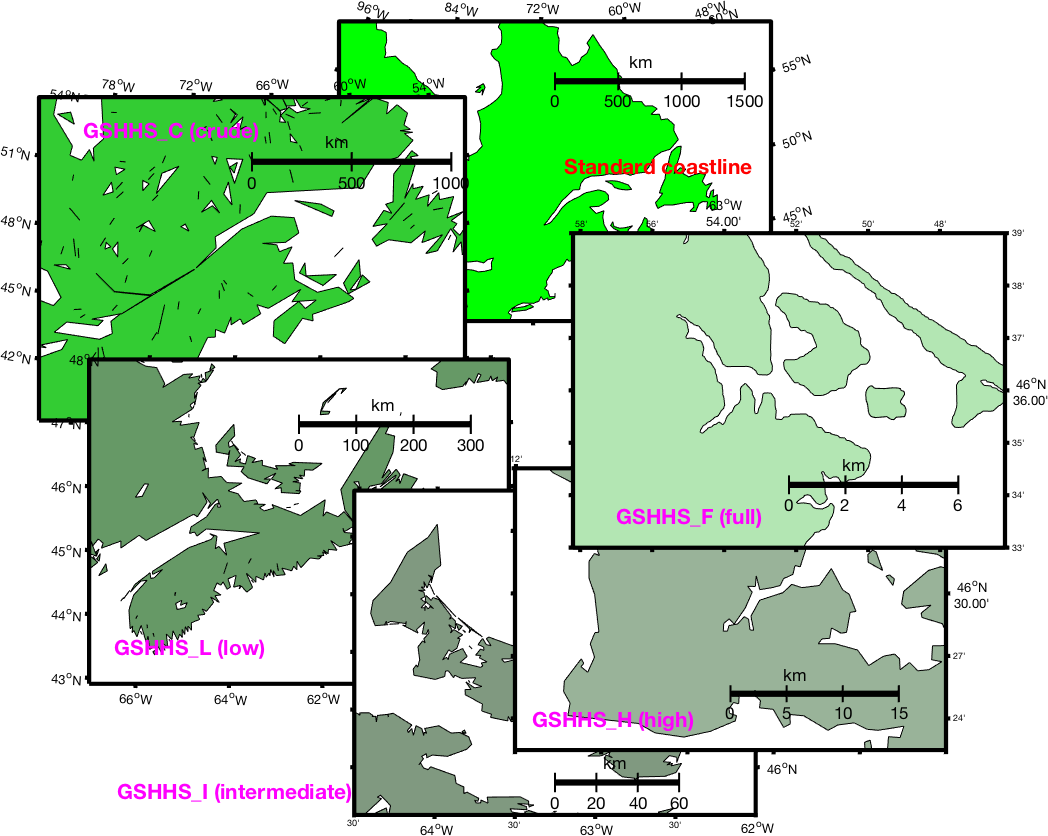

Supplement: S1 Data — The file suporting_information.zip contains the MATLAB code and the synthetic data that was used to produce all the figures in this manuscript. (ZIP) [file pone.0286624.s001.zip › Figure1/m_map/doc/exgshhs.png]

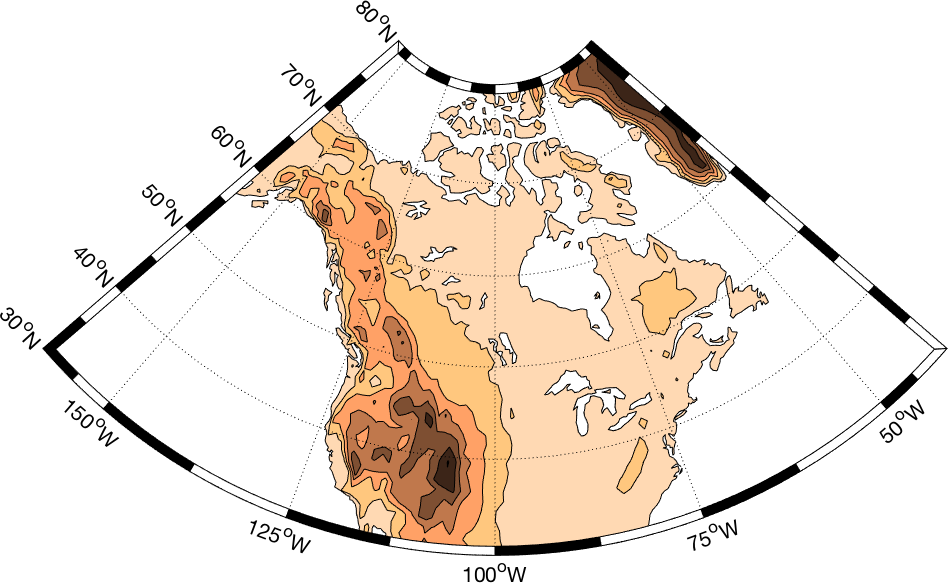

Supplement: S1 Data — The file suporting_information.zip contains the MATLAB code and the synthetic data that was used to produce all the figures in this manuscript. (ZIP) [file pone.0286624.s001.zip › Figure1/m_map/doc/exlamber.png]

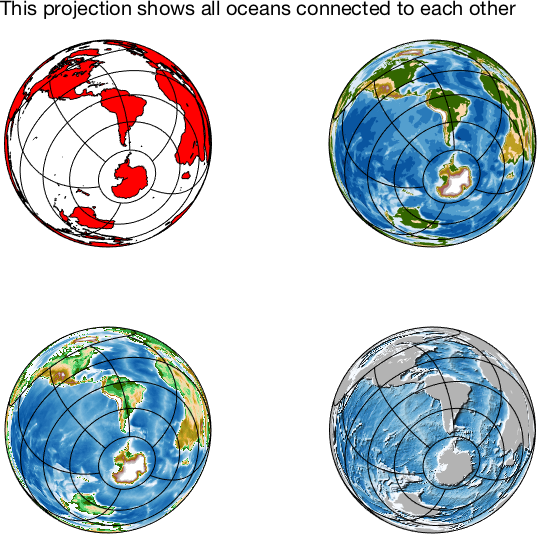

Supplement: S1 Data — The file suporting_information.zip contains the MATLAB code and the synthetic data that was used to produce all the figures in this manuscript. (ZIP) [file pone.0286624.s001.zip › Figure1/m_map/doc/extgloball.png]
